# Insights into the Small Molecule Targeting of Biologically Relevant G-Quadruplexes: An Overview of NMR and Crystal Structures

**DOI:** 10.3390/pharmaceutics14112361

**Published:** 2022-11-01

**Authors:** Andrea Criscuolo, Ettore Napolitano, Claudia Riccardi, Domenica Musumeci, Chiara Platella, Daniela Montesarchio

**Affiliations:** 1 Department of Chemical Sciences, University of Naples Federico II, Via Cintia 21, 80126 Naples, Italy; 2 Institute of Biostructures and Bioimages, CNR, 80134 Naples, Italy

**Keywords:** cancer, crystallography, G-quadruplex, ligand, NMR spectroscopy

## Abstract

G-quadruplexes turned out to be important targets for the development of novel targeted anticancer/antiviral therapies. More than 3000 G-quadruplex small-molecule ligands have been described, with most of them exerting anticancer/antiviral activity by inducing telomeric damage and/or altering oncogene or viral gene expression in cancer cells and viruses, respectively. For some ligands, in-depth NMR and/or crystallographic studies were performed, providing detailed knowledge on their interactions with diverse G-quadruplex targets. Here, the PDB-deposited NMR and crystal structures of the complexes between telomeric, oncogenic or viral G-quadruplexes and small-molecule ligands, of both organic and metal-organic nature, have been summarized and described based on the G-quadruplex target, from telomeric DNA and RNA G-quadruplexes to DNA oncogenic G-quadruplexes, and finally to RNA viral G-quadruplexes. An overview of the structural details of these complexes is here provided to guide the design of novel ligands targeting more efficiently and selectively cancer- and virus-related G-quadruplex structures.

## 1. Introduction

The study of small-molecule ligands specifically binding and stabilizing G-quadruplex nucleic acid structures is increasingly emerging as a promising branch of targeted anticancer research, due to the relevant roles played by these structures in the regulation of specific pathways of cancer cells [1,2,3,4].

**Figure 1:**
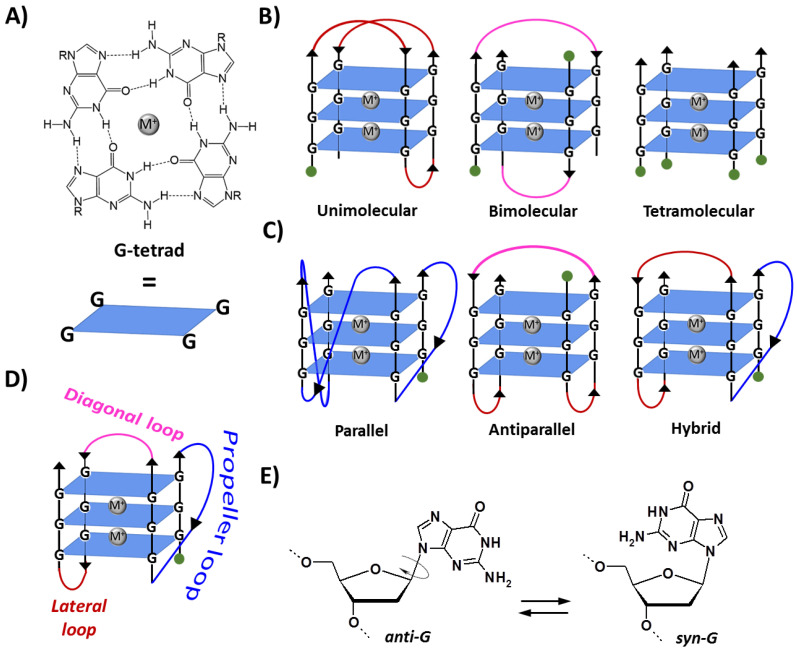
(**A**) Structure and schematic representation of a G-tetrad. (**B**) Schematic representation of unimolecular, bimolecular and tetramolecular G-quadruplexes formed by the stacking of three G-tetrads. (**C**) Examples of three different topologies of unimolecular G-quadruplexes. (**D**) Types of linking loops in a G-quadruplex. (**E**) The *anti*/*syn*-conformations of 2′-deoxyguanosine. M^+^ indicates a stabilizing metal cation, e.g., K^+^ or Na^+^. R = 1-β-D-2-deoxyribofuranosyl group. Green circles indicate the 5′-end. Adapted with permission from ref. [9], Copyright 2022 The Authors. Published by Wiley-VCH GmbH.

G-quadruplexes are noncanonical DNA and RNA structures formed by G-rich sequences in which four guanine bases associate, through Hoogsteen hydrogen bonds, in a coplanar arrangement named G-tetrad (Figure 1A). The π–π stacking of two or more G-tetrads results in the formation of a G-quadruplex structure, which is further stabilized by metal cation coordination (e.g., K^+^ and Na^+^) [5]. G-quadruplexes can be formed by one (unimolecular), two (bimolecular) or four (tetramolecular) separated strands of DNA or RNA (Figure 1B) and can display a wide variety of topologies [1,3,5,6,7], i.e., parallel, antiparallel or hybrid, depending on the orientation of the strands (Figure 1C). Moreover, various types of loops connecting the tracts of adjacent guanines are possible, i.e., propeller, lateral or diagonal (Figure 1D) [1,3,5,7]. Particularly, antiparallel G-quadruplexes can be classified as basket-type, where two loops are lateral and one diagonal, or chair-type, where all three loops are lateral. In turn, hybrid G-quadruplexes, also indicated as (3 + 1) G-quadruplexes, since they include three parallel strands and one antiparallel, can be further classified as hybrid-1, in which the antiparallel strand is the third one starting from the 5′-end, or hybrid-2, in which the antiparallel strand is the second one [1,3,5,7]. Furthermore, while nucleobases in B-DNA are only in *anti*-conformation, in G-quadruplex structures, guanines can adopt either *anti*- or *syn*-conformation (Figure 1E). Thus, differently from B-DNA, in which there are only two different grooves, a major and a minor one, the remarkable variations in the glycosidic torsion angles result in the formation of four grooves of very different sizes (wide, medium or narrow) in the G-quadruplex backbone [1,3,5,7].

Altogether, these diverse structural features give rise to a large variety of different G-quadruplexes [5,8], resulting in a wide set of noncanonical nucleic acid structures that can be specifically targeted.

In almost all known genomes, the highest density of G-rich sequences is found at the telomeres, i.e., the ends of linear chromosomes composed of a double-stranded region and a single-stranded G-rich 3′-overhang, essential to protect DNA from degradation and end-to-end fusion [10]. Telomeric ends are elongated by telomerase, a ribonucleoprotein complex composed of a reverse transcriptase and an RNA subunit that provides the short template sequence for the telomeric DNA repeats. Telomerase activity is finely regulated in normal cells, so that, after a defined number of cell cycles, telomeres progressively shorten until they reach a critical length at which cells enter in replicative senescence [10]. On the other hand, cancer cells have evolved specific mechanisms able to maintain telomere length, mainly by telomerase overexpression, resulting in cellular immortalization, one of the main hallmarks of cancer [10,11].

The peculiar ability of telomeres to fold into G-quadruplex structures proved to have regulatory roles in telomere extension and maintenance. In detail, the formation of G-quadruplex structures makes the G-rich single-stranded overhang inaccessible to telomerase, thus inhibiting telomere extension [1]. 

Furthermore, since the human genome sequence has been fully mapped, several computational analyses have been performed to find putative G-quadruplex-forming sequences in the genome. Notably, over 700,000 putative G-quadruplex-forming sequences were found [12]. These sequences are non-randomly distributed: indeed, in addition to telomeric regions, they are mainly located within other cancer-related genomic regions, i.e., in the promoters of oncogenes, where G-quadruplexes act as modulators of the transcription process [12,13,14].

In addition, G-quadruplex structures have been also found in regulatory regions of viral genomes—such as Human Immunodeficiency Virus (HIV) and Severe Acute Respiratory Syndrome Coronavirus 2 (SARS-CoV-2)—being involved in key viral pathways, and are now emerging as targets for novel antiviral therapies [15,16,17].

Thus, targeting G-quadruplexes by small-molecule ligands can provide an appealing opportunity to finely modulate/inhibit cancer- and/or virus-related pathways [18]. 

To date, more than 3000 G-quadruplex ligands have been described [19,20,21,22,23], with most of them exerting their anticancer or antiviral activity by inducing telomeric damage and/or altering oncogene or viral gene expression in cancer cells and viruses, respectively [4,24,25,26]. Additionally, for some of the investigated G-quadruplex small-molecule ligands, in-depth structural characterizations of their complexes with the proper G-quadruplex target have been reported.

Here, the NMR and crystal structures available in the literature of the complexes between telomeric, oncogenic or viral G-quadruplexes and small-molecule ligands, of both organic and metal-organic nature, have been collected. Thus, an overview of the most relevant structural details of these complexes is here presented to provide a useful guide for the design of novel ligands targeting more efficiently and selectively cancer- and virus-related G-quadruplex structures.

## 2. Small-Molecule Ligands Targeting Telomeric, Oncogenic and Viral G-Quadruplexes

The majority of G-quadruplex small-molecule ligands share common structural features, i.e., (i) a planar (hetero)aromatic core, which can stack onto the outer G-tetrads of the G-quadruplex, and (ii) pendant groups typically containing H-bond donors/acceptors and/or terminating with positively charged moieties able to interact with the backbone negatively charged phosphates [27]. Proper combinations of the core properties and the length and nature of the pendant groups in the selected ligand allow modulating its affinity and selectivity towards the specific G-quadruplex target. 

Here, the G-quadruplex/small-molecule ligand structures obtained by NMR and X-ray crystallography have been grouped based on the G-quadruplex target, going from telomeric DNA and RNA G-quadruplexes to DNA oncogenic G-quadruplexes, and finally to RNA viral G-quadruplexes. The chemical structures of the ligands and a pictorial illustration of all the G-quadruplex/small-molecule ligand structures herein described can be found in Table S1. For comprehensive and authoritative reviews on NMR and crystallographic methods used to solve the structures of G-quadruplex/small-molecule ligand complexes, the reader can refer to refs. [28,29,30].

### 2.1. Telomeric G-Quadruplexes

Since human telomeric DNA (h-tel) and RNA (hr-tel), as well as telomeres from different species such as Oxytricha (o-tel), consist of multiple repetitions of G-rich sequences, many different G-quadruplex-forming truncations of these sequences have been investigated in various structural studies. Accordingly, the human and Oxytricha telomeric G-quadruplex targets are here divided on the basis of the molecularity and topology of their secondary structure.

#### 2.1.1. Human Telomeric Unimolecular Antiparallel G-Quadruplexes

**Figure 2:**
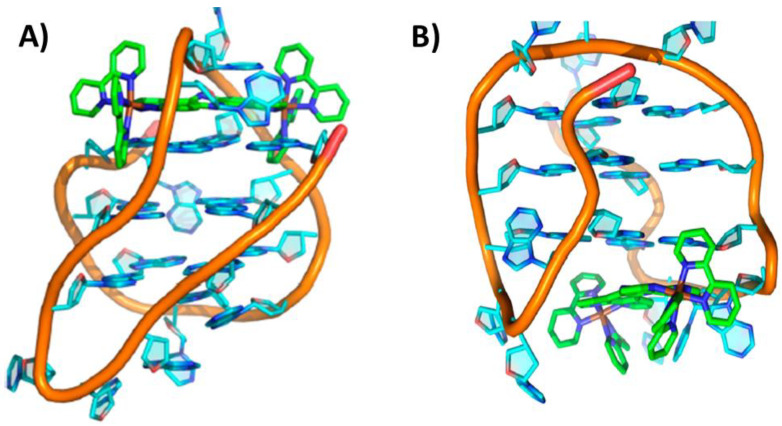
NMR structure of the complex between the unimolecular antiparallel basket G-quadruplex h-tel22 of sequence d[AGGG(TTAGGG)_3_] and (**A**) ΛΛ-[{Ru(phen)_2_}_2_tpphz]^4+^ or (**B**) ΔΔ-[{Ru(phen)_2_}_2_tpphz]^4+^. 5′- and 3′-end of the G-quadruplex are at the top and bottom, respectively. Adapted with permission from ref. [31], Copyright 2013 American Chemical Society.

The interaction of the two enantiomers, ΛΛ and ΔΔ, of the dinuclear ruthenium complex [{Ru(phen)_2_}_2_tpphz]^4+^ (phen = 1,10-phenanthroline and tpphz = tetrapyrido[3,2-*a*:2′,3′-*c*:3″,2″-*h*:2‴,3‴-*j*]phenazine) with the unimolecular antiparallel basket G-quadruplex h-tel22 of sequence d[AGGG(TTAGGG)_3_] was studied by NMR [31]. The main binding site of the ΛΛ enantiomer was the diagonal loop of the G-quadruplex (PDB ID: 2MCO). More in detail, this enantiomer was optimally inserted between the diagonal loop and the adjacent 5′-end G-tetrad, and the binding was reinforced by both stacking onto the G-tetrad and electrostatic interactions between the ruthenium cations and the oligonucleotide phosphate anions (Figure 2A). On the other hand, the ΔΔ enantiomer, unable to bind underneath the diagonal loop, stacked at the 3′-end of the G-quadruplex (Figure 2B), particularly in proximity of one of the two lateral loops (PDB ID: 2MCC). Notably, the binding affinity of the ΛΛ enantiomer was ~40-fold higher than the ΔΔ enantiomer [31]. In addition, when the ΛΛ enantiomer bound to the h-tel22 G-quadruplex, its luminescence was more intense compared to the ΔΔ enantiomer and also shifted from ~675 to ~630 nm [31]. Moreover, the ΛΛ enantiomer luminescence was higher when bound to G-quadruplexes than to B-DNA models, according to its higher affinity for the G-quadruplex than duplex DNA. More notably, the ΛΛ enantiomer also exhibited a wavelength and intensity of emission different when interacted with different G-quadruplex structures [32]. Thus, the ΛΛ enantiomer of [{Ru(phen)_2_}_2_tpphz]^4+^ emerged as a promising tool for specifically stabilizing and imaging antiparallel basket G-quadruplexes [31].

Additionally, the ruthenium complex Λ-[Ru(phen)_2_(qdppz)]^2+^ (qdppz = 12,17-dihydronaphtho[2,3-h]dipyrido[3,2-a:2′,3′-c]phenazine-12,17-dione), able to strongly inhibit DNA replication, was studied in its interaction with the unimolecular antiparallel chair G-quadruplex h-tel21 of sequence d[GGG(TTAGGG)_2_TTTGGG] by X-ray crystallography (PDB ID: 7OTB) [33]. A 1:1 G-quadruplex/ligand-binding stoichiometry was found, with the ligand positioned between the 5′-end G-tetrad and central lateral loop T10-T11-A12, thus forming specific stacking interactions [33].

#### 2.1.2. Human Telomeric Unimolecular Hybrid G-Quadruplexes

Berberine and its derivatives have been shown to stabilize telomeric G-quadruplexes and inhibit telomerase activity [34,35]. Particularly, the natural product epiberberine (EPI) exhibited a strong fluorescence enhancement upon binding to human telomeric hybrid G-quadruplexes, not observed if bound to parallel and antiparallel G-quadruplexes or duplex DNA [36]. In order to find the rationale behind this notable behavior, a NMR study was performed to investigate the interaction of EPI with the unimolecular hybrid-2 G-quadruplex h-tel26 of sequence d[(TTAGGG)_4_TT] (PDB ID: 6CCW) [37]. The NMR analysis showed that the disordered 5′-flanking segment TTA and the second lateral loop of the G-quadruplex rearranged completely upon binding to EPI. The ligand formed a pseudo-layer with the flanking A3, and intercalated between the 5′-end G-tetrad and two planes, i.e., a T2:T13:A15 triad layer and a T1:T14 pair, stabilized by hydrogen bond interactions. More in detail, EPI interacted by hydrogen bonds with the flanking A3 and by stacking interactions with the 5′-end G-tetrad [37]. Interestingly, starting: (i) from G-quadruplex-forming sequences folded in a mixture of hybrid-1 and hybrid-2 topologies or (ii) from sequences forming a predominant hybrid-1 or parallel topology or (iii) even from an unfolded human telomeric DNA sequence in the absence of salt, EPI was able to shift the conformational equilibrium to promote the formation of the hybrid-2 topology G-quadruplex, which exposes its specific binding pocket between the 5′-end G-tetrad and the system TTA triad layer/TT pair. The importance of the flanking A3 and loop A15 residues in the stability of the complex was further proved by modification of these residues, which completely disrupted the complex formation. The intercalation of EPI in its specific binding pocket can thus explain the significant fluorescence enhancement of EPI induced by binding to the telomeric hybrid-2 G-quadruplex [37].

The unimolecular G-quadruplex h-tel26 was also exploited to study the interaction with the dinuclear Au(III) complex Auoxo6 by NMR (PDB ID: 5MVB) [38]. As reported above, the G-quadruplex h-tel26 of sequence d[(TTAGGG)_4_TT] mainly folds in a hybrid-2 topology in K^+^-containing solutions [37]. However, minor species are also present in equilibrium with the major fold. When the ligand interacted with h-tel26 G-quadruplex, the equilibrium was shifted towards the hybrid-2 topology and, in detail, from 70% of the free G-quadruplex to 90% in the case of the bound G-quadruplex. Interestingly, the binding involved a conformational rearrangement of the 5′-capping moiety of the G-quadruplex, and the ligand was sandwiched between the 5′-end G-tetrad and the flanking A3 [38].

Structural models of the interactions between the tripodal cationic ligand NBTE and the unimolecular hybrid-2 G-quadruplex h-tel26 or the unimolecular hybrid-1 G-quadruplex h-tel26A (of sequence d[AAAGGG(TTAGGG)_3_AA], obtained from h-tel26 sequence by T-to-A mutations for T1, T2, T25 and T26), were obtained by NMR (PDB IDs: 6KFJ and 6KFI, respectively) [39]. This ligand presented three arms: only two of them bound the model duplex DNA while all of them interacted with the G-quadruplex structures. These different binding modes resulted in significantly different fluorescence lifetime responses and quantum yield enhancement, which allowed using NBTE as a suitable fluorescent probe to detect G-quadruplexes in live cells, proving that the G-quadruplex content was 4-fold higher in cancer than normal cells [39]. In both models, the ligand bound the G-quadruplexes at the 5′-end G-tetrad by stacking interactions. Moreover, the binding of the ligand determined a significant rearrangement of the 5′-end residues, thus inducing the formation of a capping triad—composed of A3, T14 and A21 for hybrid-2, and of A3, A9 and T20 for hybrid-1—on top of the ligand stacked onto the G-tetrad. Further stabilization to the binding was provided by π-cation and electrostatic interactions at the 5′-end by the positively charged ethylpyridinium groups [39].

**Figure 3:**
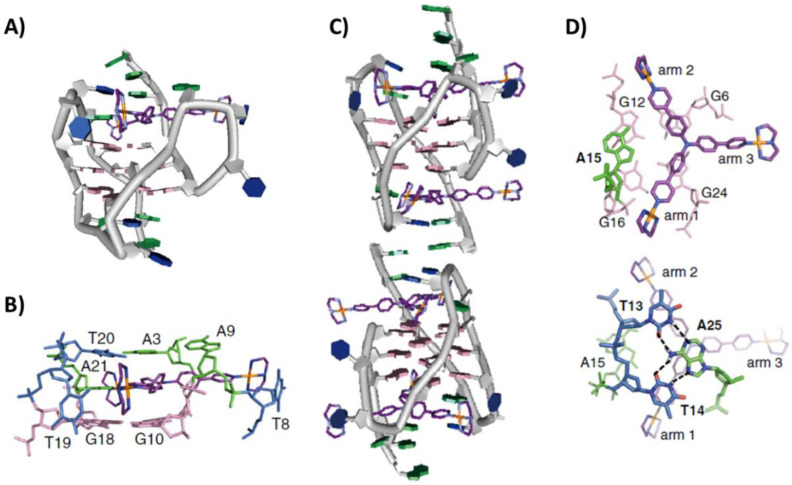
NMR structure of the complex between the unimolecular hybrid-1 h-tel26 G-quadruplex of sequence d[AAAGGG(TTAGGG)_3_AA] and Pt(II)-based tripod at (**A**) 1:1 or (**C**) 2:4 G-quadruplex/ligand stoichiometry. In (**A**), the 5′- and 3′-end of the G-quadruplex are at the top and bottom, respectively, while, in (**C**), G-quadruplex units are 3′-3′ stacked. Details of the ligand binding are highlighted in (**B**) for 1:1 and (**D**) for 2:4 G-quadruplex/ligand complexes. Adapted with permission from ref. [40], Copyright 2018 The Author(s).

Another NMR study provided information on the binding of a Pt(II)-based tripod, an inhibitor of telomerase activity, with the unimolecular hybrid-1 G-quadruplex h-tel26A (PDB IDs: 5Z80 and 5Z8F related to 1:1 and 2:4 G-quadruplex/ligand stoichiometry, respectively) [40]. The preferential binding site of the Pt-tripod, at low Pt-tripod/h-tel26 ratios (from 0 to 1), was at the 5′-end, where it recruited A21 to form an A21-Pt-tripod plane, stacking on top of the 5′-end G-tetrad. The A21-Pt-tripod plane was further covered and stabilized by the ligand-induced triad formed by A3, A9 and T20. In addition, loop residues T8 and T19 were also rearranged to interact with the NH of two platinum units through hydrogen bonding (Figure 3A,B). On the other hand, at higher Pt-tripod/h-tel26A ratios, the second Pt-tripod molecule bound the 3′-end, thus inducing the formation of a dimeric G-quadruplex structure interlocked by an A:A noncanonical pair at the 3′-3′ interface. A15 at the 3′-end was recruited by the second Pt-tripod to form an A15-Pt-tripod plane, which was further covered by a hydrogen-bonded T13:A25:T14 triad (Figure 3C,D) [40]. More in detail, the unique binding mode of Pt-tripod included first utilizing its two arms to recruit an adenine to form an A-Pt-tripod plane covering the external G-tetrad, with the two platinum cations interacting with loop residues and negatively charged phosphates of two grooves, and then exploiting the third arm to further lock the Pt-tripod binding position by similar interactions with the third groove and stabilization of the propeller loop. Overall, the binding comprised a combination of multiple interaction modes including stacking, hydrogen bonding and electrostatic interactions [40].

NMR studies allowed also to solve the structure of the complex between the telomestatin derivative L2H2-6M(2)OTD, exhibiting high selectivity toward G-quadruplex structures and potent telomerase inhibitory activity, and the unimolecular hybrid-1 G-quadruplex h-tel24 of sequence d[TTGGG(TTAGGG)_3_A] (PDB ID: 2MB3) [41]. The preferential binding site of the ligand was the 5′-end G-tetrad, onto which the oxazole rings could properly stack. Moreover, the two cationic side chains of the ligand were directed toward the negatively charged phosphates of h-tel24, thus producing electrostatic interactions. More notably, a potassium cation was trapped between the ligand and the 5′-end G-tetrad, further stabilizing the structure. Interestingly, the free ligand was featured by a nonplanar “roof-like” bent conformation, which, upon binding, became more planar to maximize the stacking interaction with the G-tetrad [41].

The complex between the unimolecular hybrid-1 G-quadruplex h-tel23 of sequence d[TAGGG(TTAGGG)_3_] and the bisquinolinium compound Phen-DC3 was recently solved by NMR (PDB ID: 7Z9L) [42]. Interestingly, the ligand was able to change the G-quadruplex fold from hybrid-1 to antiparallel chair. More notably, the ligand intercalated between a two-tetrad unit and a more dynamic 5′-end G-tetrad, referred to as a “pseudo-tetrad” since formed by the four guanines G3, G11, G15 and G23, wherein for G3–G11 and G11–G15 pairs H1–H8 NOE correlations were not observed. More in detail, the quinolinium units of Phen-DC3 were intercalated between G10–G11 and G15–G16 respectively, while the phenanthroline ring was located between G4–G22 and G3–G23 [42].

#### 2.1.3. Human Telomeric Unimolecular Parallel G-Quadruplexes

The natural alkaloid berberine was studied in its interaction with the unimolecular parallel G-quadruplex h-tel23 of sequence d[TAGGG(TTAGGG)_3_] by X-ray crystallography (PDB ID: 3R6R) [43]. Interestingly, two G-quadruplexes h-tel23 were found to dimerize forming a 5′-5′ binding pocket and give a complex with 1:3 G-quadruplex/ligand stoichiometry. Indeed, two couples of berberine molecules were stacked onto each of the two 3′-end G-tetrads of the dimer and interacted by hydrogen bonds with a water molecule positioned in correspondence of the G-quadruplex central channel. Moreover, two additional berberine molecules were sandwiched between the 5′-end G-tetrads, interacting with them by stacking interactions. At both 3′- and 5′-ends, the berberine molecules interacted with each other by Van der Waals interactions, thus forming coplanar couples that allowed maximizing the stacking interactions [43].

**Figure 4:**
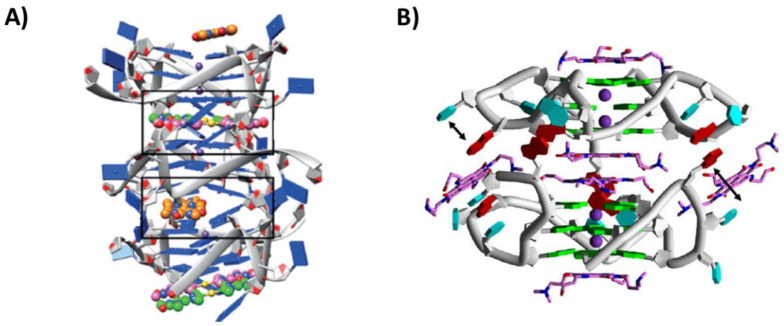
(**A**) Crystal structure of the complex between the unimolecular parallel G-quadruplex h-tel23 of sequence d[TAGGG(TTAGGG)_3_] and [Au(9-methylcaffein-8-ylidene)_2_]^+^ [44]. (**B**) Crystal structure of the complex between the unimolecular parallel G-quadruplex h-tel23 of sequence d[TAGGG(TTAGGG)_3_] and NDI-1 [47]. G-quadruplex units are 5′-5′ stacked. Adapted with permission from refs. [44,47], Copyright 2016 WILEY-VCH Verlag GmbH & Co. KGaA, Weinheim and Copyright 2008 Elsevier Ltd.

In a different study, the structure of the complex of the Au(I) bis-carbene [Au(9-methylcaffein-8-ylidene)_2_]^+^ with the unimolecular parallel G-quadruplex h-tel23 was solved using X-ray crystallography (PDB ID: 5CCW) [44]. Three ligand molecules bound the G-quadruplex at two distinct sites, i.e., the 3′-end G-tetrad, accommodating one ligand molecule, and the 5′-end G-tetrad, wherein the other two molecules were stacked (Figure 4A). In detail, only one caffeine unit per ligand molecule interacted with the guanine residues, while the second caffeine unit protruded outside the G-tetrad surface [44]. Subsequently, another Au(I) bis-carbene complex, i.e., [Au(1-butyl-3-methyl-2-ylidene)_2_]^+^, forming a 1:1 adduct with the unimolecular parallel G-quadruplex h-tel24 of sequence d[TAGGG(TTAGGG)_3_T], was investigated (PDB ID: 6H5R) [45]. In this case, the bis-carbene ligand was stacked onto two guanines at the 5′-end G-tetrad [45].

Another ligand studied in its interaction with the unimolecular parallel G-quadruplex h-tel23 was a tetrasubstituted naphthalene diimide (NDI) derivative, bearing two dimethylaminopropyl and two hydroxypropylamino groups (NDI-1). This ligand proved to better stabilize telomeric G-quadruplexes over duplex DNA, also showing cancer cell senescence induction, as well as high antiproliferative activity (IC_50_ value of 287.7 nM on MCF-7 cancer cell line) [46]. Considering its interesting properties, it was in-depth investigated with the G-quadruplex h-tel23. The crystal structure of this complex was solved (PDB ID: 3CDM) [47], showing a binding stoichiometry of 1:6 G-quadruplex/ligand (Figure 4B). Indeed, two G-quadruplexes h-tel23 were 5′-5′ stacked and two ligand molecules were stacked onto each of the two 3′-end G-tetrads, two additional molecules were stacked on each other and inserted into the 5′-5′ binding pocket formed by the two G-quadruplexes, while other two ligand molecules were stacked between an adenine and a thymine of the loops, which were swung out from their orientation in the native structure [47].

Two additional different tetrasubstituted NDI derivatives with propyl (BMSG-SH3) or butyl (BMSG-SH4) side chains terminating with *N*-methyl-piperazine moieties were studied in their complexes with the unimolecular parallel G-quadruplex h-tel22 of sequence d[AGGG(TTAGGG)_3_] by X-ray crystallography (PDB IDs: 3SC8 and 3T5E, respectively) [48]. In both cases, 1:1 G-quadruplex/ligand complexes were formed, thus indicating a specificity in the binding mode of these NDIs to telomeric G-quadruplex DNA. Particularly, two individual G-quadruplexes formed a dimer stabilized by stacking of their 5′-end G-tetrads, and two NDI ligands stacked each onto one of the two 3′-end G-tetrads of the dimer. In detail, BMSG-SH3 and BMSG-SH4 were asymmetrically or symmetrically positioned over the 3′-end G-tetrads, respectively. As far as the NDI pendant groups are concerned, only two of the four side chain groups in BMSG-SH3 were deeply positioned within the G-quadruplex grooves, having close direct contact between their positively charged methylpiperazine ring nitrogen atoms and the negatively charged phosphates of the G-quadruplex grooves. On the other hand, all four side chains of BMSG-SH4 were positioned in the G-quadruplex grooves as a consequence of the central position of its NDI core on the G-tetrad. However, the contacts between BMSG-SH4 and groove atoms were weaker and less specific than BMSG-SH3. This peculiar behavior well-explained the higher ability to selectively stabilize G-quadruplex over other DNA secondary structures, as well as the more selective cancer vs. normal cells activity of BMSG-SH3 than BMSG-SH4 [48].

Based on the promising data obtained for BMSG-SH3 [48], several BMSG-SH3-based NDI derivatives were designed and evaluated for their interaction with G-quadruplex structures by X-ray crystallography [49]. Aiming at improving the pharmacological properties of BMSG-SH3 by decreasing its basic properties but preserving its overall size and binding to G-quadruplexes, two of the four *N*-methyl-piperazine moieties were substituted by two morpholine groups, thus obtaining the derivative named 3d. The crystal structure of 3d with the unimolecular parallel G-quadruplex h-tel22 was solved (PDB ID: 3UYH), proving that the morpholine groups did not modify the main interactions between the ligand and the G-quadruplex already observed for BMSG-SH3. Additionally, the crystal structures of 3d and BMSG-SH3 in complex with the unimolecular parallel G-quadruplex h-tel21 of sequence d[GGG(TTAGGG)_3_] were also obtained (PDB IDs: 4DA3 and 4DAQ, respectively), showing no relevant difference in their ligand binding behavior compared to that observed with the unimolecular parallel G-quadruplex h-tel22. Finally, 3d showed 10-fold higher activity on different cancer cell lines than BMSG-SH3, with IC_50_ values in the range 10–20 nm [49].

In the crystal structure between the unimolecular parallel G-quadruplex h-tel22 and *N*-methyl mesoporphyrin IX (NMM) (PDB IDs: 4FXM and 4G0F, related to two different crystal forms where the former had higher resolution) [50], the ligand showed not only a high G-quadruplex over duplex DNA selectivity but also the ability to specifically recognize parallel vs. antiparallel G-quadruplex topologies [51,52]. Two G-quadruplexes h-tel22 formed a dimer stabilized by 5′-5′ stacking interactions, while NMM exhibited an optimized stacking interaction with the 3′-end G-tetrads by adjusting its macrocycle geometry to maximize the stacking interactions. Particularly, the *N*-methyl group of NMM fitted perfectly into the center of the parallel G-quadruplex aligning the potassium cations. Notably, the *N*-methyl group was responsible for the high specificity for parallel G-quadruplexes. Indeed, neither antiparallel G-quadruplexes nor duplex structures had sufficient space to accommodate the NMM *N*-methyl group, leading to steric clashes that prevented the ligand binding [50].

Finally, the Pt(II)-based compound bearing two 3-(pyridin-2-yl)-[1,2,4]triazole[4,3-a]pyridine ligands, which demonstrated very strong stabilization and affinity of/for G-quadruplexes and good selectivity over duplex DNA, was studied in its complex with the unimolecular parallel G-quadruplex h-tel22 by X-ray crystallography (PDB ID: 6XCL) [53]. In this crystal structure, two G-quadruplexes formed a dimer interacting through 5′-5′ G-tetrads stacking. The ligand could interact with both 3′- and 5′-end G-tetrads of the G-quadruplex: one ligand molecule was stacked within the two facing 5′-5′ G-tetrads, while the two 3′-end G-tetrads exposed to the solvent were capped with one ligand each [53].

#### 2.1.4. Human Telomeric Bimolecular Parallel G-Quadruplexes

**Figure 5:**
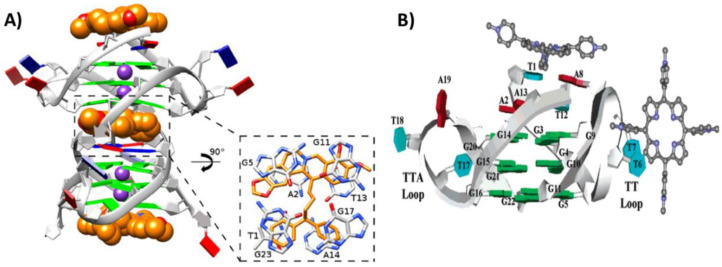
(**A**) Crystal structure of the complex between the bimolecular parallel G-quadruplex h-tel12 of sequence d(TAGGGTTAGGGT) and NAX053 [54]. (**B**) Crystal structure of the complex between the bimolecular parallel G-quadruplex h-tel11 of sequence d(TAGGGTTAGGG) and TMPyP4 [59]. In (**A**) the G-quadruplex, units are 5′-5′ stacked, while, in (**B**) the 5′- and 3′-ends of the G-quadruplex, they are at the top and bottom, respectively. Adapted with permission from refs. [54,59], Copyright 2016 WILEY-VCH Verlag GmbH & Co. KGaA, Weinheim and Copyright 2007 American Chemical Society.

In addition to the above characterization of a G-quadruplex/berberine complex [43], more detailed studies on berberine derivatives, differently substituted in position 13, with various G-quadruplexes were performed by X-ray crystallography [54,55]. Particularly, aiming at increasing the overall stacking interactions, berberine was derivatized with a phenylalkyl (NAX039 and NAX042) or a benzhydrylalkyl group (NAX035 and NAX053) [54]. In the case of NAX053, the structure of its complex with the bimolecular parallel G-quadruplex h-tel12 of sequence d(TAGGGTTAGGGT) was obtained (PDB ID: 5CDB). NAX053 was stacked between the 3′-end G-tetrad and the 5′-end T:A:T:A platform of two different G-quadruplex units, forming complexes with an overall 2:3 G-quadruplex/ligand stoichiometry (Figure 5A). The berberine ligand core was similarly placed onto the 3′-end G-tetrad as in the h-tel23/berberine complex, whereas the 13-benzhydrylalkyl groups replaced one of the two berberine molecules in the ligand plane, with the two phenyl groups in a nonplanar arrangement and the alkyl chain directed toward the G-tetrad [54]. Interestingly, NAX053 proved to be more cytotoxic on several cancer cell lines than berberine (IC_50_ values of 2.56 and 2.27 µM for HeLa and MCF-7 cancer cell lines, respectively, compared to 18.82 and 11.75 µM). Similar interactions were found for another 13-substituted berberine derivative in which an alkylpyridine was present as the side chain, thus suggesting a conservative binding mode for berberine derivatives mainly driven by the berberine core (PDB ID: 6S15) [55].

The tetrasubstituted naphthalene diimide derivative NDI-1 was also studied in its interaction with the parallel bimolecular G-quadruplex h-tel12 by X-ray crystallography (PDB ID: 3CCO) [47]. A different binding stoichiometry was found compared to its complex with the unimolecular parallel G-quadruplex h-tel23, i.e., 1:3 G-quadruplex/ligand. Indeed, in this case, a ligand molecule stacked on the 3′-end G-tetrad, whereas the additional two ligand molecules were stacked on each other and interacted with T7 in the loop by stacking interactions [47].

The 3,6,9-trisubstituted acridine derivative BRACO-19 showed marked anticancer activity both in vitro and in vivo associated with telomere uncapping [56,57]. The structure of its complex with the bimolecular parallel G-quadruplex h-tel12 was solved by X-ray crystallography (PDB ID: 3CE5) [58]. BRACO-19 was asymmetrically positioned on the 3′-end G-tetrad, thus forming stacking interactions with two of the four guanines of the G-tetrad. The other side of the acridine core surface was stacked onto a reverse Watson-Crick A:T base pair of a 5′-end T:A:T:A tetrad of a second G-quadruplex unit in the lattice. The 3- and 6-position substituents of BRACO19 were located on opposite sides of the G-tetrad plane, while the 9-position of the anilino substituent fitted into a narrow pocket at the dimer interface. Among the eight donor-acceptor substituents in BRACO-19, seven participated in H-bonding, six of which were bound to water molecules rather than directly to the G-quadruplex. Thus, the crystal structure explained the key role of the 9-position anilino substituent, which increased its affinity for G-quadruplexes by 10-fold [58].

Another crystallographic study was devoted to investigating the interactions of the cationic porphyrin TMPyP4 with the bimolecular parallel G-quadruplex h-tel11 of sequence d(TAGGGTTAGGG) (PDB ID: 2HRI) [59]. Notably, TMPyP4 bound to two different binding sites, but none of them included the G-tetrads. Indeed, the ligand molecules interacted with the TTA loops in two different ways. Specifically, one TMPyP4 was stacked onto the A:T base pair formed by the 5′-end T1 and the A8 from the TTA loop, which rearranged thus forming the T6-T7 loop, while the second ligand molecule was stacked on the T6-T7 loop (Figure 5B). The latter molecule was stabilized in the lattice by further interactions with the T17-T18-A19 loop of a second G-quadruplex unit [59].

Finally, two crystal structures of Cu(II) and Ni(II) salphen metal complexes with the bimolecular parallel G-quadruplex h-tel11Br, modified with a 5-bromo-2′-deoxyuridine-5′-monophosphate monomer, of sequence d(AGGGTBrUAGGTT) were solved (PDB IDs: 3QSC and 3QSF relative to the Cu(II) and Ni(II) complexes, respectively) [60]. In both cases, two G-quadruplexes interacted by stacking interactions at their 5′-end, whereas the ligand was bound at the 3′-end and, in particular, sandwiched between the 3′-end G-tetrad and two water-bridged 3′-end thymines of another G-quadruplex unit of the lattice. The observed binding poses resulted in positioning the metals in line with the central channel of the G-quadruplex. Notably, the Ni(II) complex bound more strongly and was more stabilizing for G-quadruplex structures than the Cu(II) complex. This was explained considering that the bending of the Ni(II) complex was lower than that produced by the Cu(II) complex, thus allowing a higher degree of stacking of the phenyl rings onto the G-tetrad [60].

#### 2.1.5. Human Telomeric Tetramolecular Parallel G-Quadruplexes

A racemic mixture of Λ/Δ-[Ru(TAP)_2_(11-CN-dppz)]^2+^ (TAP = 1,4,5,8-tetraazaphenanthrene; DPPZ = dipyridophenazine) in complex with the tetramolecular parallel G-quadruplex h-tel8 of sequence d(TAGGGTTA) was evaluated and its structure solved by X-ray crystallography (PDB ID: 5LS8) [61]. Notably, the structural analysis revealed a complete conversion of the G-quadruplex topology from parallel to antiparallel only for the Λ-enantiomer. Direct stacking interactions of Λ-enantiomer with both the outer G-tetrads were found. Particularly, two Λ-enantiomer molecules per side of the G-quadruplex were intercalated between the outer G-tetrads and the external T/A tetrads, giving an overall stoichiometry of four molecules for a single G-quadruplex. On the other hand, two Δ-enantiomer molecules were stacked on the terminal T–T pairs, also keeping together two adjacent G-quadruplex units in the crystal lattice, with no direct interaction with the G-tetrads. The unusual antiparallel topology observed for h-tel8 was probably due to the ability of the Λ-enantiomer, unlike the Δ-enantiomer, to strongly stabilize the *syn*-conformation of the deoxyguanosines [61].

**Figure 6:**
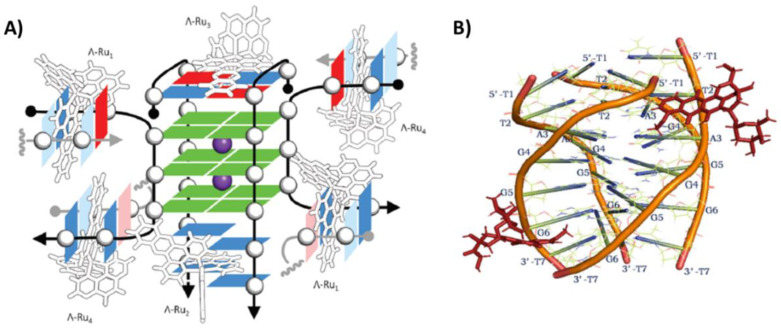
(**A**) Crystal structure of the complex between the parallel tetramolecular G-quadruplex h-tel7 of sequence d(TAGGGTT) and Λ-[Ru(TAP)_2_(dppz)]^2+^ [62]. (**B**) NMR structure of the complex between the parallel tetramolecular G-quadruplex h-tel7 of sequence d(TTAGGGT) and adriamycin [64]. 5′- and 3′-ends of the G-quadruplex are at the top and bottom, respectively. Adapted with permission from refs. [62,64], Copyright 2019 The Royal Society of Chemistry and Copyright 2021 Taylor & Francis.

Additionally, the Λ-enantiomer of [Ru(TAP)_2_(dppz)]^2+^ was studied in its interaction with the tetramolecular parallel G-quadruplex h-tel7 of sequence d(TAGGGTT) by X-ray crystallography (PDB ID: 6RNL) [62]. Differently from the complex formed with h-tel8, the G-quadruplex h-tel7 maintained its usual parallel topology. Moreover, the four interacting molecules of the Λ-enantiomer were bound to four distinct binding sites [62]. Indeed, the terminals T1-A2 and T6-T7 were kinked out of the G-quadruplex core, thus accommodating two Λ-enantiomer molecules and producing an overall bent structure. Finally, the third and fourth molecules were positioned directing the TAP or dppz group, respectively, towards a T–T linkage or a T–A–T–A tetrad, thus forming stacking interactions (Figure 6A) [62].

On the other hand, two NMR structures between the parallel tetramolecular G-quadruplex h-tel7 of sequence d(TTAGGGT) and two anthracycline molecules, i.e., epirubicin (PDB ID: 6KXZ) [63] and adriamycin (also known as doxorubicin) (PDB ID: 6KN4) [64], were recently solved. In the case of epirubicin, 1:2 G-quadruplex/ligand complexes were observed with epirubicin interacting in two different binding sites in the grooves, i.e., at T1-T2-A3 and G6-T7. The ring D of both epirubicin molecules interacted by hydrogen bonds with the G-quadruplex groove, while the rest of the molecule did not participate in the interaction [63]. Adriamycin showed a similar behavior, although with slight differences; indeed, while the binding to T1-T2-A3 was similar, in the other binding site, the D ring of adriamycin displaced T7 in order to stack on G6 (Figure 6B) [64].

In another study, the flavonoid quercetin, showing anticancer and antioxidant activities [65,66], was found to strongly bind to the parallel tetramolecular G-quadruplex h-tel7, and the structure of this complex was solved by NMR (PDB ID: 2MS6) [67]. The natural compound interacted with the G-quadruplex structure by stacking between the T1 and T2 tetrads, as well as between the 3′-end G-tetrad and the T7 tetrad [67].

Additionally, the quinacridone-based ligand MMQ1 was studied in its interaction with the parallel tetramolecular G-quadruplex h-tel7 by NMR (PDB ID: 2JWQ). It showed stacking interactions with both the 5′- and 3′-end G-tetrads, and the cationic side chains were directed toward the negatively charged G-quadruplex grooves [68].

Additionally, the structure of the fluorinated acridine RHPS4, known as a potent inhibitor of telomerase activity, in a complex with the parallel tetramolecular G-quadruplex h-tel7 was solved by NMR (PDB ID: 1NZM) [69]. A 1:2 G-quadruplex/ligand stoichiometry was observed with the two RHPS4 molecules stacked onto both the outer G-tetrads. Interestingly, at the 5′-end, the acridine moiety intercalated between the G-tetrad and the A-tetrad, which further stabilized the overall structure. Moreover, the partial positive charge on position 13-N of the acridine ring acted as a “pseudo”-potassium cation being positioned in line with the central channel of the G-quadruplex [69].

#### 2.1.6. Oxytricha Telomeric Unimolecular Parallel G-Quadruplexes

The interactions between the ligand NMM and two different Oxytricha telomeric truncations, i.e., the unimolecular parallel G-quadruplexes o-tel20 and o-tel18 of sequence d[(TGGGT)_4_] and d[(GGGTT)_3_GGG], respectively, were studied by X-ray crystallography (PDB IDs: 6P45 and 6PNK, respectively) [70]. In both complexes, a 1:1 G-quadruplex/ligand stoichiometry was observed [70]. Analogously to the h-tel22/NMM complex [50], NMM interacted at both the 3′-end G-tetrads of a 5′-5′ dimer of parallel G-quadruplexes, and both molecules adopted a nonplanar conformation, thus optimizing the stacking interactions [70].

**Figure 7:**
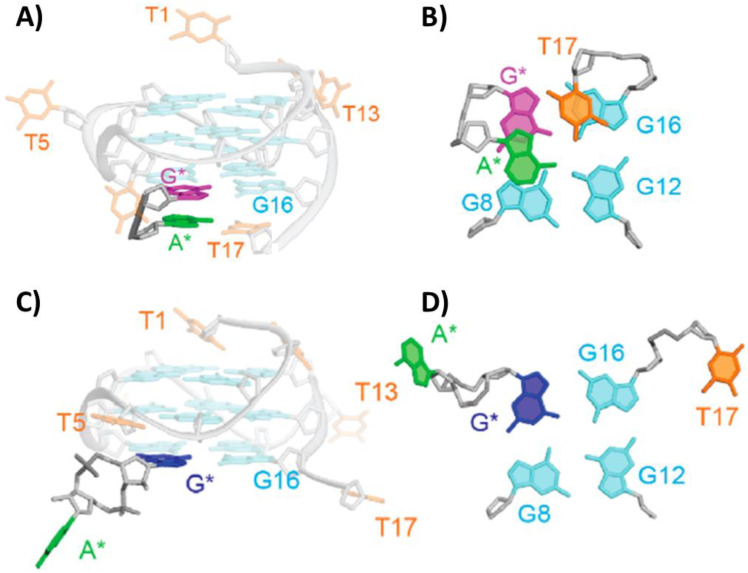
NMR structure of the complex between the unimolecular parallel G-quadruplex o-tel17 of sequence d[TTGGT(GGGT)_3_] and (**A**) d(AG) or (**C**) cGAMP. 5′- and 3′-ends of the G-quadruplex are at the top and bottom, respectively. Details of the ligand binding are highlighted in (**B**) for d(AG) and (**D**) for cGAMP. Adapted with permission from ref. [71], Copyright 2019 American Chemical Society.

Interestingly, dinucleotides were also studied as G-quadruplex ligands. In this context, two structures between the unimolecular parallel G-quadruplex o-tel17 of sequence d[TTGGT(GGGT)_3_] and two different dinucleotides, i.e., linear d(AG) and cyclic cGAMP, were solved by NMR (PDB IDs: 6K3X and 6K3Y, respectively) [71]. The guanine base of d(AG) dinucleotide interacted with the vacant G-triad, thus filling the G-vacancy in the structure and forming a complete G-tetrad, whereas the adenine interacted with the T17 flanking base via Watson Crick hydrogen bonds (Figure 7A,B). In the case of cGAMP, only guanine G-tetrad filling of the vacancy was observed, due to the lower mobility of adenine in the cyclic dinucleotide, which hampered the putative interaction with T17 (Figure 7C,D). However, even in the absence of the A:T terminal base pair, the overall complex proved to be fully stable [71].

#### 2.1.7. Oxytricha Telomeric Bimolecular Antiparallel G-Quadruplexes

**Figure 8:**
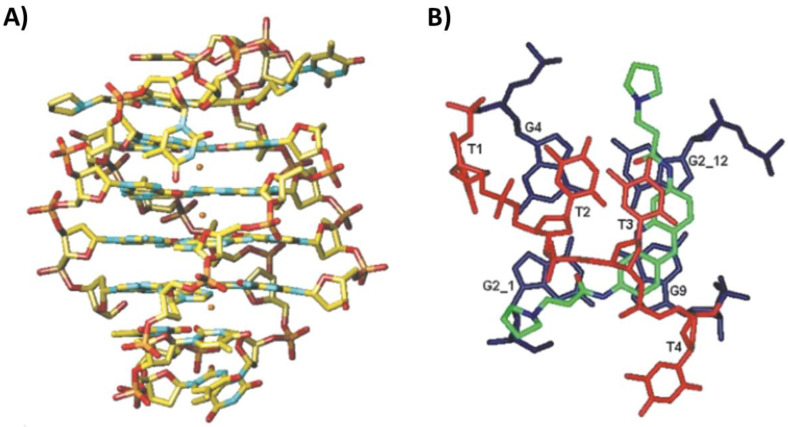
(**A**) Crystal structure of the complex between the bimolecular antiparallel G-quadruplex of o-tel12 sequence d(GGGGTTTTGGGG) and BSU6039. Details of the ligand binding are highlighted in (**B**). Adapted with permission from ref. [72], Copyright 2003 Elsevier Science Ltd.

Several 3,6-disubstituted acridine derivatives were in-depth analyzed for their interaction with the Oxytricha telomeric bimolecular G-quadruplex models. First of all, X-ray crystallography allowed insight into the structure of the complex between the acridine derivative BSU6039, with two 3-pyrrolopropionamido groups and able to inhibit telomerase activity, and the bimolecular antiparallel G-quadruplex o-tel12 of sequence d(GGGGTTTTGGGG) (PDB ID: 1L1H) [72]. The structure included a single acridine derivative molecule bound per G-quadruplex. In detail, the molecule bound the G-quadruplex within one of the two TTTT loops, with the second thymine residue of the loop positioned in the plane of the acridine core (Figure 8A). BSU6039 was sandwiched between two guanines of the 5′-end G-tetrad and T3, and the complex was stabilized by stacking interactions. The complex was further stabilized by hydrogen bonds formed by the ligand with T2 and T4 (Figure 8B). Notably, a significant modification occurred in the TTTT loop upon ligand binding: indeed, the T1 and T4 were swung out from the loop and formed T1–T4 stacking interaction with a second G-quadruplex unit in the lattice [72].

Successively, additional 3,6-disubstituted acridine derivatives were evaluated, featured by the following side chains: 3-morpholinopropionamido groups (PDB ID: 3EM2), 3-(2-ethylpiperidino)propionamido groups (PDB IDs: 3EQW and 3EUI, related to two different crystal forms), 3-(2-methylpiperidino)propionamido groups (PDB ID: 3ERU), 3-(4-methylpiperidino)propionamido groups (PDB ID: 3ES0), 3-(3-methylpiperidino)propionamido groups (PDB ID: 3ET8) and 3-(azepan-1-yl)propionamido groups (PDB ID: 3EUM). All these derivatives showed a similar binding mode towards the G-quadruplex model as BSU6039 [73].

Additional derivatives of BSU6039, differing from the parent acridine compound for the fluorine substitution at C-3 of the pyrrolidine rings, were also designed, thus obtaining the two bis-3-fluoropyrrolidine enantiomers (*R*,*R*) and (*S*,*S*). Both enantiomers were studied in their interaction with the bimolecular antiparallel G-quadruplex o-tel12 and the structures of their complexes solved by X-ray crystallography (PDB IDs: 3NYP and 3NZ7, respectively) [74]. Both fluorinated-derivative complexes showed the acridine moiety in an identical position as BSU6039, while the pyrrolidinium N^+^-H was oriented in the opposite direction compared to BSU6039. The fluoro-substituted pyrrolinidium rings interacted with the oligonucleotide backbone phosphates, whereas the nonfluorinated ones in BSU6039 formed classical hydrogen bonds with either a ribose ring or a water molecule [72]. However, the fluorinated derivatives stabilized the G-quadruplex model less than BSU6039. This was attributed to the fact that the ligand was no longer acting as an anchor to secure the top and bottom layers of the structure, hence weakening the overall integrity of the complex [74].

The last ligand studied in its interaction with the bimolecular antiparallel G-quadruplex o-tel12 was the aromatic oligoamide foldamer, consisting of a repetition of quinoline units, and the structure of this complex was solved by X-ray crystallography (PDB ID: 5HIX) [75]. Interestingly, the ligand adopted a helical arrangement stabilized by stacking interactions, whereas the cationic groups protruded toward the solvent. Particularly, the foldamer molecules were intercalated in the G-quadruplex lattice, and their cationic groups interacted with the phosphate groups in the G-quadruplex loops by electrostatic interactions, overall forming 1:1 complexes, while no stacking interactions were observed [75].

#### 2.1.8. Oxytricha Telomeric Tetramolecular Parallel G-Quadruplexes

**Figure 9:**
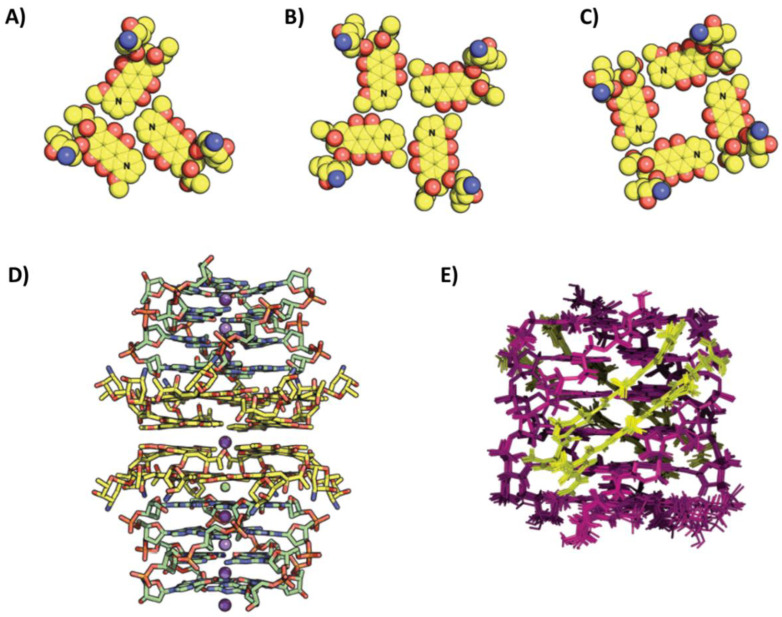
(**A**) Layer of daunomycin molecules in complex with the tetramolecular parallel G-quadruplex o-tel6 of sequence d(TGGGGT), as obtained by X-ray crystallography [76]. (**B**,**C**) Different layers of daunomycin molecules in complex with the tetramolecular parallel G-quadruplex o-tel4 of sequence d(GGGG), as obtained by X-ray crystallography [77]. (**D**) Crystal structure of the complex between the tetramolecular parallel G-quadruplex o-tel4 of sequence d(GGGG) and daunomycin [77]. G-quadruplex units are 5′-5′ stacked. (**E**) NMR structure of the complex between the tetramolecular parallel G-quadruplex o-tel6 of sequence d(TGGGGT) and distamycin [78]. 5′- and 3′-ends of the G-quadruplex are at the top and bottom, respectively. Adapted with permission from refs. [76,77,78], Copyright 2003 American Chemical Society and Copyright 2012 Oxford University Press and Copyright 2007 American Chemical Society.

The structure of the complex of daunomycin with the tetramolecular parallel G-quadruplex o-tel6 of sequence d(TGGGGT) was solved by X-ray crystallography (PDB ID: 1O0K) [76]. Three daunomycin molecules, held together in one layer by a cluster of Van der Waals contacts (Figure 9A), interacted with the 5′-end G-tetrad by stacking interactions. This layer was also stacked on a second layer of three daunomycin molecules, which, in turn, stacked on the 5′-end G-tetrad of a second G-quadruplex unit. The degree of anthraquinone cores overlap within the two daunomycin layers indicating stronger stacking interactions than those between the single daunomycin layer and the 5′-end G-tetrad of each G-quadruplex. Additionally, the three daunomycin molecules in each layer formed direct or water-mediated hydrogen bonds with the oligonucleotide backbone phosphates in the G-quadruplex grooves exploiting their sugar moiety [76].

In a successive study, the structure of daunomycin in complex with the tetramolecular parallel G-quadruplex o-tel4 of sequence d(GGGG) was also obtained by X-ray crystallography (PDB ID: 3TVB) in order to investigate how the ligand bound the G-quadruplex target in the absence of a putative thymine interference [77]. In this case, four daunomycin layers were observed within two G-quadruplex units, each consisting of four molecules stacked onto the 5′-end G-tetrad (Figure 9B–D). More in detail, the daunomycin layers exhibited two different arrangements: (i) in the outer layers, the methoxy ends (N) of each daunomycin molecule pointed towards the methoxy ends of the adjacent molecule (Figure 9B), while, in the inner layers, the methoxy ends pointed towards the acetyl ends (Figure 9C). Finally, contrary to the previous complex, no interaction of the ligands with the grooves was observed [77].

A completely different binding mode was found for distamycin complexed with the tetramolecular parallel G-quadruplex o-tel6 by NMR (PDB ID: 2JT7) [78]. In detail, four distamycin molecules were bound per G-quadruplex, forming two antiparallel dimers in two opposite grooves of the G-quadruplex structure (Figure 9E). Moreover, the two distamycin dimers spanned almost the entire length of the grooves, being slightly shifted towards the 5′-end, and their positively charged amidinium moiety interacted with the phosphate groups of G4 and G5 [78].

NMR studies allowed to investigate in detail the structure of the complex between the tetramolecular parallel G-quadruplex o-tel6 and a distamycin analog, where the amidinium group was replaced by an uncharged *N*-methylamide moiety (PDB ID: 2KVY) [79]. Analogously to distamycin, four molecules of this derivative bound a single G-quadruplex by forming two antiparallel dimers located in two opposite grooves. However, in this case, the dimers were shifted towards the 3′-end of the structure. Particularly, the dimers were inserted in the grooves mainly forming a network of hydrogen bonds, but they also interacted with the 3′-end of the G-quadruplex by hydrophobic interactions. Overall, the binding pose was quite different from that of distamycin due to the lack of the charged group. However, the binding constants for the two ligands to the G-quadruplex o-tel6 were similar [79].

#### 2.1.9. RNA Telomeric G-Quadruplexes

Contrary to what previously believed, telomeric DNA is not transcriptionally silent, but the C-rich strand of telomeric DNA is transcribed into telomeric repeat-containing RNA, named TERRA and involved in several cellular processes [80,81]. Particularly, TERRA sequence consists of r(UAAGGG) repeats, and several studies proved that it can fold in highly stable G-quadruplexes, which can represent specific structures to be targeted for therapeutic approaches [82,83].

**Figure 10:**
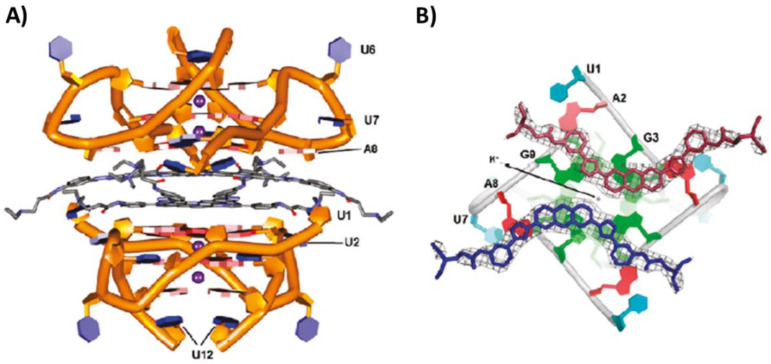
(**A**) Crystal structure of the complex between the bimolecular parallel G-quadruplex hr-tel12 of sequence r[(UAGGGU)_2_] and a 3,6-disubstituted acridine. G-quadruplex units are 5′-5′ stacked. Details of the ligand binding are highlighted in (**B**). Adapted with permission from ref. [84], Copyright 2011 American Chemical Society.

Interestingly, the crystal structure of a 3,6-disubstituted acridine, bearing triazole-phenyl-diethylamine side chains, in complex with the bimolecular parallel G-quadruplex hr-tel12 of sequence r[(UAGGGU)_2_] was solved (PDB ID: 3MIJ) [84]. A binding stoichiometry of 1:2 G-quadruplex/ligand was found, explained considering that the 5′-end G-tetrad of RNA G-quadruplex was surrounded by four adenines of the loops, forming an all-purine octet, thus generating a big surface platform where two acridine molecules could stack (Figure 10A,B) [84]. This arrangement was due to the presence of 2′-hydroxyl groups in RNA strands that were able to interact with the loop adenines by hydrogen bonds. Moreover, the two ligand molecules were stacked on additional two molecules which in turn stacked on the 5′-end G-tetrad of a second G-quadruplex unit (Figure 10A). The ligand, despite having a potentially high degree of intrinsic conformational flexibility, adopted a predominantly planar conformation due in large part to the high degree of overlap between its aromatic groups and the purine octet [84].

### 2.2. Oncogenic G-Quadruplexes

In addition to telomeres, DNA G-quadruplexes have been also found in promoter regions of oncogenes [85]. In-depth structural characterization of oncogenic G-quadruplexes/small-molecule ligands complexes was achieved for G-quadruplexes of C-MYC, RET, PDGFR-β and VEGF oncogene promoters, as described below.

#### 2.2.1. C-MYC Oncogene Promoter G-Quadruplexes

The unimolecular parallel G-quadruplex Pu24T of sequence d(TGAGGGTGGTGAGGGTGGGGAAGG) was chosen as a model to study C-MYC oncogene promoter G-quadruplex interactions with the bisquinolinium compound Phen-DC3 by NMR (PDB ID: 2MGN) [86]. The binding of the ligand occurred at the more accessible 5′-end G-tetrad by stacking interactions. The quinolinium moieties were perfectly stacked onto two guanine bases of the G-tetrad, while the phenanthroline core was stacked onto the other two guanines of the G-tetrad, thus reinforcing the overall interactions and explaining the high binding affinity of Phen-DC3 for G-quadruplexes [86].

On the other hand, the unimolecular parallel G-quadruplex Pu24I of sequence d(TGAGGGTGGIGAGGGTGGGGAAGG) was exploited as target for NMR studies with the cationic porphyrin TMPyP4 (PDB ID: 2A5R) [87]. One molecule of TMPyP4 interacted with the 5′-end G-tetrad, establishing stacking interactions with the guanines and electrostatic interactions with the negatively charged phosphate groups. In turn, the residues T1 and G2 were stacked on top of TMPyP4, contributing to the complex stability [87].

Additionally, solution NMR studies of the complex between berberine and the unimolecular parallel G-quadruplex Pu22 of sequence d(TGAGGGTGGGTAGGGTGGGGAA) were performed and the NMR structures related to two different ligand conformers were solved (PDB IDs: 7N7D and 7N7E, respectively) [88]. Berberine molecules bound at both 5′- and 3′-end G-tetrads, forming a 1:2 G-quadruplex/ligand complex. Interestingly, two different conformers of berberine were observed in each binding site, due to the possibility of each berberine molecule to interact with the G-tetrad by its two different surfaces, thus giving rise to four different complexes. However, in all cases, the positively charged convex side of the molecule pointed towards the center of each G-tetrad [88].

Moreover, the unimolecular parallel G-quadruplex Pu22T of sequence d(TGAGGGTGGGTAGGGTGGGTAA) was used as target for NMR studies with the ligand quindoline (PDB ID: 2L7V) [89]. A 1:2 G-quadruplex/ligand complex formed wherein the quindoline ligands were stacked at both 5′- and 3′-end G-tetrads. In both ends, upon ligand binding, the flanking bases changed their original conformation thus forming a pocket in which each ligand molecule could perfectly fit. The ligand binding at the 3′-end was also stabilized by a hydrogen bond with T23 flanking base. However, relevant differences were found in the two binding sites. Indeed, the 5′-end G-tetrad was more accessible, providing a larger hydrophobic surface, where the quindoline could more easily stack and interact stronger compared to the 3′-end G-tetrad [89].

Furthermore, two different structures with a quinoline derivative named PEQ, using both the unimolecular parallel G-quadruplexes Pu22 and Pu22T as models, were obtained by NMR (PDB IDs: 7KBW and 7KBX, respectively) [90]. PEQ bound both 5′- and 3′-end G-tetrads and, particularly, was stacked between the 5′-end G-tetrad and A6 flanking base, and stacked between the 3′-end G-tetrad and G20 or T20 flanking base in the case of Pu22 or Pu22T, respectively [90].

The unimolecular parallel G-quadruplex Pu22T was also studied by NMR in its interaction with a benzofuran derivative named DC-34 (PDB ID: 5W77), which showed an efficient and specific down-regulation of C-MYC transcription in cancer cells [91]. DC-34 stacked on both the 5′- and 3′-end G-tetrads by its benzofuran and methylbenzene rings. Notably, both the 5′- and 3′-end flanking bases changed their native positions and generated hydrophobic-binding pockets accommodating the bound ligands. Additionally, hydrogen bonds proved to be crucial for binding specificity and were formed between the oxygen of the benzofuran core and the amino group of A25, as well as between the fluorine atoms at the para-position of the benzene ring and the amino groups of G7 and G18 [91].

**Figure 11:**
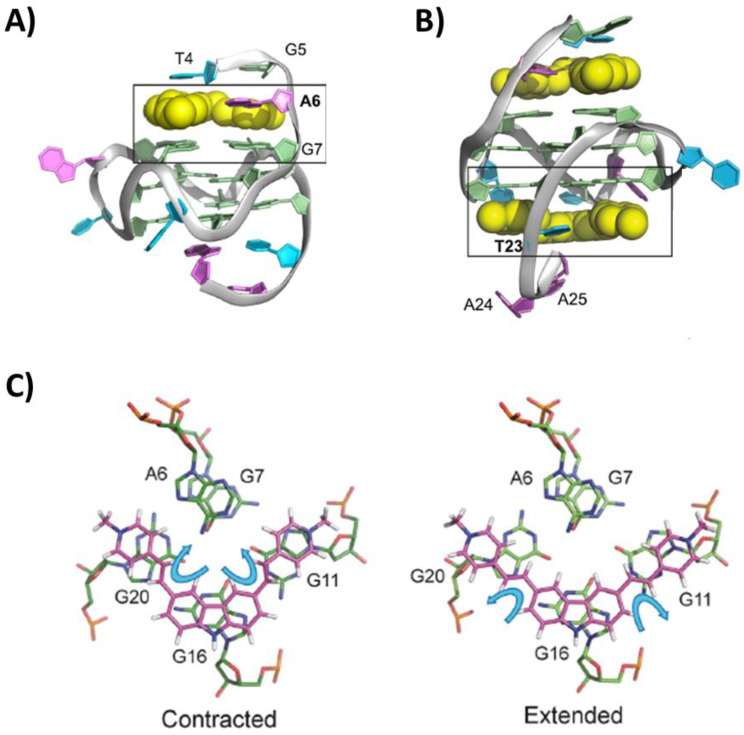
NMR structure of the complex between the unimolecular parallel G-quadruplex Pu22T of sequence d(TGAGGGTGGGTAGGGTGGGTAA) and BMVC at (**A**) 1:1 or (**B**) 1:2 G-quadruplex/ligand stoichiometry. 5′- and 3′-ends of the G-quadruplex are at the top and bottom, respectively. (**C**) Comparison between the contracted and extended conformation of BMVC found for its G-quadruplex-bound and free forms. Adapted with permission from ref. [92], Copyright 2019 Oxford University Press.

Another ligand showing high and specific ability to repress C-MYC expression in cancer cells was the carbazole derivative BMVC. The structure of its complex with the unimolecular parallel G-quadruplex Pu22T was solved by NMR (PDB IDs: 6JJ0 and 6O2L related to the 1:1 and 1:2 G-quadruplex/ligand complexes, respectively) [92]. BMVC first bound the 5′-end G-tetrad by stacking interactions and recruiting A6 flanking base to form a ligand–base pair that anchored its position and was responsible of the high specificity and affinity (Figure 11A). On the other hand, the 3′-end G-tetrad was also targeted only when the ligand was in excess, due to the lower affinity of the ligand for this binding site and higher dynamicity of the binding. Additionally, in this case, a flanking base, i.e., T23, rearranged to form a ligand–base pair (Figure 11B). Notably, BMVC flexibility allowed a contraction of its structure (Figure 11C), thus maximizing the stacking interactions [92].

Finally, a complex of the unimolecular parallel G-quadruplex Pu19 of sequence d(TAGGGAGGGTAGGGAGGGT) with the triangulenium derivative DAOTA-M2 was solved by NMR (PDB ID: 5LIG) [93]. DAOTA-M2 is an optical probe showing the notable property of displaying significantly longer fluorescence lifetimes when bound to G-quadruplexes than duplex DNA. This ligand bound both the 5′- and 3′-end G-tetrads by stacking with its polyaromatic core. More in detail, it was sandwiched between T1 and the 5′-end G-tetrad, as well as between T19 and the 3′-end G-tetrad. However, at 5′-end it exhibited higher flexibility than when bound to the 3′-end [93].

#### 2.2.2. RET Oncogene Promoter G-Quadruplexes

**Figure 12:**
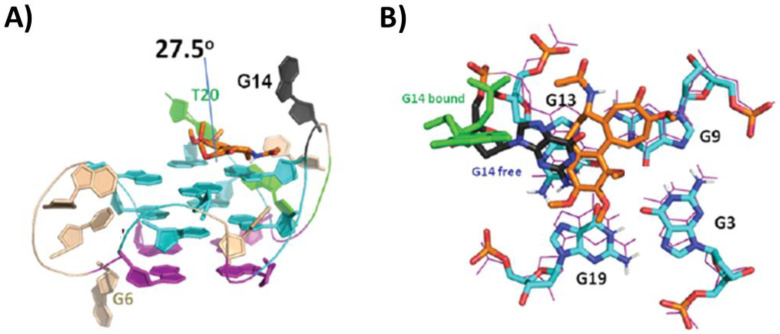
(**A**) NMR structure of the complex between the unimolecular parallel G-quadruplex RET of sequence d(GGGGCGGGGCGGGGCGGGGT) and colchicine. 3′- and 5′-end of the G-quadruplex are at the top and bottom, respectively. Details of the ligand binding are highlighted in (**B**). Adapted with permission from ref. [94], Copyright 2020 The Royal Society of Chemistry.

By in silico and 1D NMR studies, a highly specific binding of colchicine to the G-quadruplex formed by RET oncogene promoter was proved [94]. Based on these results, more in-depth NMR studies were performed, and the solution structure of the complex between the unimolecular parallel G-quadruplex RET of sequence d(GGGGCGGGGCGGGGCGGGGT) and colchicine was solved (PDB ID: 6JWE) [94]. A 1:1 G-quadruplex/ligand complex was formed, wherein colchicine interacted by stacking with the 3′-end G-tetrad and the seven-membered and phenyl rings of colchicine formed an angle of 27.5° (Figure 12A). Notably, in the absence of a ligand, the G14 residue played a key role in the G-quadruplex stability, stacking on G13 of the 3′-end G-tetrad and forming a hydrogen bond with G19. On the other hand, in the complex with colchicine, the ligand displaced the G14, playing a stabilizing role which provides a rational behind its high selectivity for the unimolecular parallel G-quadruplex RET (Figure 12B). Indeed, another G-quadruplex ligand, i.e., berberine, whose NMR structure with the same G-quadruplex was also solved (PDB ID: 6JWD), did not exhibit the ability to displace G14 from its native position, and accordingly showed lower specificity for the G-quadruplex RET than colchicine [94].

#### 2.2.3. PDGFR-β Oncogene Promoter G-Quadruplexes

**Figure 13:**
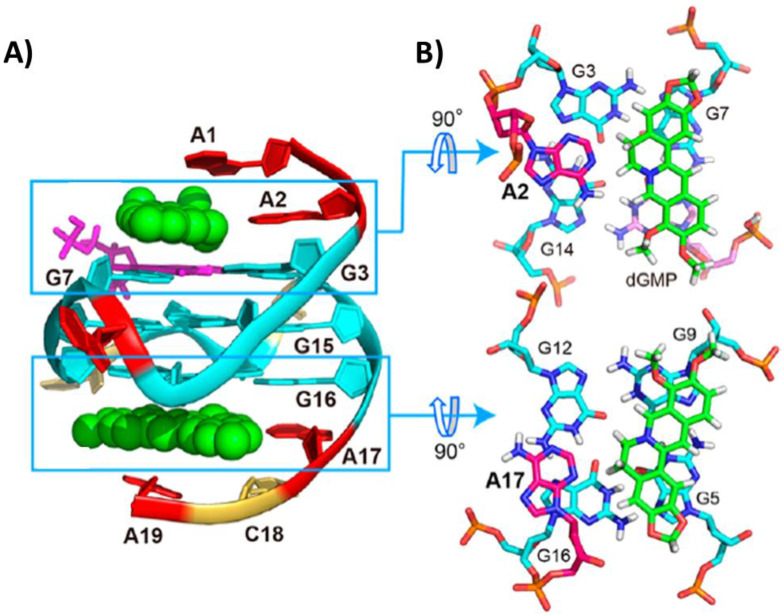
(**A**) NMR structure of the ternary complex formed by the unimolecular parallel G-quadruplex PDGFR-β of sequence d(AAGGGAGGGCGGCGGGACA), one dGMP molecule and berberine. Details of the berberine binding are highlighted in (**B**). Adapted with permission from ref. [96], Copyright 2021 American Chemical Society.

The unimolecular parallel G-quadruplex PDGFR-β of sequence d(AAGGGAGGGCGGCGGGACA) is featured by a vacancy in the 5′-end G-tetrad. Considering this peculiar characteristic, both the complex of G-quadruplex PDGFR-β with one dGMP molecule and the ternary complex among the G-quadruplex PDGFR-β, dGMP and berberine were investigated and their structures solved by NMR (PDB IDs: 6V0L and 7MSV, respectively) [95,96]. In the ternary complex, the dGMP molecule filled the vacancy. In turn, berberine interacted with both the 5′- and 3′-end G-tetrads by stacking and electrostatic interactions between the positively charged quaternary nitrogen of berberine and the negatively polarized tetrad-guanine carbonyl groups (Figure 13A). Additionally, A2 and A17 flanking residues were recruited at each end to form pseudo-planes with the ligand molecules that stacked onto the G-tetrads (Figure 13B) [96].

#### 2.2.4. VEGF Oncogene Promoter G-Quadruplexes

**Figure 14:**
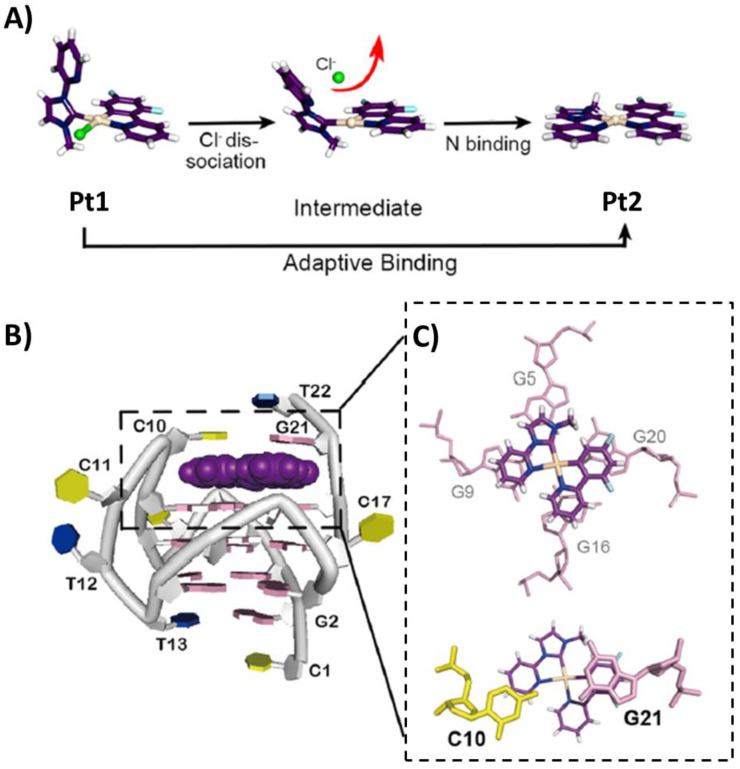
(**A**) Pt1 conversion to Pt2. (**B**) NMR structure of the complex between the unimolecular parallel G-quadruplex VEGF of sequence d(CGGGGCGGGCCTTGGGCGGGGT) and Pt2. 3′- and 5′-end of the G-quadruplex are at the top and bottom, respectively. Details of the ligand binding are highlighted in (**C**). Adapted with permission from ref. [97], Copyright 2021 Wiley-VCH GmbH.

A Pt(II) complex (Pt1) coordinated by a bidentate cyclometalated ligand, a monodentate *N*-heterocyclic carbene ligand and a chloride anion, able to repress VEGF expression in cancer cells, was studied in its interaction with the unimolecular parallel G-quadruplex VEGF of sequence d(CGGGGCGGGCCTTGGGCGGGGT). An analysis of this NMR structure (PDB ID: 6LNZ) [97] showed that the platinum complex lost its chloride ligand and then its overall structure changed, because the monodentate carbene interacted with the metal center through the *N*-pyridine residue (Figure 14A), forming a perfectly planar ligand (Pt2). This planar conformation given by the new Pt-N bond allowed optimized stacking interactions with all four 3′-end G-tetrad guanine residues (Figure 14B,C), with Pt2 overall showing an adaptive binding to the G-quadruplex VEGF. Moreover, C10 and G21 residues stacked on top of Pt2, thus further stabilizing the complex (Figure 14C) [97].

### 2.3. Viral G-Quadruplexes

**Figure 15:**
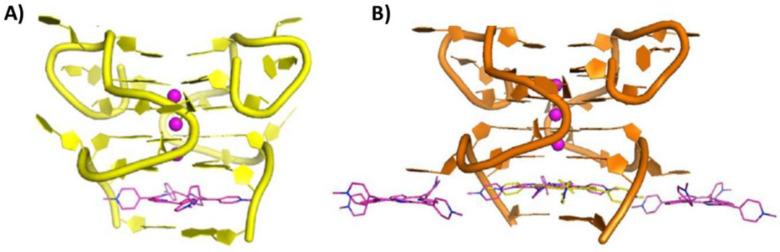
Crystal structure of the complex between the bimolecular parallel G-quadruplex IE180 of sequence r(GGCUCGGCGGCGGA) and TMPyP4 at (**A**) 1:1 or (**B**) 1:2 G-quadruplex/ligand stoichiometry. 5′- and 3′-ends of the G-quadruplex are at the top and bottom, respectively. Adapted with permission from ref. [98], Copyright 2020 Oxford University Press.

The structure of the complex between TMPyP4 and the bimolecular parallel RNA G-quadruplex of sequence r(GGCUCGGCGGCGGA), from the IE180 gene regulating the Pseudorabies virus (PRV) replication, was obtained by X-ray crystallography (PDB IDs: 6JJI and 6JJH related to 1:1 and 1:2 G-quadruplex/ligand complexes, respectively) [98]. This viral RNA G-quadruplex was very unusual, as it presented the two different strands in an interlocked arrangement, thus forming four G-tetrad stacked planes. In the 1:1 G-quadruplex/ligand complex, the ligand bound to the 3′-end G-tetrad, being sandwiched between the G-tetrad and an AA-coupling plane formed by two flanking adenines (Figure 15A). On the other hand, in the 1:2 G-quadruplex/ligand complex, one ligand molecule bound to the pocket between the 3′-end G-tetrad and the AA-coupling plane, as in the 1:1 complex, while the second ligand molecule stacked between a pair of cytosines and a pair of uracils of two lattice-related G-quadruplexes (Figure 15B) [98].

## 3. Summary and Outlook

The existence and significant biological role of G-quadruplex structures have been fully ascertained in several genomes. Indeed, both DNA and RNA G-quadruplexes turned out to be important targets for the development of novel, effective anticancer and antiviral therapies [1,2,3,4]. In this framework, more than 3000 small-molecule ligands for G-quadruplexes have been developed in the last decades as potential anticancer and/or antiviral drugs [19,20,21,22,23], and for some of them, NMR and/or crystallographic studies were performed providing in-depth knowledge on their interactions with diverse G-quadruplex targets. Here, all the structures solved thus far and deposited as PDB files have been described, and the summary of all the collected data is schematically reported in Table 1 and Table S1.

Overall, it emerged that crystal packing favors the formation of G-quadruplex dimers, 5′-5′ stacked, wherein ligands can interact only with the 3′-end G-tetrads, or only with the 5′-5′ interface between the G-quadruplex units or, in other cases, with both the 3′- and 5′-end G-tetrads. On the other hand, G-quadruplex dimer formation was observed by NMR only in one case, i.e., with the ligand Pt(II)-based tripod, which, interestingly, has the peculiar ability to induce the formation in solution of a 3′-3′ G-quadruplex dimer, in contrast to what observed in crystals, wherein the ligand bound both to 5′- and 3′-end G-tetrads [40].

Moreover, despite the high prevalence of end-stacking binding mode for G-quadruplex ligands, groove binding has been observed in the case of tetramolecular parallel G-quadruplexes with adriamycin, epirubicin and distamycin derivatives [63,64,78,79]. More notably, the intercalative binding mode, which has never been observed due to the presence of the coordinating cation between two G-tetrads, has been recently reported for Phen-DC3 with a telomeric unimolecular G-quadruplex target [42].

Furthermore, when the target adopts hybrid-1 or hybrid-2 topology, the preferential binding site for the ligands, regardless of the nature of the ligand, appears to be the 5′-end G-tetrad.

Additional general conclusions can be inferred for the most investigated families of ligands, i.e., acridines and naphthalene diimides. Indeed, both families of ligands showed a conservative binding mode: acridines preferentially target the 5′-end G-tetrad, while NDIs typically target the 3′-end G-tetrad in 1:1 G-quadruplex/ligand complexes, while, at a higher ligand concentration, they also target the 5′-end G-tetrad and/or groove/loop regions.

Interestingly, some ligands showed the ability to change their conformation upon G-quadruplex binding, either by modifying the bent geometry or space compactness of their free form, as in the case of telomestatin derivative L2H2-6M(2)OTD [41], porphyrin derivative NMM [50] and carbazole derivative BMVC [92], or even exhibiting exchange of the coordinating ligands, as in the case of the Pt(II) complex Pt1 [97], finally providing adaptive binding with the highest overlapping of the ligand aromatic groups and the G-tetrad surface so to produce the strongest stacking interactions.

Altogether, the large body of structural data here summarized provides fundamental knowledge for the rational design of novel and more selective G-quadruplex targeting ligands. Notwithstanding the outstanding advances in this field, only a very limited number of small molecules effective as G-quadruplex binders entered human clinical trials and no one has been approved as a drug yet. Therefore, the development of highly effective anticancer/antiviral drugs but also of diagnostic tools for selective G-quadruplex detection still needs further, targeted scientific efforts, which have to be based on solid and detailed structural information, crucial to obtaining the required specific recognition. In this regard, the advent of in-cell NMR to characterize G-quadruplex structures and G-quadruplex/ligand interactions could significantly complement NMR and crystallographic studies, thus providing crucial information on the target/drug interactions in the cellular context [99,100,101].

**Table 1:** Summary of the features of ligands and G-quadruplex targets within the NMR and crystal structures solved thus far for G-quadruplex/small-molecule ligand complexes.

PDB ID	Technique	Ligand Class	Ligand Family	Ligand Name	Sequence	Sequence Name	Prevalent Cation	Molecularity	Topology	Binding Mode (G-Quadruplex/ Ligand Stoichiometry)	Binding Interactions	Refs.
**Telomeric G-quadruplexes**												
**Human telomeric unimolecular antiparallel G-quadruplexes**												
2MCO	NMR	Metal-organic	Dinuclear Ru(II) complex	ΛΛ-[{Ru(phen)_2_}_2_tpphz]^4+^	d[AGGG(TTAGGG)_3_]	h-tel22	Na^+^	Unimolecular	Antiparallel basket	Intercalation between 5′-end G-tetrad and diagonal loop (1:1)	Stacking and electrostatic interactions	[31]
2MCC	NMR	Metal-organic	Dinuclear Ru(II) complex	ΔΔ-[{Ru(phen)_2_}_2_tpphz]^4+^	d[AGGG(TTAGGG)_3_]	h-tel22	Na^+^	Unimolecular	Antiparallel basket	3′-end G-tetrad stacking (1:1)	Stacking	[31]
7OTB	X-ray crystallography	Metal-organic	Ru(II) complex	Λ-[Ru(phen)_2_(qdppz)]^2+^	d[GGG(TTAGGG)_2_TTTGGG]	h-tel21	Na^+^	Unimolecular	Antiparallel chair	5′-end G-tetrad stacking (1:1)	Stacking	[33]
**Human telomeric unimolecular hybrid G-quadruplexes**												
6CCW	NMR	Organic	Berberine	Epiberberine	d[(TTAGGG)_4_TT]	h-tel26	K^+^	Unimolecular	Hybrid-2	Intercalation between 5′-end G-tetrad and T2:T13:A15 triad layer and T1:T14 pair (1:1)	Stacking and hydrogen bonds	[37]
5MVB	NMR	Metal-organic	Dinuclear Au(III) complex	Auoxo6	d[(TTAGGG)_4_TT]	h-tel26	K^+^	Unimolecular	Hybrid-2	Intercalation between 5′-end G-tetrad and flanking A3 (1:1)	Stacking	[38]
6KFJ	NMR	Organic	Tripod	NBTE	d[(TTAGGG)_4_TT]	h-tel26	K^+^	Unimolecular	Hybrid-2	Intercalation between 5′-end G-tetrad and capping triad A3, T14, A21 (1:1)	Stacking, π-cation and electrostatic interactions	[39]
6KFI	NMR	Organic	Tripod	NBTE	d[AAAGGG(TTAGGG)_3_AA]	h-tel26A	K^+^	Unimolecular	Hybrid-1	Intercalation between 5′-end G-tetrad and capping triad A3, A9, T20 (1:1)	Stacking, π-cation and electrostatic interactions	[39]
5Z80	NMR	Metal-organic	Pt(II) complex	Pt(II)-based tripod	d[AAAGGG(TTAGGG)_3_AA]	h-tel26A	K^+^	Unimolecular	Hybrid-1	Intercalation between 5′-end G-tetrad and capping triad A3, A9, T20 (1:1)	Stacking, hydrogen bonds and electrostatic interactions	[40]
5Z8F	NMR	Metal-organic	Pt(II) complex	Pt(II)-based tripod	d[AAAGGG(TTAGGG)_3_AA]	h-tel26A	K^+^	Unimolecular	Hybrid-1	5′- and 3′-end G-tetrad stacking within a 3′-3′ dimer (2:4)	Stacking, hydrogen bonds and electrostatic interactions	[40]
2MB3	NMR	Organic	Telomestatin	L2H2-6M(2)OTD	d[TTGGG(TTAGGG)_3_A]	h-tel24	K^+^	Unimolecular	Hybrid-1	5′-end G-tetrad stacking (1:1)	Stacking and electrostatic interactions	[41]
7Z9L	NMR	Organic	Phenanthroline-quinoline	Phen-DC3	d[TAGGG(TTAGGG)_3_]	h-tel23	K^+^	Unimolecular	From Hybrid-1 to Antiparallel chair	Intercalation between a two-tetrad unit and a 5′-end “pseudo-tetrad” (1:1)	Stacking	[42]
**Human telomeric unimolecular parallel G-quadruplexes**												
3R6R	X-ray crystallography	Organic	Berberine	Berberine	d[TAGGG(TTAGGG)_3_]	h-tel23	K^+^	Unimolecular	Parallel	5′- and 3′-end G-tetrad stacking within a 5′-5′ dimer (1:3)	Stacking and hydrogen bonds	[43]
5CCW	X-ray crystallography	Metal-organic	Au(I) complex	[Au(9-methylcaffein-8-ylidene)_2_]^+^	d[TAGGG(TTAGGG)_3_]	h-tel23	K^+^	Unimolecular	Parallel	5′- and 3′-end G-tetrad stacking (1:3)	Stacking	[44]
6H5R	X-ray crystallography	Metal-organic	Au(I) complex	[Au(1-butyl-3-methyl-2-ylidene)_2_]^+^	d[TAGGG(TTAGGG)_3_T]	h-tel24	K^+^	Unimolecular	Parallel	5′-end G-tetrad stacking (1:1)	Stacking	[45]
3CDM	X-ray crystallography	Organic	Naphthalene diimide	NDI-1	d[TAGGG(TTAGGG)_3_]	h-tel23	K^+^	Unimolecular	Parallel	5′- and 3′-end G-tetrad stacking within a 5′-5′ dimer and loop binding (1:6)	Stacking	[47]
3SC8	X-ray crystallography	Organic	Naphthalene diimide	BMSG-SH3	d[AGGG(TTAGGG)_3_]	h-tel22	K^+^	Unimolecular	Parallel	3′-end G-tetrad stacking within a 5′-5′ dimer (1:1)	Stacking and electrostatic interactions	[48]
3T5E	X-ray crystallography	Organic	Naphthalene diimide	BMSG-SH4	d[AGGG(TTAGGG)_3_]	h-tel22	K^+^	Unimolecular	Parallel	3′-end G-tetrad stacking within a 5′-5′ dimer (1:1)	Stacking and electrostatic interactions	[48]
3UYH	X-ray crystallography	Organic	Naphthalene diimide	3d	d[AGGG(TTAGGG)_3_]	h-tel22	K^+^	Unimolecular	Parallel	3′-end G-tetrad stacking within a 5′-5′ dimer (1:1)	Stacking and electrostatic interactions	[49]
4DA3	X-ray crystallography	Organic	Naphthalene diimide	3d	d[GGG(TTAGGG)_3_]	h-tel21	K^+^	Unimolecular	Parallel	3′-end G-tetrad stacking within a 5′-5′ dimer (1:1)	Stacking and electrostatic interactions	[49]
4DAQ	X-ray crystallography	Organic	Naphthalene diimide	BMSG-SH3	d[GGG(TTAGGG)_3_]	h-tel21	K^+^	Unimolecular	Parallel	3′-end G-tetrad stacking within a 5′-5′ dimer (1:1)	Stacking and electrostatic interactions	[49]
4FXM	X-ray crystallography	Organic	Porphyrin	*N*-methyl mesoporphyrin IX	d[AGGG(TTAGGG)_3_]	h-tel22	K^+^	Unimolecular	Parallel	3′-end G-tetrad stacking within a 5′-5′ dimer (1:1)	Stacking	[50]
4G0F	X-ray crystallography	Organic	Porphyrin	*N*-methyl mesoporphyrin IX	d[AGGG(TTAGGG)_3_]	h-tel22	K^+^	Unimolecular	Parallel	3′-end G-tetrad stacking within a 5′-5′ dimer (1:1)	Stacking	[50]
6XCL	X-ray crystallography	Metal-organic	Pt(II) complex	Pt(II)-based ligand	d[AGGG(TTAGGG)_3_]	h-tel22	K^+^	Unimolecular	Parallel	5′- and 3′-end G-tetrad stacking within a 5′-5′ dimer (2:3)	Stacking	[53]
**Human telomeric bimolecular parallel G-quadruplexes**												
5CDB	X-ray crystallography	Organic	Berberine	NAX053	d(TAGGGTTAGGGT)	h-tel12	K^+^	Bimolecular	Parallel	Intercalation between 3′-end G-tetrad and 5′-end T:A:T:A tetrad (2:3)	Stacking	[54]
6S15	X-ray crystallography	Organic	Berberine	13-alkylpyridine berberine	d(TAGGGTTAGGGT)	h-tel12	K^+^	Bimolecular	Parallel	Intercalation between 3′-end G-tetrad and 5′-end T:A:T:A tetrad (2:3)	Stacking	[55]
3CCO	X-ray crystallography	Organic	Naphthalene diimide	NDI-1	d(TAGGGTTAGGGT)	h-tel12	K^+^	Bimolecular	Parallel	3′-end G-tetrad stacking and loop binding (1:3)	Stacking	[47]
3CE5	X-ray crystallography	Organic	Acridine	BRACO-19	d(TAGGGTTAGGGT)	h-tel12	K^+^	Bimolecular	Parallel	Intercalation between 3′-end G-tetrad and 5′-end T:A:T:A tetrad (1:2)	Stacking and hydrogen bonds	[58]
2HRI	X-ray crystallography	Organic	Porphyrin	TMPyP4	d(TAGGGTTAGGG)	h-tel11	K^+^	Bimolecular	Parallel	Flanking residues and loop binding (1:2)	Stacking	[59]
3QSC	X-ray crystallography	Metal-organic	Cu(II) complex	Cu(II) salphen metal complex	d(AGGGTBrUAGGTT)	h-tel11Br	K^+^	Bimolecular	Parallel	3′-end G-tetrad stacking within a 5′-5′ dimer (1:1)	Stacking	[60]
3QSF	X-ray crystallography	Metal-organic	Ni(II) complex	Ni(II) salphen metal complex	d(AGGGTBrUAGGTT)	h-tel11Br	K^+^	Bimolecular	Parallel	3′-end G-tetrad stacking within a 5′-5′ dimer (1:1)	Stacking	[60]
**Human telomeric tetramolecular parallel G-quadruplexes**												
5LS8	X-ray crystallography	Metal-organic	Ru(II) complex	Λ/Δ-[Ru(TAP)_2_(11-CN-dppz)]^2+^	d(TAGGGTTA)	h-tel8	K^+^	Tetramolecular	From Parallel to Antiparallel	5′- and 3′-end binding (1:4 and 1:2)	Stacking	[61]
6RNL	X-ray crystallography	Metal-organic	Ru(II) complex	Λ-[Ru(TAP)_2_(dppz)]^2+^	d(TAGGGTT)	h-tel7	K^+^	Tetramolecular	Parallel	Flanking residues binding (1:4)	Stacking	[62]
6KXZ	NMR	Organic	Anthracyclin	Epirubicin	d(TTAGGGT)	h-tel7	K^+^	Tetramolecular	Parallel	Groove binding (1:2)	Hydrogen bonds	[63]
6KN4	NMR	Organic	Anthracyclin	Adriamycin	d(TTAGGGT)	h-tel7	K^+^	Tetramolecular	Parallel	Groove binding (1:2)	Stacking and hydrogen bonds	[64]
2MS6	NMR	Organic	Flavonoid	Quercetin	d(TTAGGGT)	h-tel7	K^+^	Tetramolecular	Parallel	Stacking between T1 and T2 tetrads and between 3′-end G-tetrad and T7 tetrad (1:2)	Stacking	[67]
2JWQ	NMR	Organic	Quinacridine	MMQ1	d(TTAGGGT)	h-tel7	K^+^	Tetramolecular	Parallel	5′- and 3′-end G-tetrad stacking (1:2)	Stacking and electrostatic interactions	[68]
1NZM	NMR	Organic	Acridine	RHPS4	d(TTAGGGT)	h-tel7	K^+^	Tetramolecular	Parallel	5′- and 3′-end G-tetrad stacking (1:2)	Stacking and electrostatic interactions	[69]
**Oxytricha telomeric unimolecular parallel G-quadruplexes**												
6P45	X-ray crystallography	Organic	Porphyrin	*N*-methyl mesoporphyrin IX	d[(TGGGT)_4_]	o-tel20	K^+^	Unimolecular	Parallel	3′-end G-tetrad stacking within a 5′-5′ dimer (1:1)	Stacking	[70]
6PNK	X-ray crystallography	Organic	Porphyrin	*N*-methyl mesoporphyrin IX	d[(GGGTT)_3_GGG]	o-tel18	K^+^	Unimolecular	Parallel	3′-end G-tetrad stacking within a 5′-5′ dimer (1:1)	Stacking	[70]
6K3X	NMR	Organic	Dinucleotide	d(AG)	d[TTGGT(GGGT)_3_]	o-tel17	K^+^	Unimolecular	Parallel	G-vacancy filling (1:1)	Stacking and hydrogen bonds	[71]
6K3Y	NMR	Organic	Dinucleotide	cGAMP	d[TTGGT(GGGT)_3_]	o-tel17	K^+^	Unimolecular	Parallel	G-vacancy filling (1:1)	Stacking and hydrogen bonds	[71]
**Oxytricha telomeric bimolecular antiparallel G-quadruplexes**												
1L1H	X-ray crystallography	Organic	Acridine	BSU6039	d(GGGGTTTTGGGG)	o-tel12	K^+^	Bimolecular	Antiparallel	Intercalation between 5′-end G-tetrad and T3 (1:1)	Stacking and hydrogen bonds	[72]
3EM2	X-ray crystallography	Organic	Acridine	3-morpholinopropionamido 3,6-disubstituted acridine derivative	d(GGGGTTTTGGGG)	o-tel12	K^+^	Bimolecular	Antiparallel	Intercalation between 5′-end G-tetrad and T3 (1:1)	Stacking and hydrogen bonds	[73]
3EQW	X-ray crystallography	Organic	Acridine	3-(2-ethylpiperidino)propionamido 3,6-disubstituted acridine derivative	d(GGGGTTTTGGGG)	o-tel12	K^+^	Bimolecular	Antiparallel	Intercalation between 5′-end G-tetrad and T3 (1:1)	Stacking and hydrogen bonds	[73]
3EUI	X-ray crystallography	Organic	Acridine	3-(2-ethylpiperidino)propionamido 3,6-disubstituted acridine derivative	d(GGGGTTTTGGGG)	o-tel12	K^+^	Bimolecular	Antiparallel	Intercalation between 5′-end G-tetrad and T3 (1:1)	Stacking and hydrogen bonds	[73]
3ERU	X-ray crystallography	Organic	Acridine	3-(2-methylpiperidino)propionamido 3,6-disubstituted acridine derivative	d(GGGGTTTTGGGG)	o-tel12	K^+^	Bimolecular	Antiparallel	Intercalation between 5′-end G-tetrad and T3 (1:1)	Stacking and hydrogen bonds	[73]
3ES0	X-ray crystallography	Organic	Acridine	3-(4-methylpiperidino)propionamido 3,6-disubstituted acridine derivative	d(GGGGTTTTGGGG)	o-tel12	K^+^	Bimolecular	Antiparallel	Intercalation between 5′-end G-tetrad and T3 (1:1)	Stacking and hydrogen bonds	[73]
3ET8	X-ray crystallography	Organic	Acridine	3-(3-methylpiperidino)propionamido groups 3,6-disubstituted acridine derivative	d(GGGGTTTTGGGG)	o-tel12	K^+^	Bimolecular	Antiparallel	Intercalation between 5′-end G-tetrad and T3 (1:1)	Stacking and hydrogen bonds	[73]
3EUM	X-ray crystallography	Organic	Acridine	3-(azepan-1-yl)propionamido 3,6-disubstituted acridine derivative	d(GGGGTTTTGGGG)	o-tel12	K^+^	Bimolecular	Antiparallel	Intercalation between 5′-end G-tetrad and T3 (1:1)	Stacking and hydrogen bonds	[73]
3NYP	X-ray crystallography	Organic	Acridine	bis-3-fluoropyrrolidine enantiomer (*R,R*)	d(GGGGTTTTGGGG)	o-tel12	K^+^	Bimolecular	Antiparallel	Intercalation between 5′-end G-tetrad and T3 (1:1)	Stacking and electrostatic interactions	[74]
3NZ7	X-ray crystallography	Organic	Acridine	bis-3-fluoropyrrolidine enantiomer (*S,S*)	d(GGGGTTTTGGGG)	o-tel12	K^+^	Bimolecular	Antiparallel	Intercalation between 5′-end G-tetrad and T3 (1:1)	Stacking and electrostatic interactions	[74]
5HIX	X-ray crystallography	Organic	Quinoline	Oligoamide foldamer	d(GGGGTTTTGGGG)	o-tel12	K^+^	Bimolecular	Antiparallel	Loop binding (1:1)	Electrostatic interactions	[75]
**Oxytricha telomeric tetramolecular parallel G-quadruplexes**												
1O0K	X-ray crystallography	Organic	Anthracyclin	Daunomycin	d(TGGGGT)	o-tel6	Na^+^	Tetramolecular	Parallel	5′-end G-tetrad stacking within a 5′-5′ dimer (1:3)	Stacking and hydrogen bonds	[76]
3TVB	X-ray crystallography	Organic	Anthracyclin	Daunomycin	d(GGGG)	o-tel4	Na^+^	Tetramolecular	Parallel	5′-end G-tetrad stacking within a 5′-5′ dimer (1:4)	Stacking	[77]
2JT7	NMR	Organic	Distamycin	Distamycin	d(TGGGGT)	o-tel6	K^+^	Tetramolecular	Parallel	Groove binding (1:4)	Electrostatic interactions	[78]
2KVY	NMR	Organic	Distamycin	*N*-methyl amide distamycin derivative	d(TGGGGT)	o-tel6	K^+^	Tetramolecular	Parallel	Groove binding (1:4)	Hydrogen bonds	[79]
**RNA telomeric G-quadruplexes**												
3MIJ	X-ray crystallography	Organic	Acridine	triazole-phenyl-diethylamine 3,6-disubstituted acridine	r[(UAGGGU)_2_]	hr-tel12	K^+^	Bimolecular	Parallel	5′-end G-tetrad stacking within a 5′-5′ dimer (1:2)	Stacking	[84]
**Oncogenic G-quadruplexes**												
**C-MYConcogene promoter G-quadruplexes**												
2MGN	NMR	Organic	Phenanthroline-quinoline	Phen-DC3	d(TGAGGGTGGTGAGGGTGGGGAAGG)	Pu24T	K^+^	Unimolecular	Parallel	5′-end G-tetrad stacking (1:1)	Stacking	[86]
2A5R	NMR	Organic	Porphyrin	TMPyP4	d(TGAGGGTGGIGAGGGTGGGGAAGG)	Pu24I	K^+^	Unimolecular	Parallel	Intercalation between 5′-end G-tetrad and flanking T1 and G2 (1:1)	Stacking and electrostatic interactions	[87]
7N7D	NMR	Organic	Berberine	Berberine	d(TGAGGGTGGGTAGGGTGGGGAA)	Pu22	K^+^	Unimolecular	Parallel	5′- and 3′-end G-tetrad stacking (1:2)	Stacking	[88]
7N7E	NMR	Organic	Berberine	Berberine	d(TGAGGGTGGGTAGGGTGGGGAA)	Pu22	K^+^	Unimolecular	Parallel	5′- and 3′-end G-tetrad stacking (1:2)	Stacking	[88]
2L7V	NMR	Organic	Quindoline	Quindoline	d(TGAGGGTGGGTAGGGTGGGTAA)	Pu22T	K^+^	Unimolecular	Parallel	5′-end G-tetrad stacking and intercalation between 3′-end G-tetrad and flanking T23 (1:2)	Stacking	[89]
7KBW	NMR	Organic	Quinoline	PEQ	d(TGAGGGTGGGTAGGGTGGGGAA)	Pu22	K^+^	Unimolecular	Parallel	Intercalation between 5′-end G-tetrad and flanking A6 and between 3′-end G-tetrad and flanking G20 (1:2)	Stacking	[90]
7KBX	NMR	Organic	Quinoline	PEQ	d(TGAGGGTGGGTAGGGTGGGTAA)	Pu22T	K^+^	Unimolecular	Parallel	Intercalation between 5′-end G-tetrad and flanking A6 and between 3′-end G-tetrad and flanking T20 (1:2)	Stacking	[90]
5W77	NMR	Organic	Benzofuran	DC-34	d(TGAGGGTGGGTAGGGTGGGTAA)	Pu22T	K^+^	Unimolecular	Parallel	5′- and 3′-end G-tetrad stacking (1:2)	Stacking and hydrogen bonds	[91]
6JJ0	NMR	Organic	Carbazole	BMVC	d(TGAGGGTGGGTAGGGTGGGTAA)	Pu22T	K^+^	Unimolecular	Parallel	5′-end G-tetrad stacking (1:1)	Stacking	[92]
6O2L	NMR	Organic	Carbazole	BMVC	d(TGAGGGTGGGTAGGGTGGGTAA)	Pu22T	K^+^	Unimolecular	Parallel	5′- and 3′-end G-tetrad stacking (1:2)	Stacking	[92]
5LIG	NMR	Organic	Triangulenium	DAOTA-M2	d(TAGGGAGGGTAGGGAGGGT)	Pu19	K^+^	Unimolecular	Parallel	Intercalation between 5′-end G-tetrad and flanking T1 and between 3′-end G-tetrad and flanking T19 (1:2)	Stacking	[93]
**REToncogene promoter G-quadruplexes**												
6JWE	NMR	Organic	Colchicine	Colchicine	d(GGGGCGGGGCGGGGCGGGGT)	RET	K^+^	Unimolecular	Parallel	3′-end G-tetrad stacking (1:1)	Stacking	[94]
6JWD	NMR	Organic	Berberine	Berberine	d(GGGGCGGGGCGGGGCGGGGT)	RET	K^+^	Unimolecular	Parallel	3′-end G-tetrad stacking (1:1)	Stacking	[94]
**PDGFR-βoncogene promoter G-quadruplexes**												
6V0L	NMR	Organic	Nucleotide	dGMP	d(AAGGGAGGGCGGCGGGACA)	PDGFR-β	K^+^	Unimolecular	Parallel	G-vacancy filling (1:1)	Stacking and hydrogen bonds	[95]
7MSV	NMR	Organic	Nucleotide, berberine	dGMP and berberine	d(AAGGGAGGGCGGCGGGACA)	PDGFR-β	K^+^	Unimolecular	Parallel	G-vacancy filling, and 5′- and 3′-end G-tetrad stacking (1:1:2)	Stacking, hydrogen bonds and electrostatic interactions	[96]
**VEGFoncogene promoter G-quadruplexes**												
6LNZ	NMR	Metal-organic	Pt(II) complex	Pt2	d(CGGGGCGGGCCTTGGGCGGGGT)	VEGF	K^+^	Unimolecular	Parallel	Intercalation between 3′-end G-tetrad and loop/flanking C10/G21 (1:1)	Stacking	[97]
**Viral G-quadruplexes**												
6JJI	X-ray crystallography	Organic	Porphyrin	TMPyP4	r(GGCUCGGCGGCGGA)	IE180	K^+^	Bimolecular	Parallel	Intercalation between 3′-end G-tetrad and AA-coupling plane (1:1)	Stacking	[98]
6JJH	X-ray crystallography	Organic	Porphyrin	TMPyP4	r(GGCUCGGCGGCGGA)	IE180	K^+^	Bimolecular	Parallel	Intercalation between 3′-end G-tetrad and AA-coupling plane, and stacking between a pair of cytocines and a pair of uracils of two G-quadruplex units (1:2)	Stacking	[98]

## Data Availability

Not applicable.

## Supplementary Materials

The following supporting information can be downloaded at: https://www.mdpi.com/article/10.3390/pharmaceutics14112361/s1, Table S1: Chemical structures of the ligands, and structures of the G-quadruplex/small-molecule ligand complexes solved thus far by NMR and X-ray crystallography.
