# Supplementary material for: Insights into the Small Molecule Targeting of Biologically Relevant G-Quadruplexes: An Overview of NMR and Crystal Structures
Source: Pharmaceutics. 2022 Nov 1;14(11):2361. doi: 10.3390/pharmaceutics14112361 (PMC9696056; doi:10.3390/pharmaceutics14112361)
Supplement: Supplementary file 1 [file pharmaceutics-14-02361-s001.zip › pharmaceutics-1956249-supplementary.pdf]

Supplementary Information

to the manuscript:

Insights into the small molecule targeting of biologically relevant G-quadruplexes: an overview of NMR and crystal structures

Andrea Criscuolo[1], Ettore Napolitano[1], Claudia Riccardi[1], Domenica Musumeci[1,2], Chiara Platella[1,*] and Daniela Montesarchio[1]

[1]Department of Chemical Sciences, University of Naples Federico II, Via Cintia 21, 80126 Naples, Italy

[2]Institute of Biostructures and Bioimages, CNR, 80134 Naples, Italy

*Correspondence: chiara.platella@unina.it

**Table S1.** Chemical structures of the ligands, and structures of the G-quadruplex/small-molecule ligand complexes solved thus far by NMR and X-ray crystallography.				
**PDB ID**	**Ligand features:**<br>**Name**<br>**Chemical structure**	**G-quadruplex features:**<br>**Molecularity**<br>**Topology**<br>**Sequence**	**Structure of the G-quadruplex/ligand complex**	**Refs.**
**Telomeric G-quadruplexes**				
**Human telomeric unimolecular antiparallel G-quadruplexes**				
2MCO	$\Lambda\Lambda$-[{Ru(phen)$_2$}$_2$tpphz]$^{4+}$<br>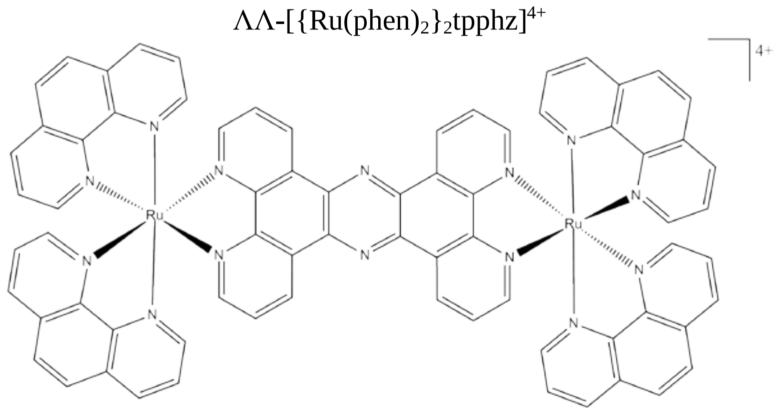	Unimolecular<br>Antiparallel basket<br>d[AGGG(TTAGGG)$_3$]	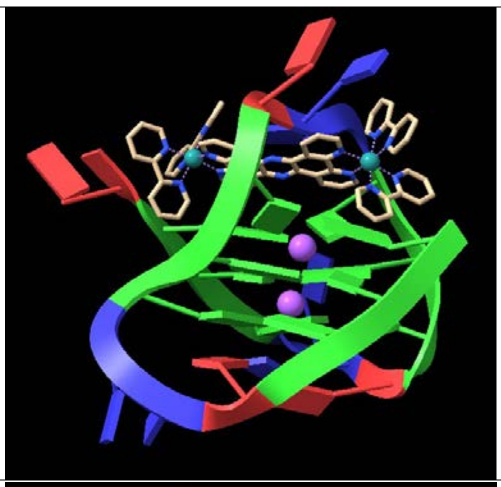	[1]
2MCC	$\Delta\Delta$-[{Ru(phen)$_2$}$_2$tpphz]$^{4+}$<br>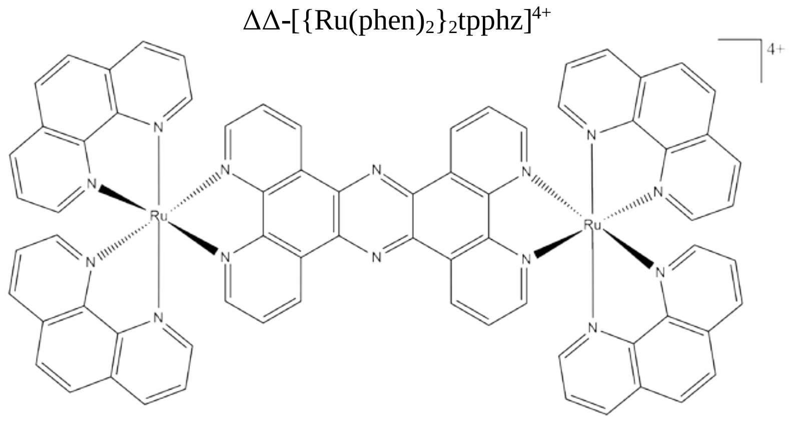	Unimolecular<br>Antiparallel basket<br>d[AGGG(TTAGGG)$_3$]	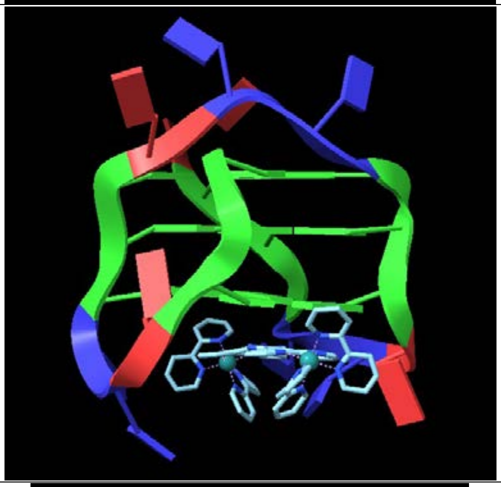	[1]
7OTB	Λ-[Ru(phen)$_2$(qdppz)]$^{2+}$<br>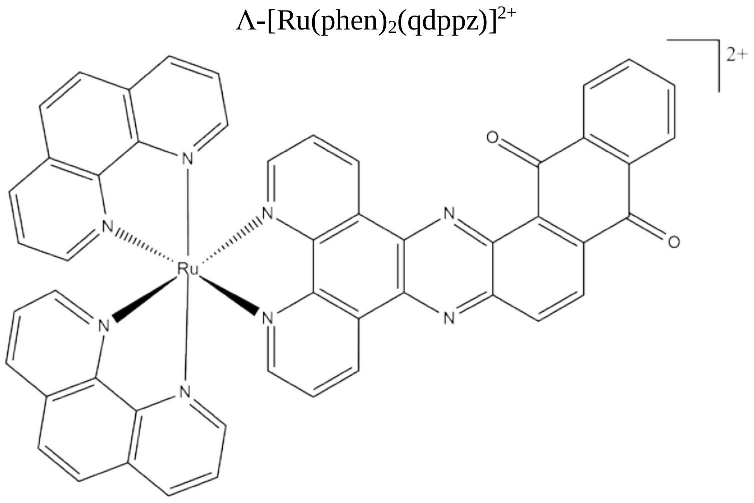	Unimolecular<br>Antiparallel chair<br>d[GGG(TTAGGG)$_2$TTTGGG]	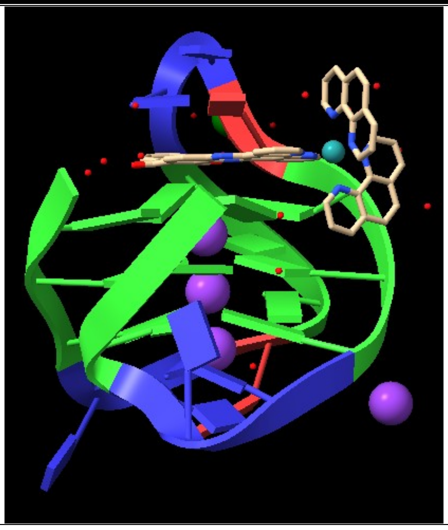	[2]
**Human telomeric unimolecular hybrid G-quadruplexes**				
6CCW	Epiberberine<br>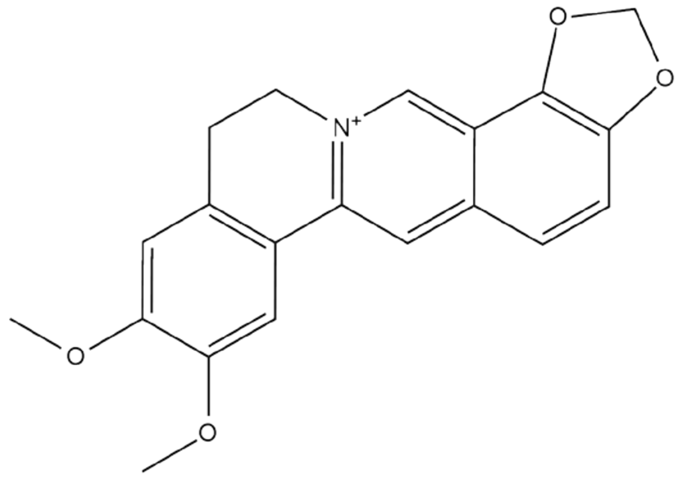	Unimolecular<br>Hybrid-2<br>d[(TTAGGG)$_4$TT]	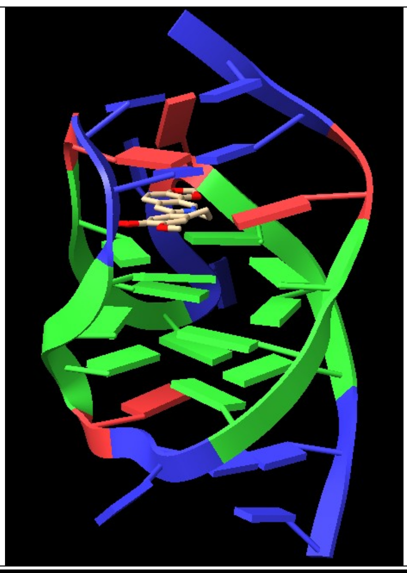	[3]
5MVB	Auoxo6<br>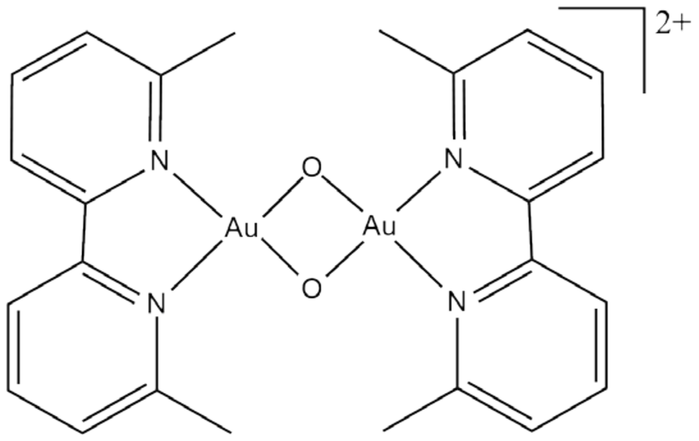	Unimolecular<br>Hybrid-2<br>d[(TTAGGG)$_4$TT]	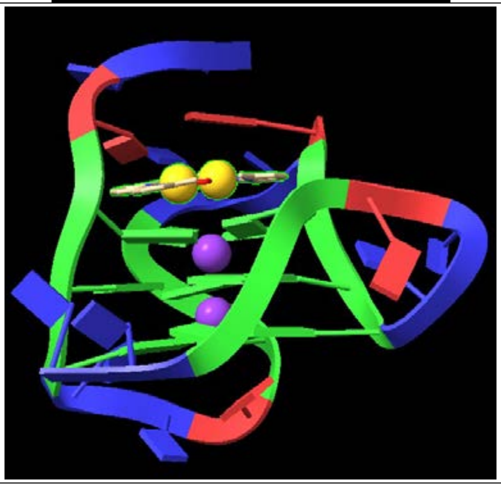	[4]

6KFJ	NBTE<br>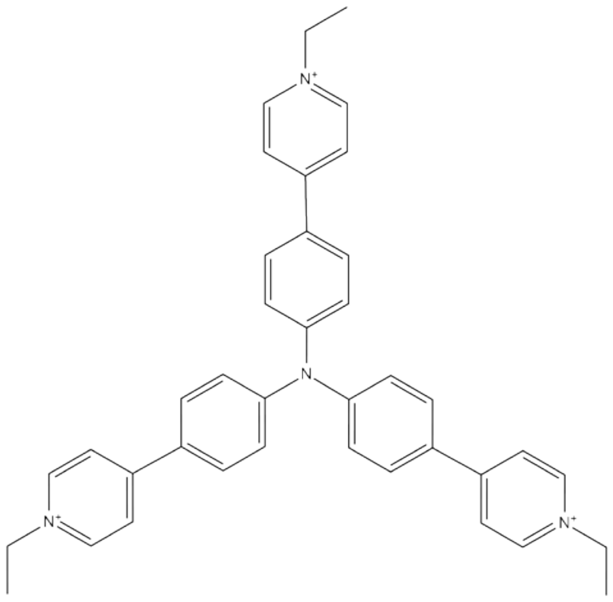	Unimolecular<br>Hybrid-2<br>d[(TTAGGG)$_4$TT]	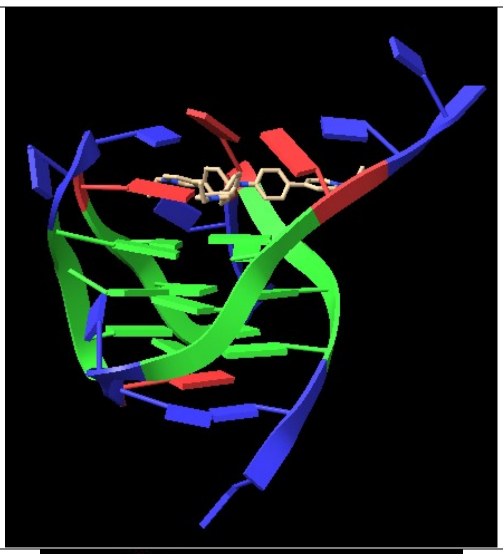	[5]
6KFI		Unimolecular<br>Hybrid-1<br>d[AAAGGG(TTAGGG)$_3$AA]	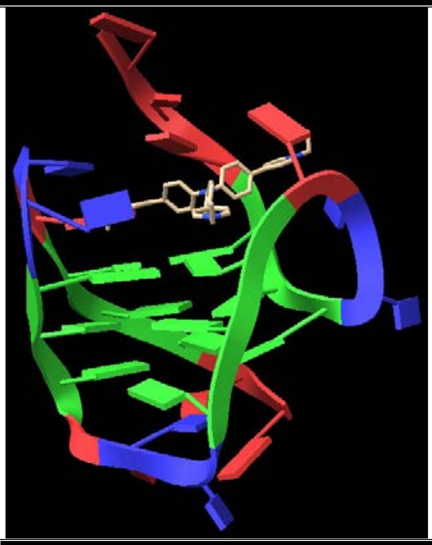	[5]
5Z80	Pt(II)-based tripod<br>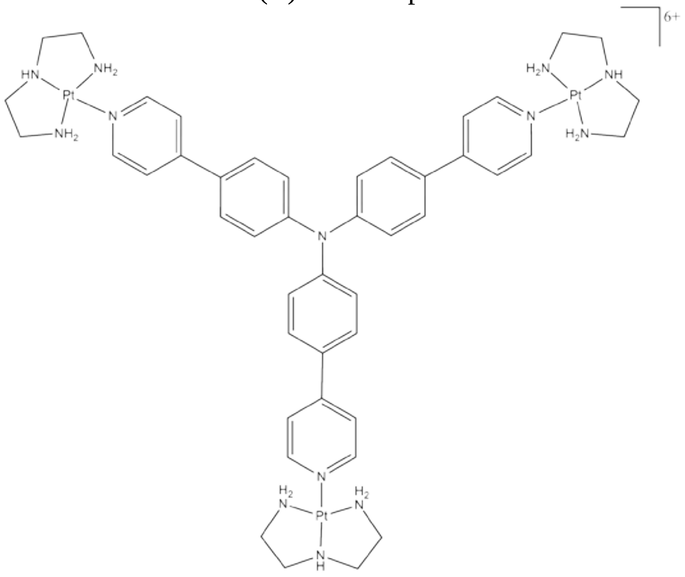	Unimolecular<br>Hybrid-1<br>d[AAAGGG(TTAGGG)$_3$AA]	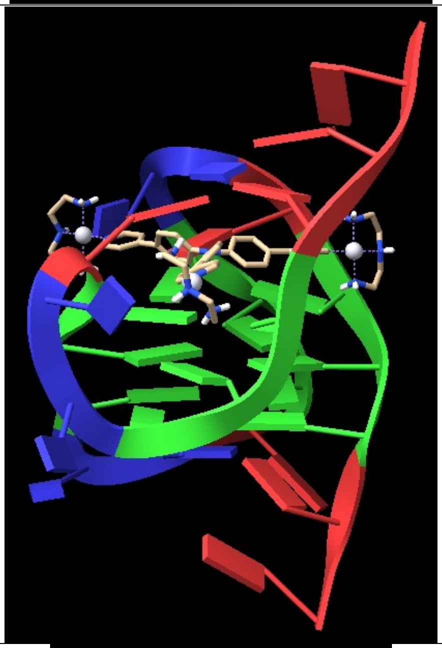	[6]
5Z8F		Unimolecular<br>Hybrid-1<br>d[AAAGGG(TTAGGG)$_3$AA]	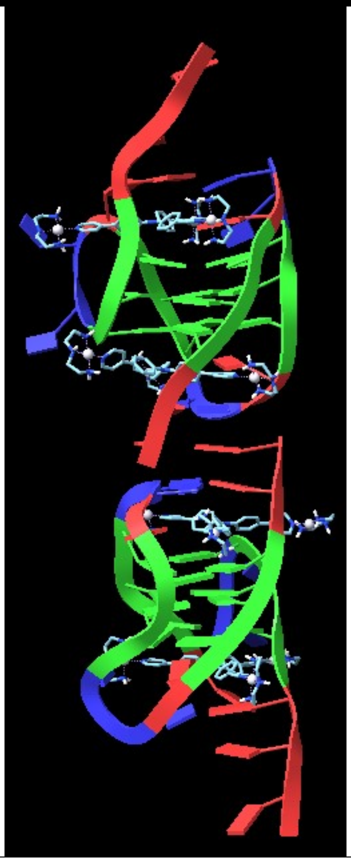	[6]

2MB3	L2H2-6M(2)OTD 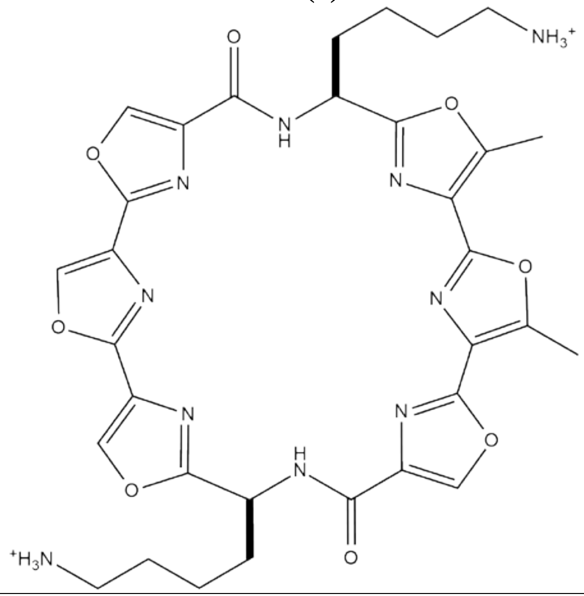	Unimolecular<br>Hybrid-1<br>d[TTGGG(TTAGGG)$_3$A]	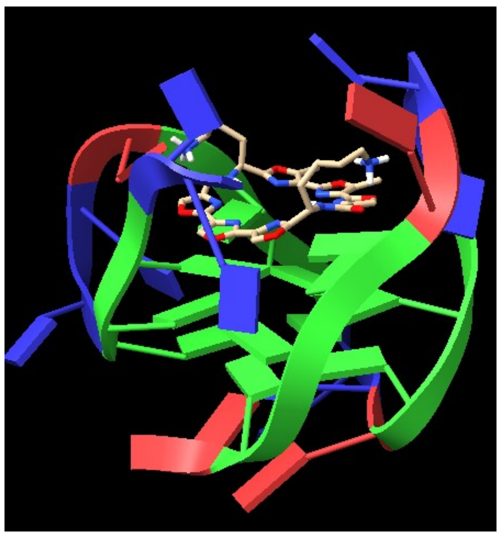	[7]
7Z9L	Phen-DC3 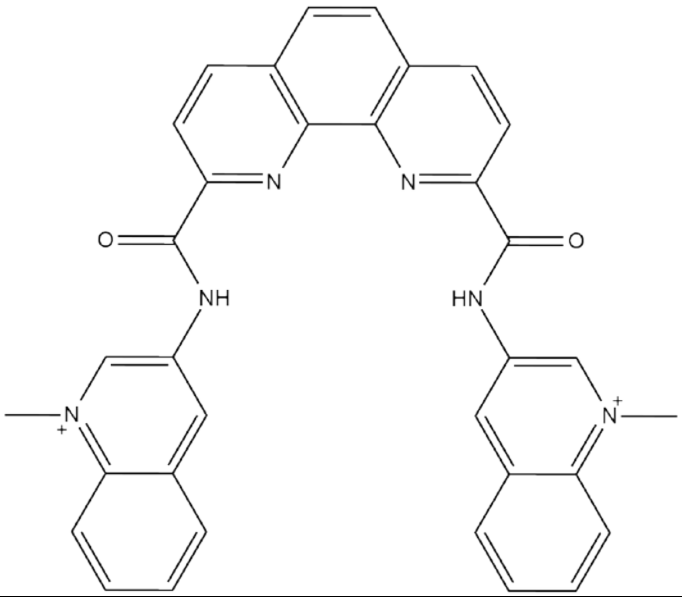	Unimolecular<br>From Hybrid-1 to<br>Antiparallel chair<br>d[TAGGG(TTAGGG)$_3$]	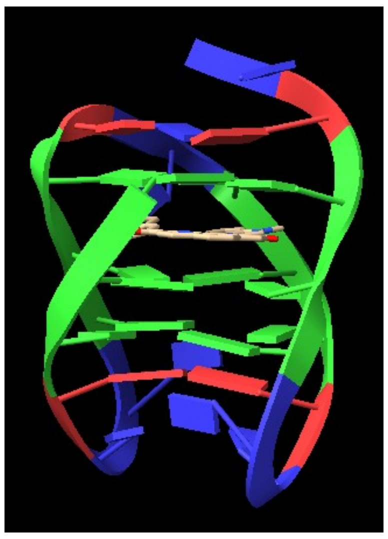	[8]
		**Human telomeric unimolecular parallel G-quadruplexes**		
3R6R	Berberine 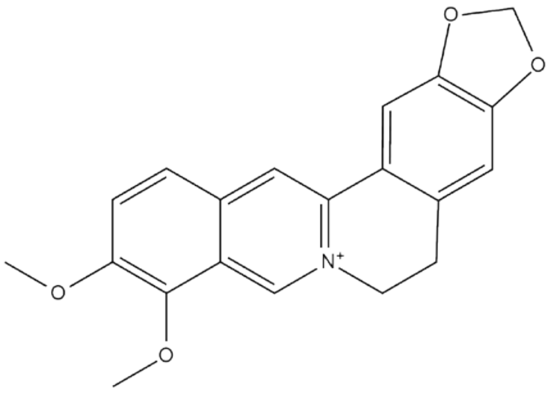	Unimolecular<br>Parallel<br>d[TAGGG(TTAGGG)$_3$]	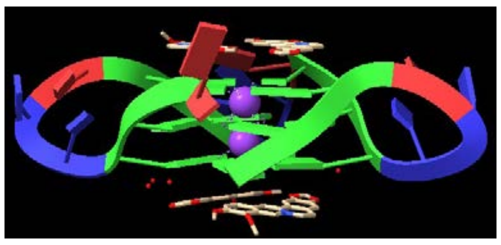	[9]
5CCW	[Au(9-methylcaffein-8-ylidene)$_2$]$^+$ 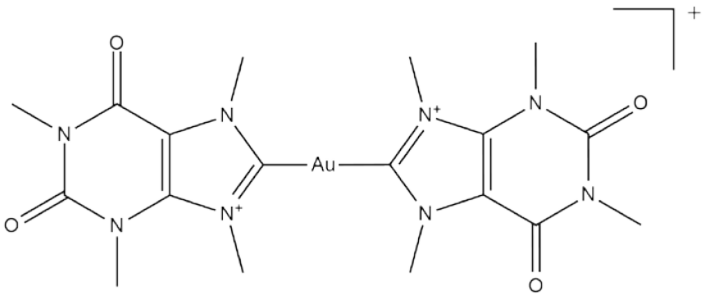	Unimolecular<br>Parallel<br>d[TAGGG(TTAGGG)$_3$]	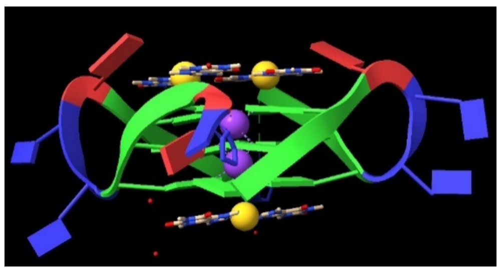	[10]
6H5R	[Au(1-butyl-3-methyl-2-ylidene)$_2$]$^+$ 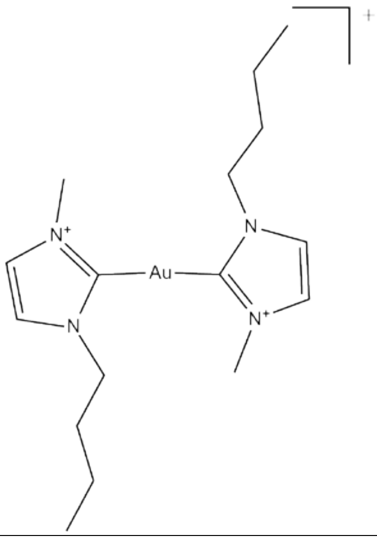	Unimolecular<br>Parallel<br>d[TAGGG(TTAGGG)$_3$T]	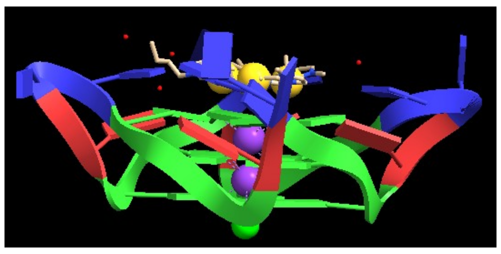	[11]

3CDM	NDI-1 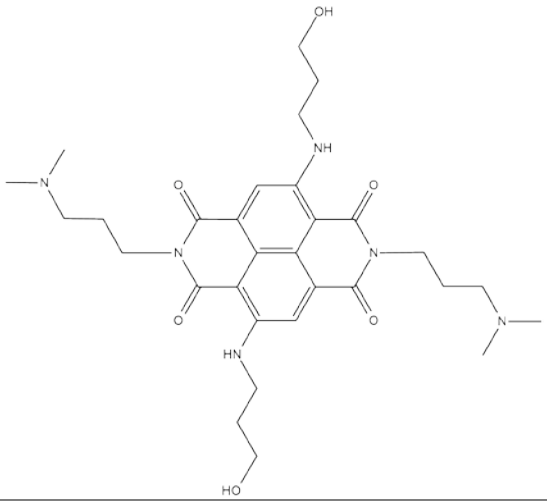	Unimolecular<br>Parallel<br>d[TAGGG(TTAGGG)$_3$]	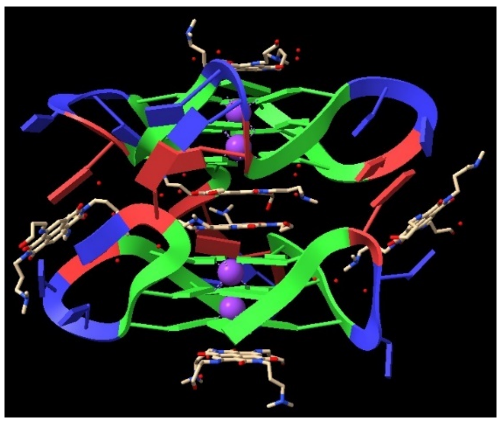	[12]
3SC8	BMSG-SH3 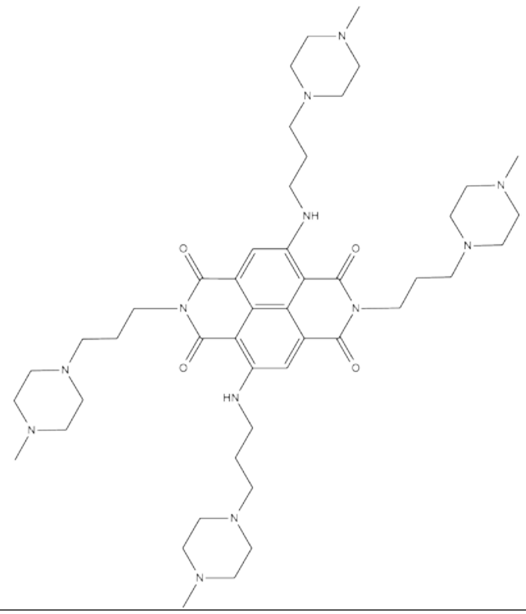	Unimolecular<br>Parallel<br>d[AGGG(TTAGGG)$_3$]	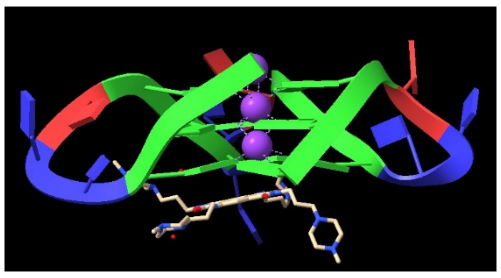	[13]
3T5E	BMSG-SH4 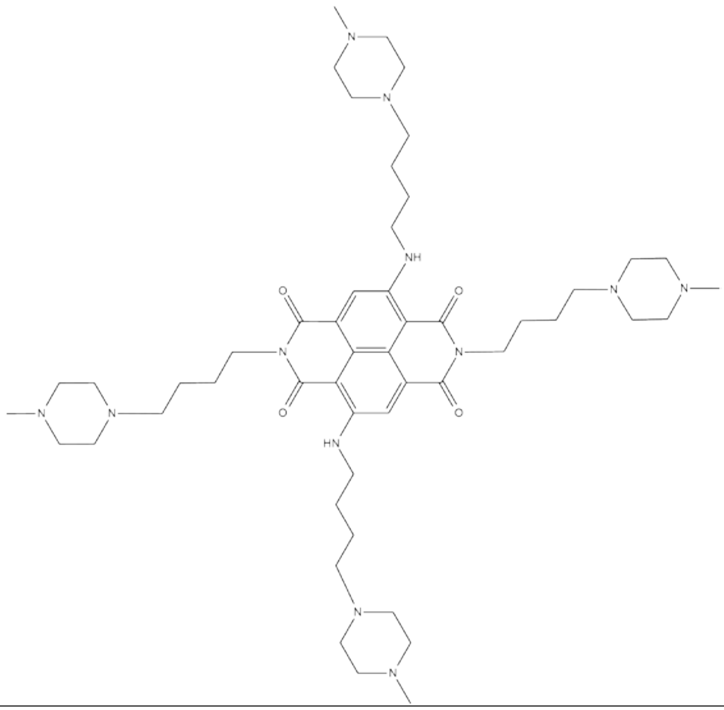	Unimolecular<br>Parallel<br>d[AGGG(TTAGGG)$_3$]	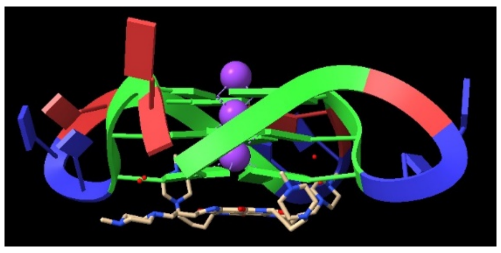	[13]
3UYH	3d 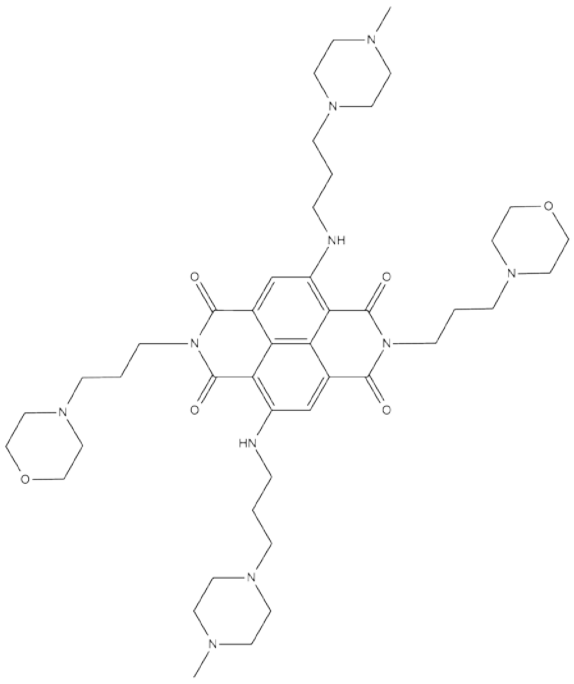	Unimolecular<br>Parallel<br>d[AGGG(TTAGGG)$_3$]	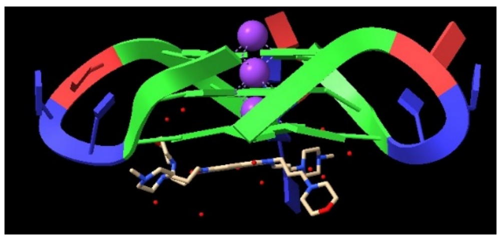	[14]
4DA3		Unimolecular<br>Parallel<br>d[GGG(TTAGGG)$_3$]	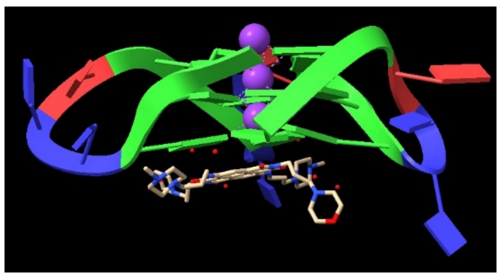	[14]

<table>
<tr><td>4DAQ</td><td>BMSG-SH3
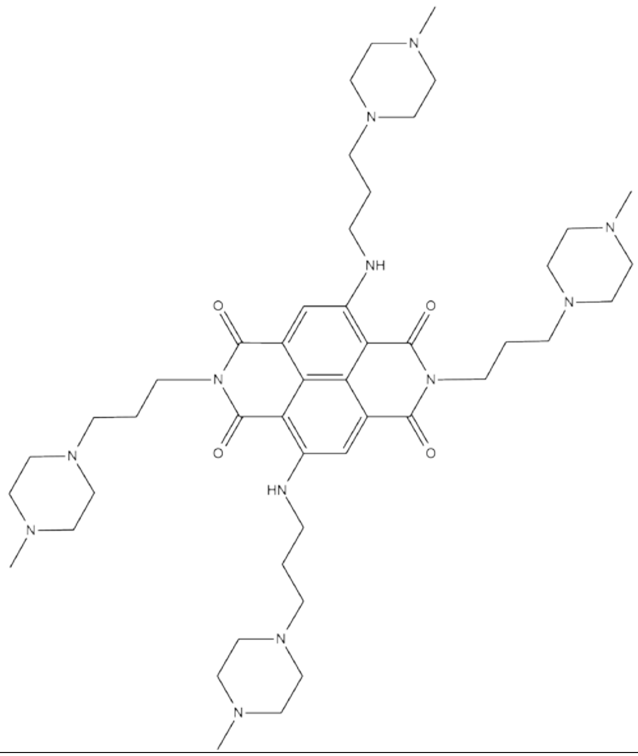</td><td>Unimolecular
Parallel
d[GGG(TTAGGG)$_3$]</td><td>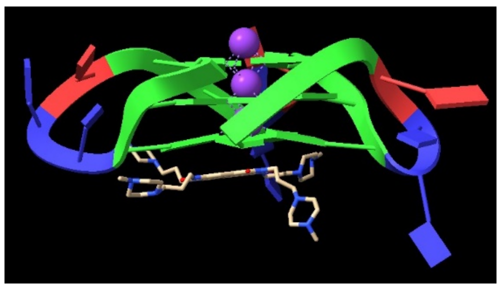</td><td>[14]</td></tr>
<tr><td>4FXM</td><td rowspan="2">N-methyl mesoporphyrin IX
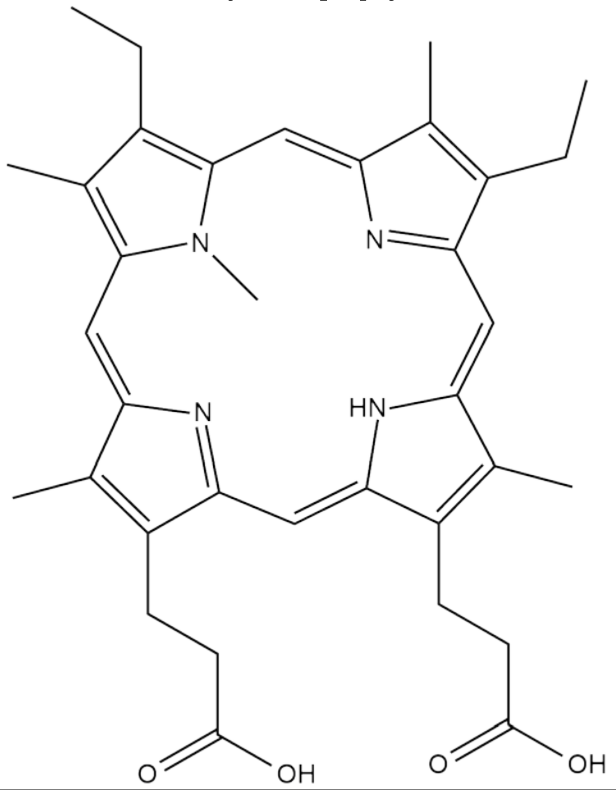</td><td>Unimolecular
Parallel
d[AGGG(TTAGGG)$_3$]</td><td>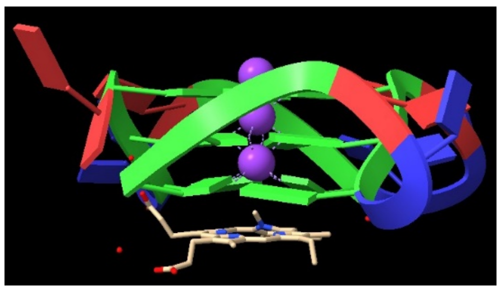</td><td>[15]</td></tr>
<tr><td>4G0F</td><td>Unimolecular
Parallel
d[AGGG(TTAGGG)$_3$]</td><td>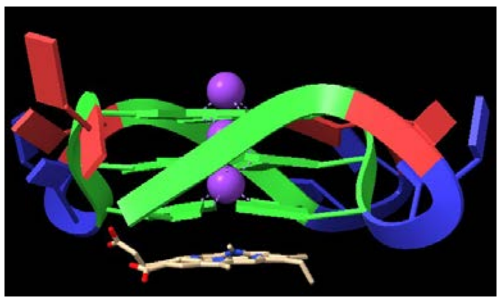</td><td>[15]</td></tr>
<tr><td>6XCL</td><td>Pt(II)-based ligand
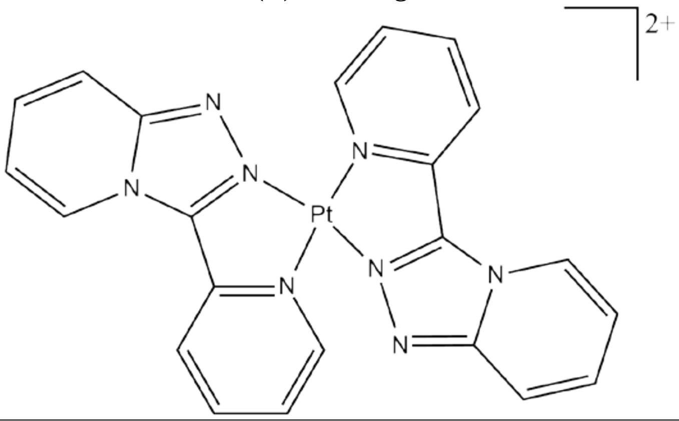</td><td>Unimolecular
Parallel
d[AGGG(TTAGGG)$_3$]</td><td>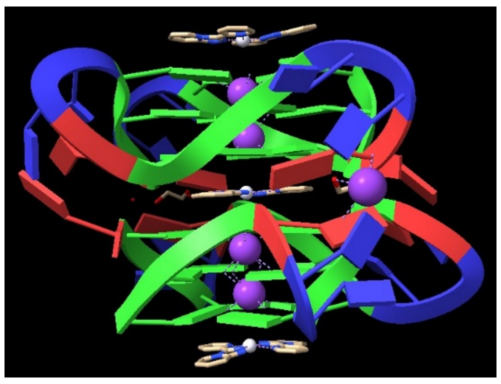</td><td>[16]</td></tr>
<tr><td colspan="5">Human telomeric bimolecular parallel G-quadruplexes</td></tr>
<tr><td>5CDB</td><td>NAX053
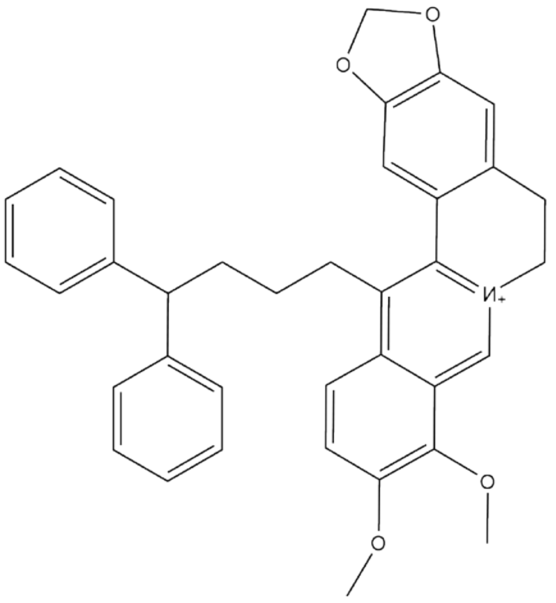</td><td>Bimolecular
Parallel
d(TAGGGTTAGGGT)</td><td>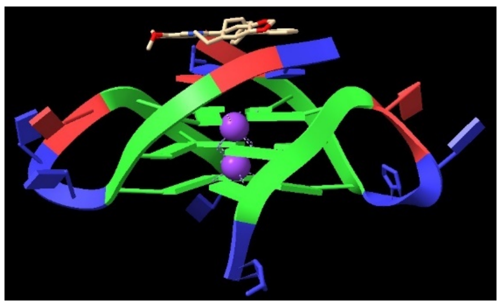</td><td>[17]</td></tr>
</table>

6S15	13-alkylpyridine berberine<br>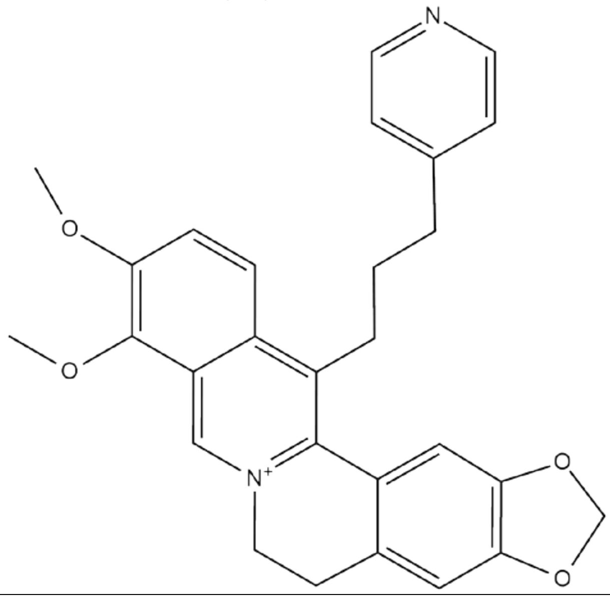	Bimolecular<br>Parallel<br>d(TAGGGTTAGGGT)	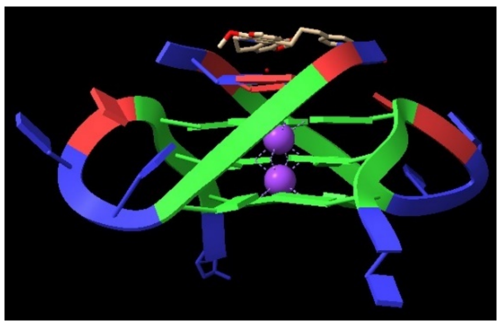	[18]
3CCO	NDI-1<br>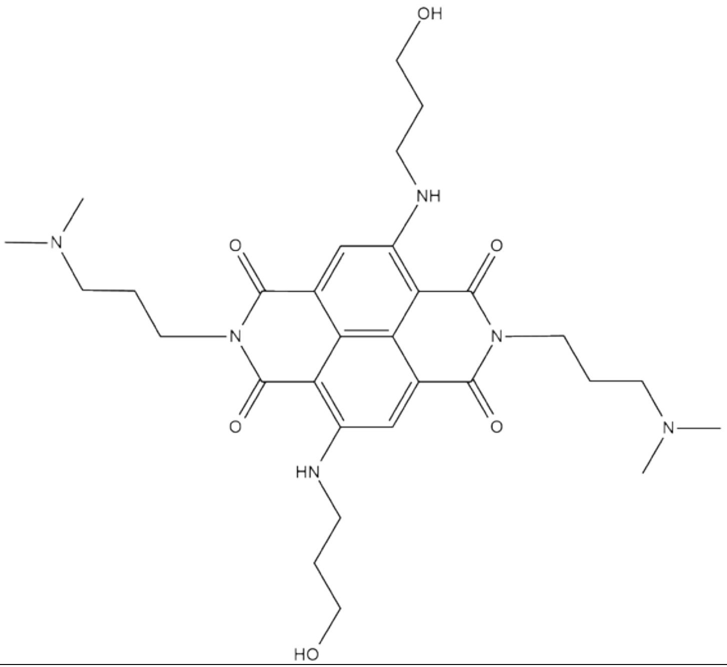	Bimolecular<br>Parallel<br>d(TAGGGTTAGGGT)	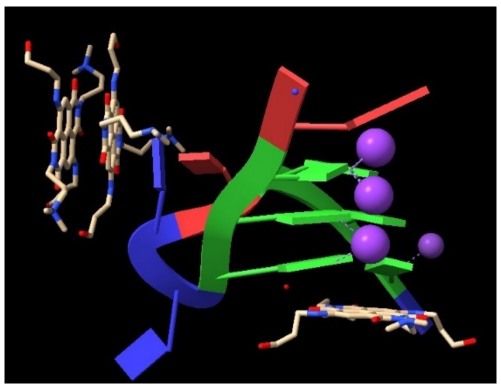	[12]
3CE5	BRACO-19<br>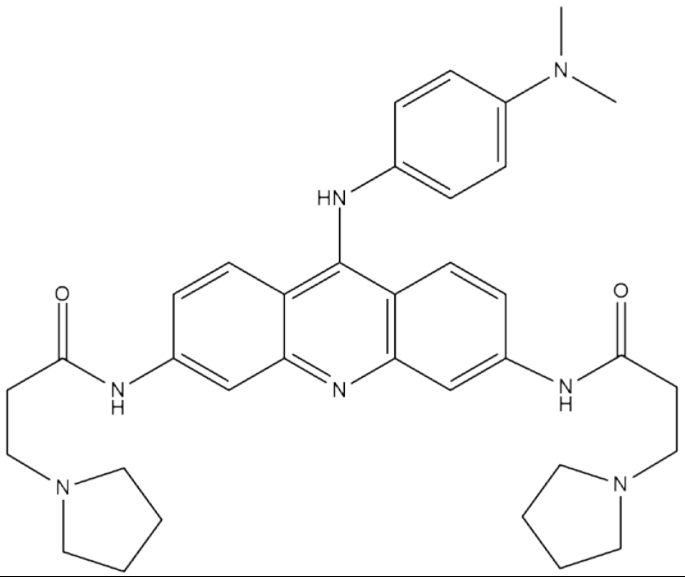	Bimolecular<br>Parallel<br>d(TAGGGTTAGGGT)	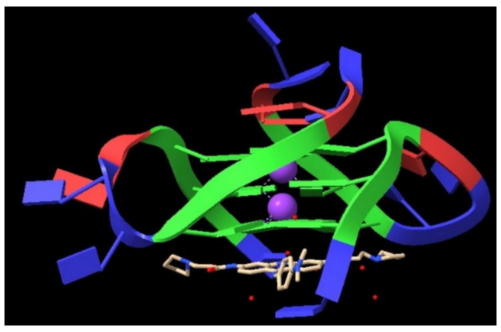	[19]
2HRI	TMPyP4<br>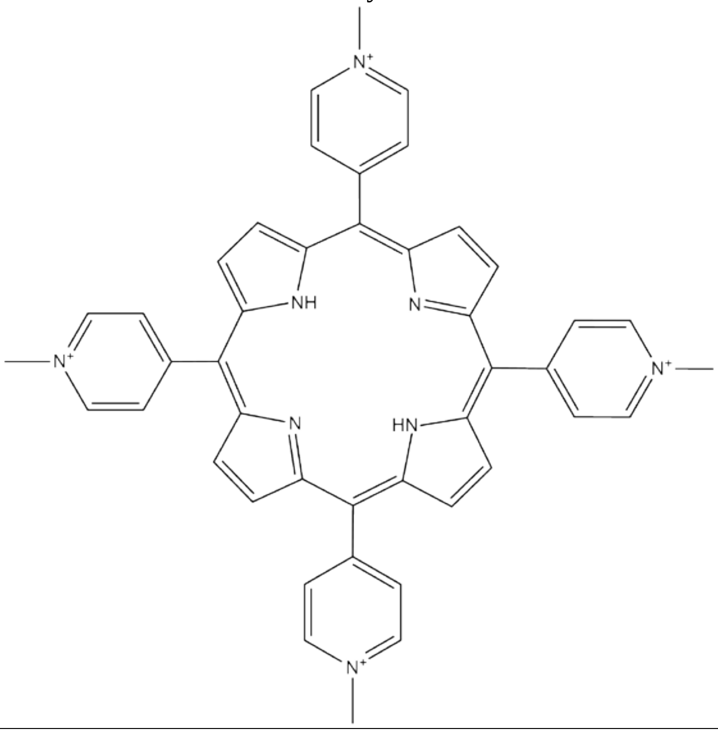	Bimolecular<br>Parallel<br>d(TAGGGTTAGGG)	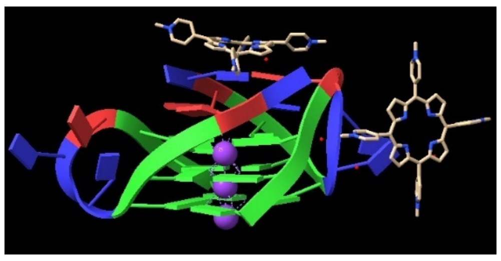	[20]

3QSC	Cu(II) salphen metal complex<br>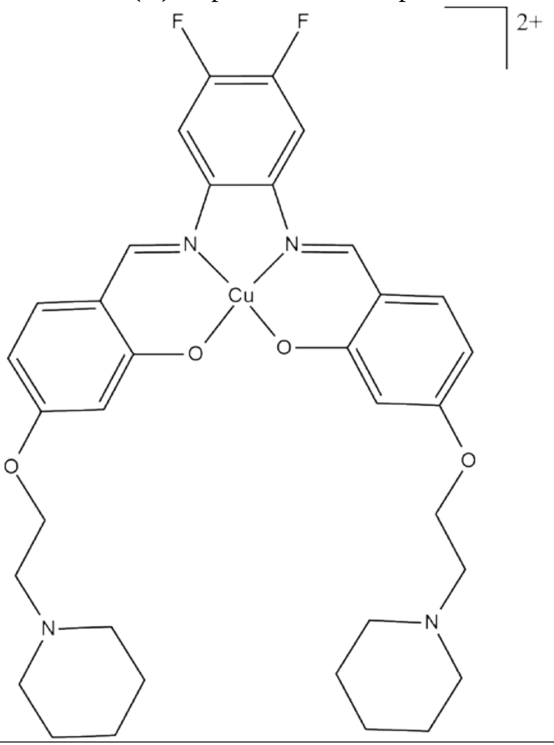	Bimolecular<br>Parallel<br>d(AGGGT^{Br}UAGGTT)	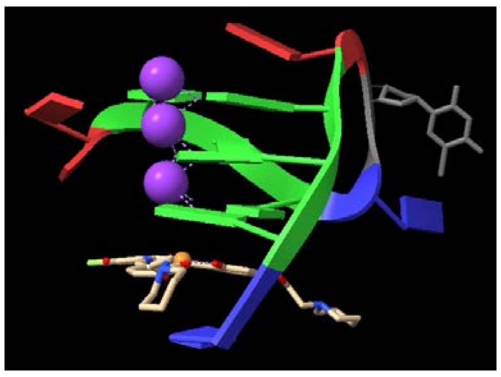	[21]
3QSF	Ni(II) salphen metal complex<br>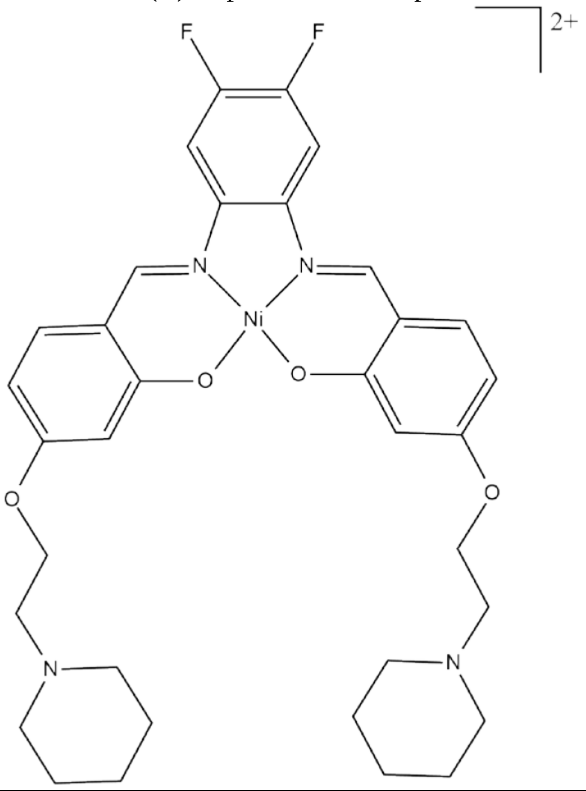	Bimolecular<br>Parallel<br>d(AGGGT^{Br}UAGGTT)	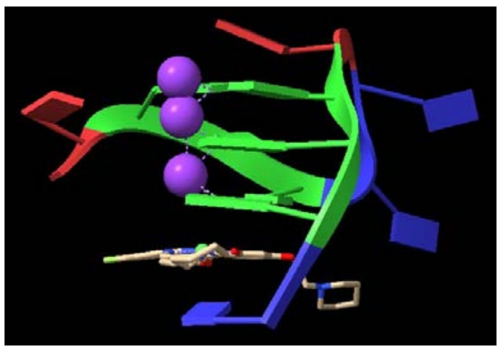	[21]
**Human telomeric tetramolecular parallel G-quadruplexes**				
5LS8	Λ/Δ-[Ru(TAP)$_2$(11-CN-dppz)]$^{2+}$<br>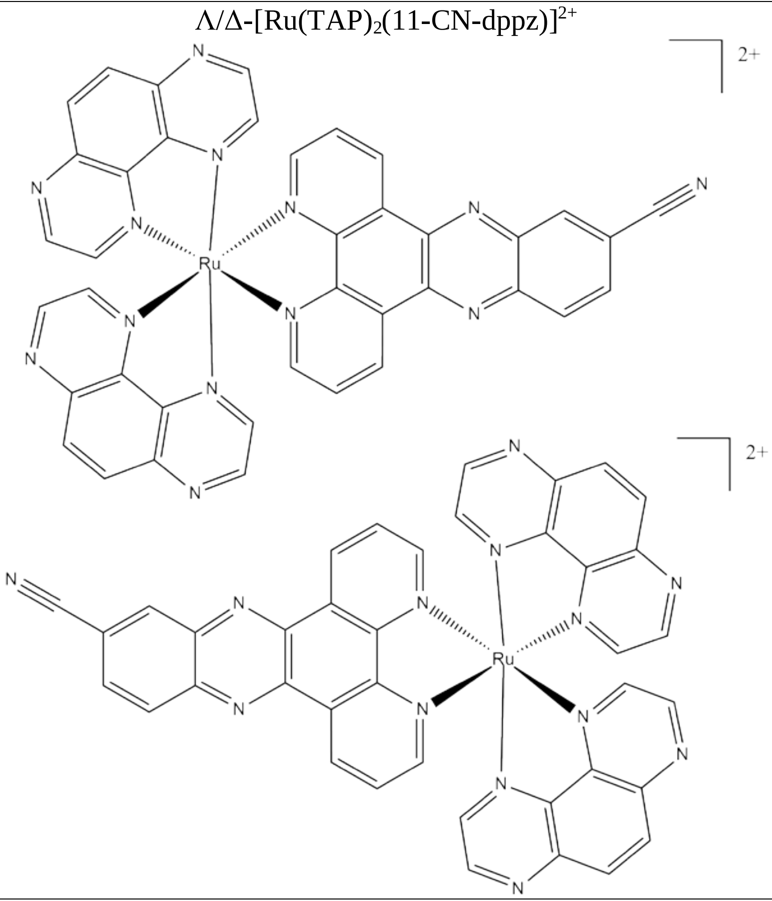	Tetramolecular<br>From Parallel to<br>Antiparallel<br>d(TAGGGTTA)	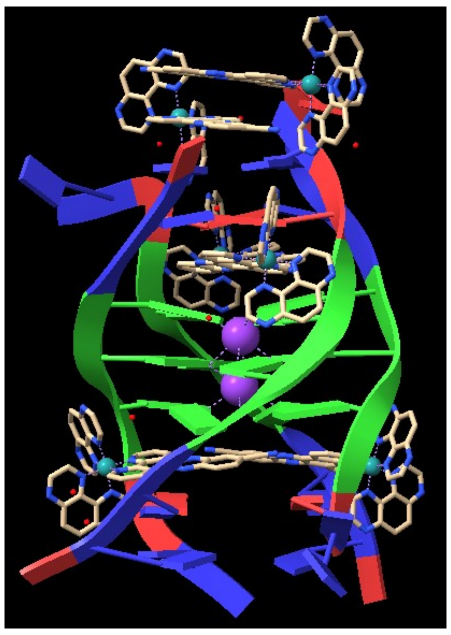	[22]

6RNL	Λ-[Ru(TAP)$_2$(dppz)]$^{2+}$ 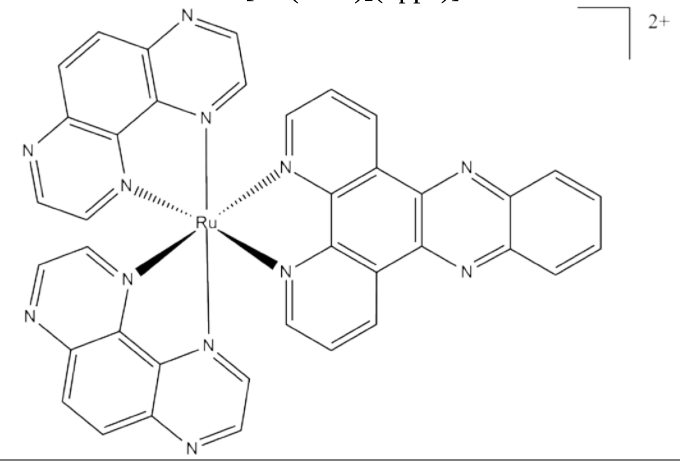	Tetramolecular Parallel d(TAGGGTT)	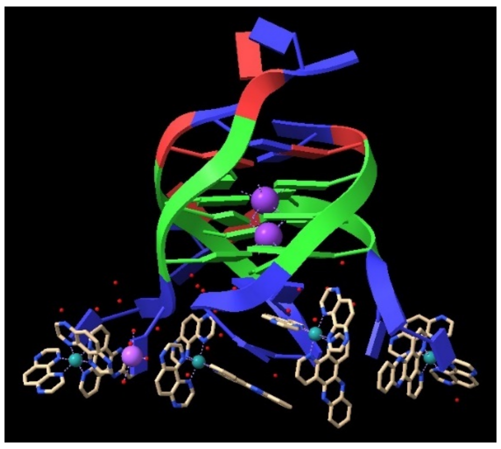	[23]
6KXZ	Epirubicin 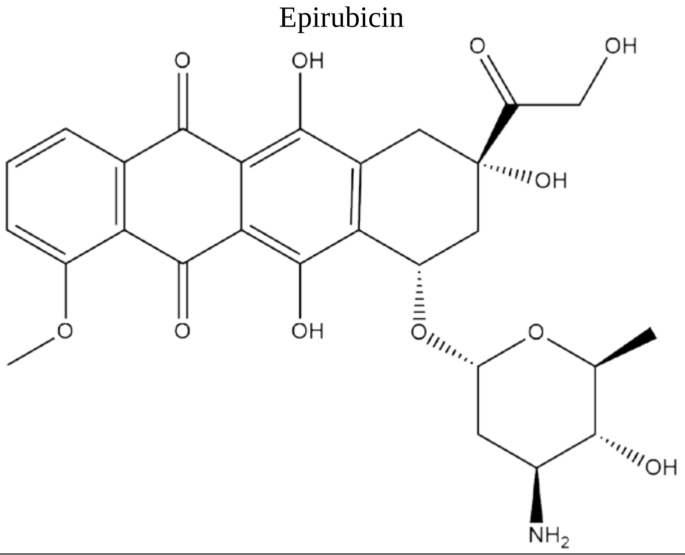	Tetramolecular Parallel d(TTAGGGT)	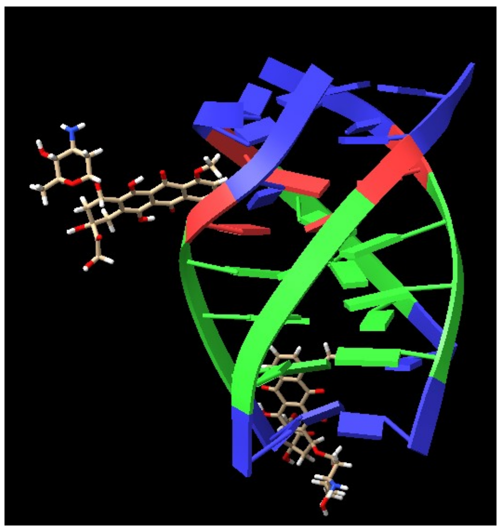	[24]
6KN4	Adriamycin 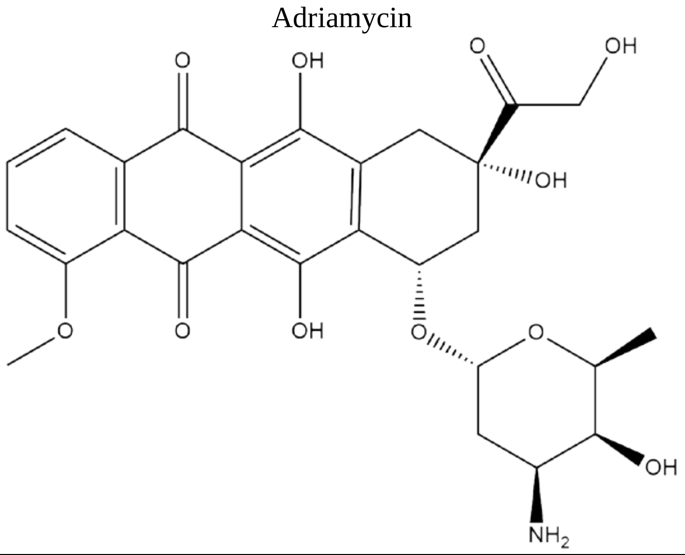	Tetramolecular Parallel d(TTAGGGT)	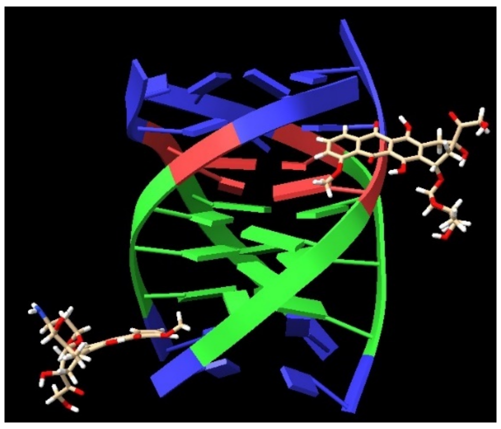	[25]
2MS6	Quercetin 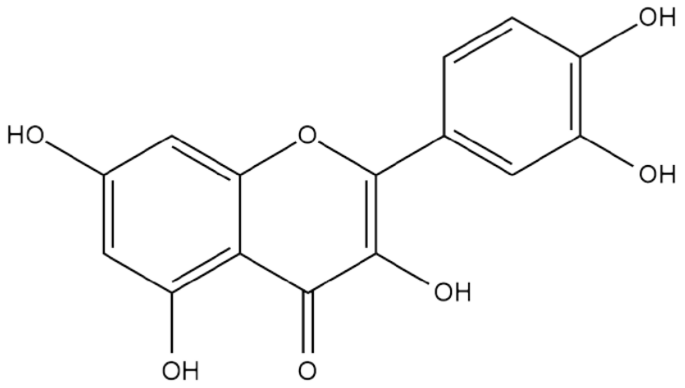	Tetramolecular Parallel d(TTAGGGT)	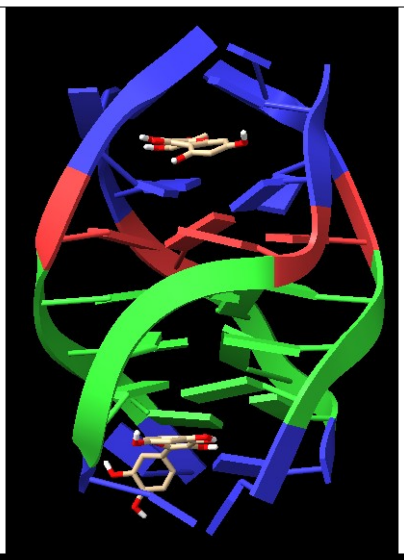	[26]
2JWQ	MMQ$_1$ 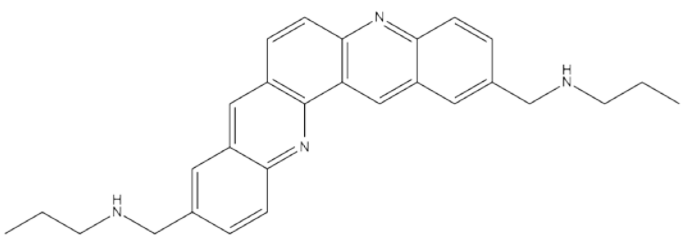	Tetramolecular Parallel d(TTAGGGT)	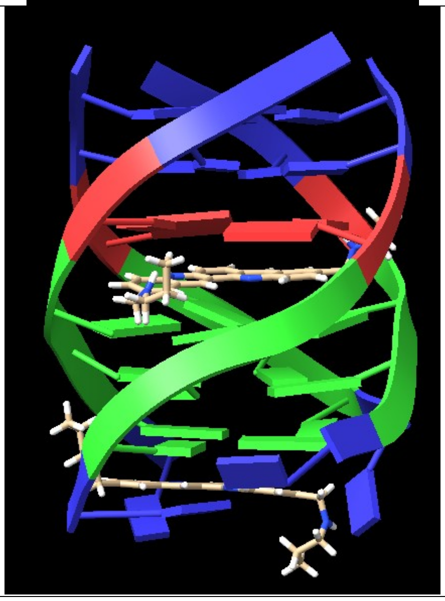	[27]

1NZM	RHPS4<br>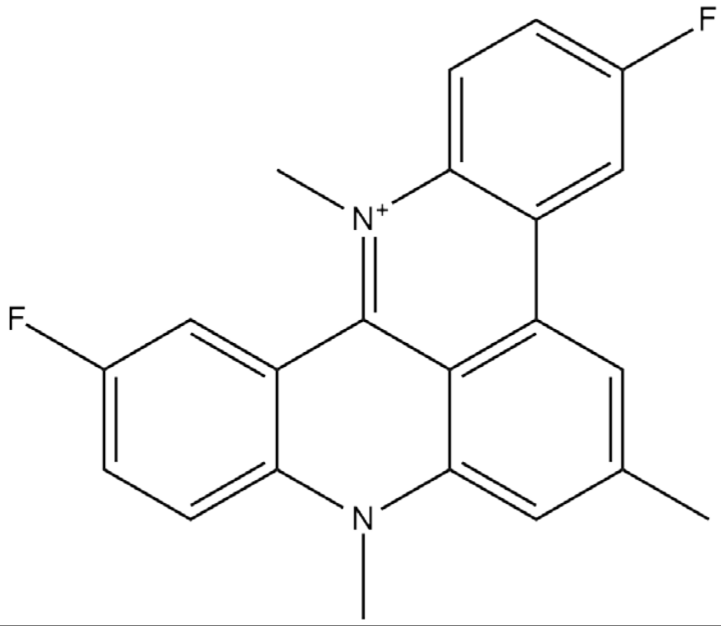	Tetramolecular<br>Parallel<br>d(TTAGGGT)	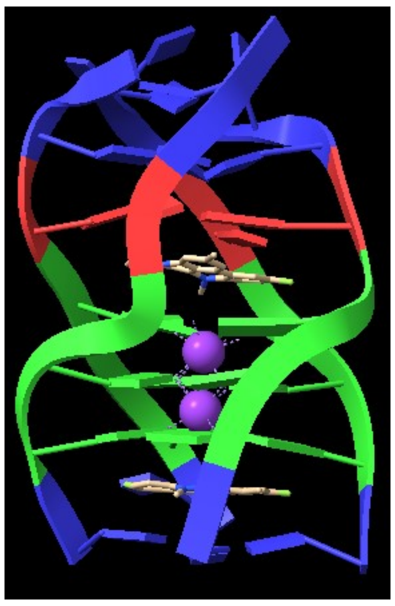	[28]
***Oxytricha* telomeric unimolecular parallel G-quadruplexes**				
6P45	*N*-methylmesoporphyrin IX<br>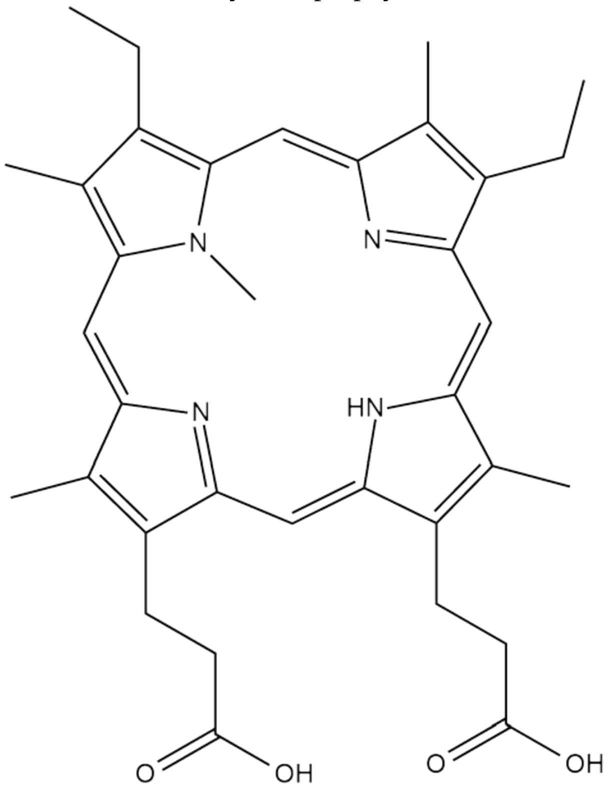	Unimolecular<br>Parallel<br>d[(TGGGT)$_4$]	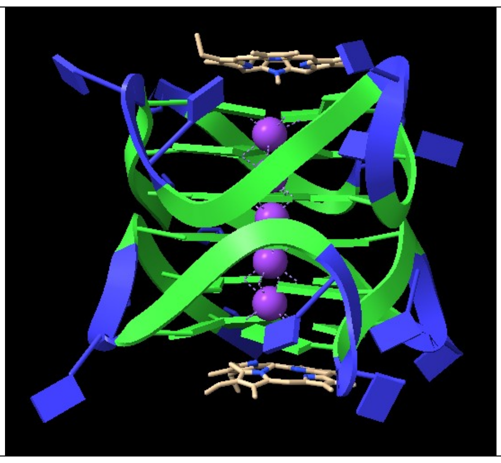	[29]
6PNK		Unimolecular<br>Parallel<br>d[(GGGTT)$_3$GGG]	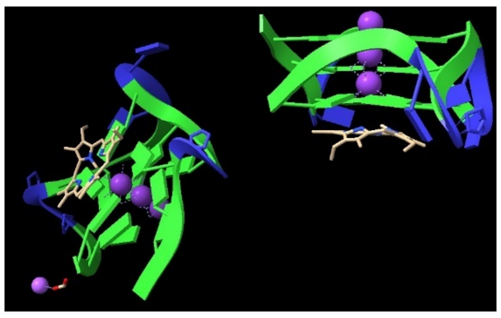	[29]
6K3X	d(AG)<br>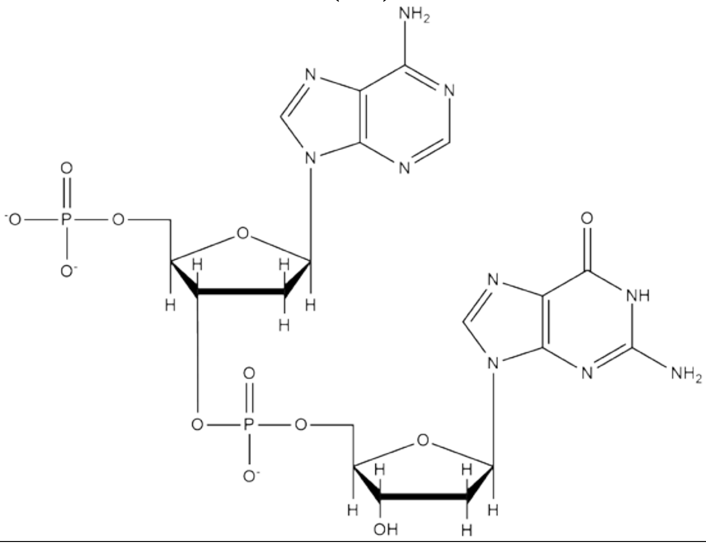	Unimolecular<br>Parallel<br>d[TTGGT(GGGT)$_3$]	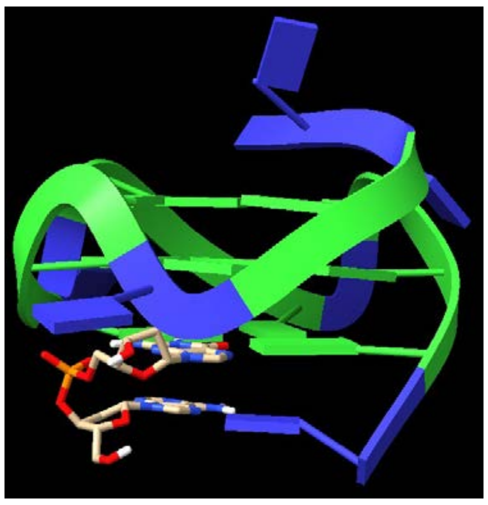	[30]
6K3Y	cGAMP<br>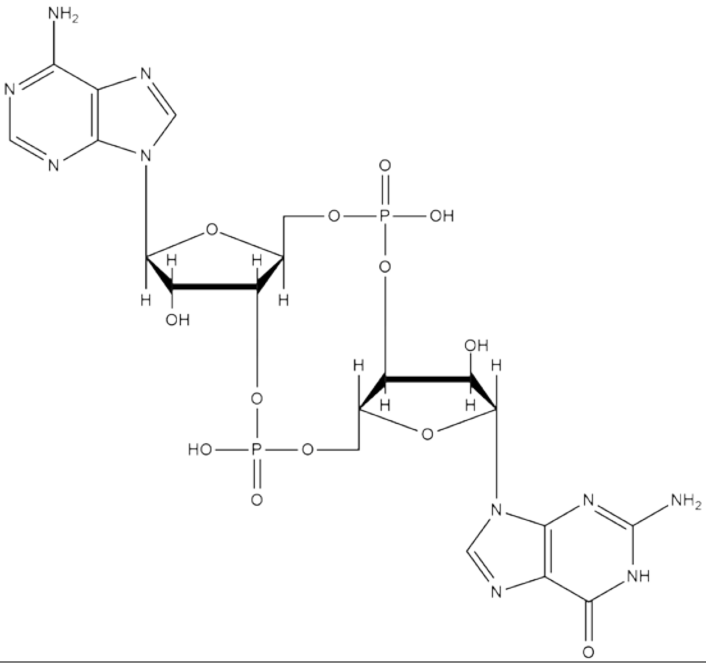	Unimolecular<br>Parallel<br>d[TTGGT(GGGT)$_3$]	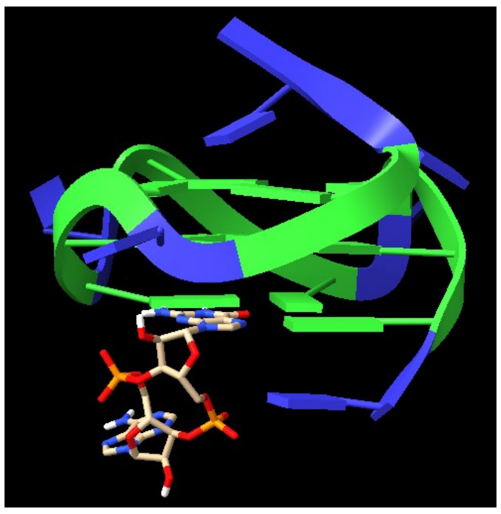	[30]

***Oxytricha* telomeric bimolecular antiparallel G-quadruplexes**				
1L1H	BSU6039<br>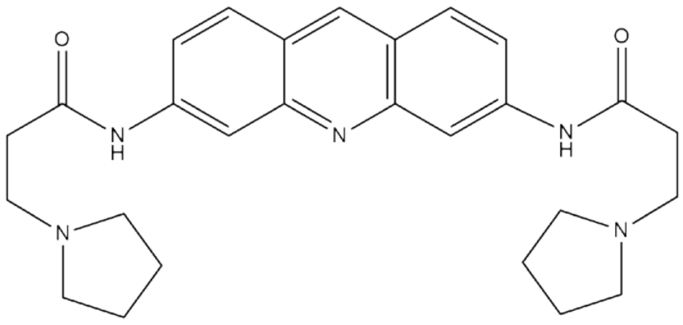	Bimolecular<br>Antiparallel<br>d(GGGGTTTTGGGG)	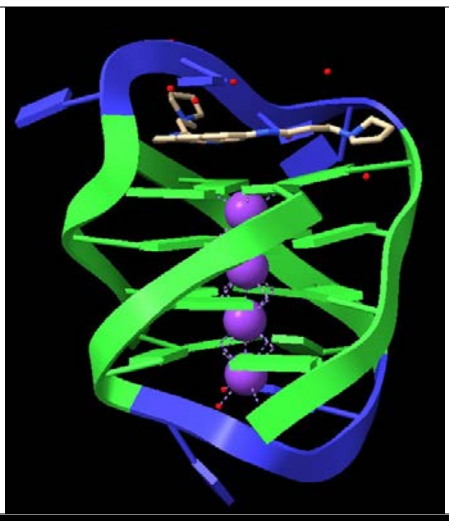	[31]
3EM2	3-morpholinopropionamido 3,6-disubstituted acridine derivative<br>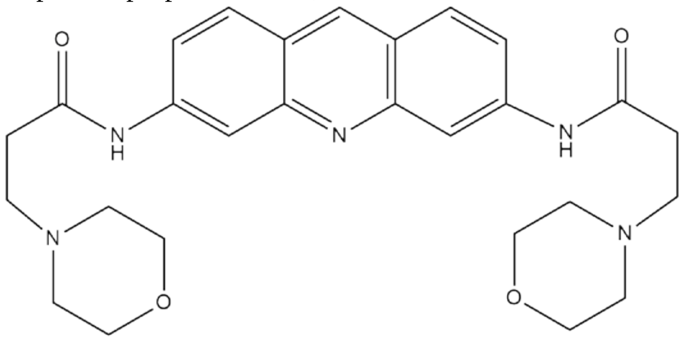	Bimolecular<br>Antiparallel<br>d(GGGGTTTTGGGG)	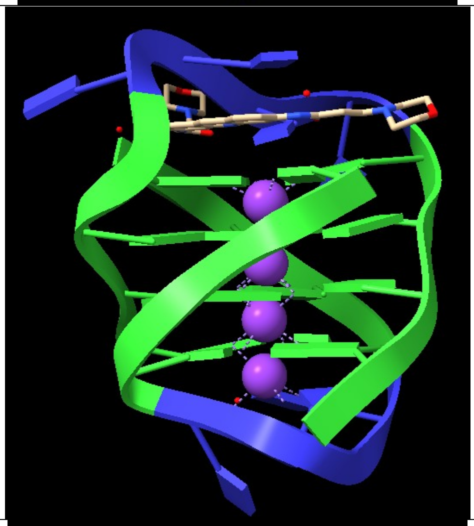	[32]
3EQW	3-(2-ethylpiperidino)propionamido 3,6-disubstituted acridine derivative<br>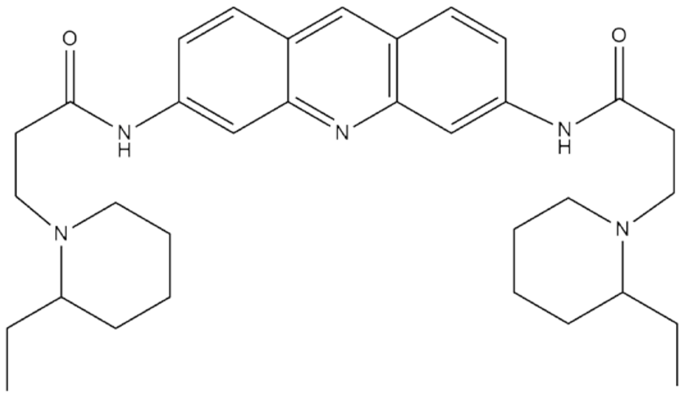	Bimolecular<br>Antiparallel<br>d(GGGGTTTTGGGG)	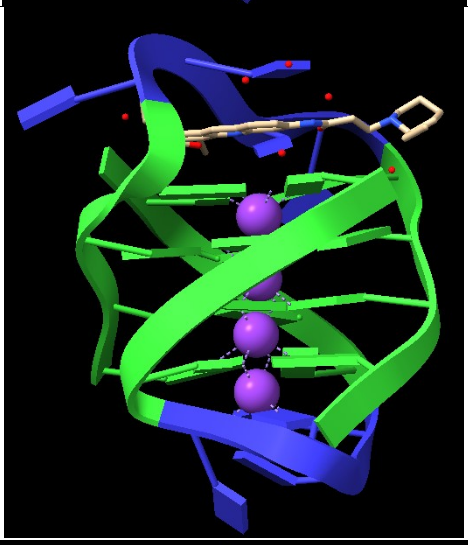	[32]
3EUI		Bimolecular<br>Antiparallel<br>d(GGGGTTTTGGGG)	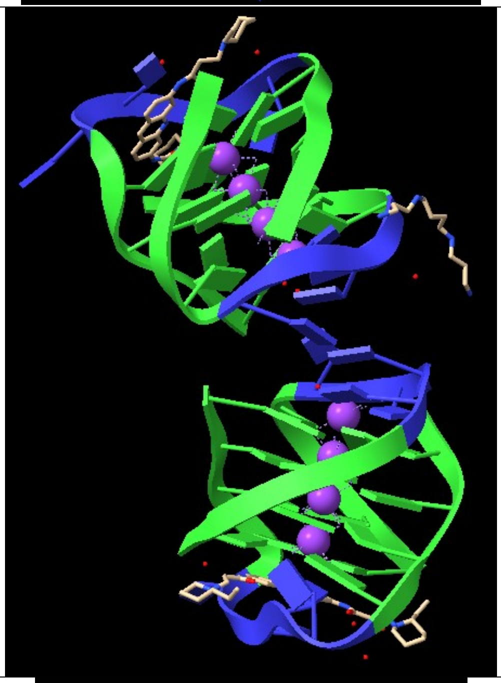	[32]
3ERU	3-(2-methylpiperidino)propionamido 3,6-disubstituted acridine derivative<br>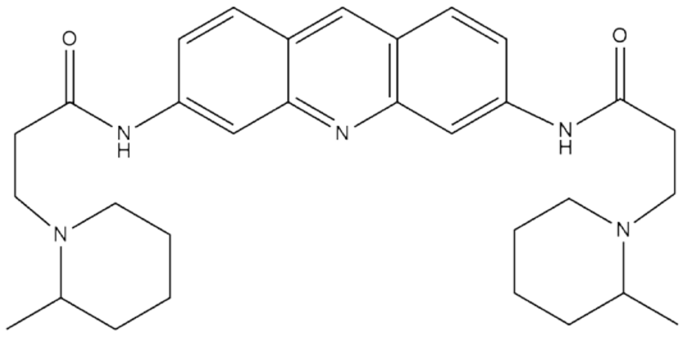	Bimolecular<br>Antiparallel<br>d(GGGGTTTTGGGG)	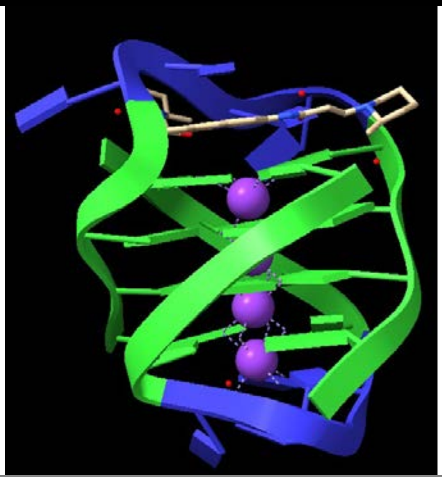	[32]

3ES0	3-(4-methylpiperidino)propionamido 3,6-disubstituted acridine derivative 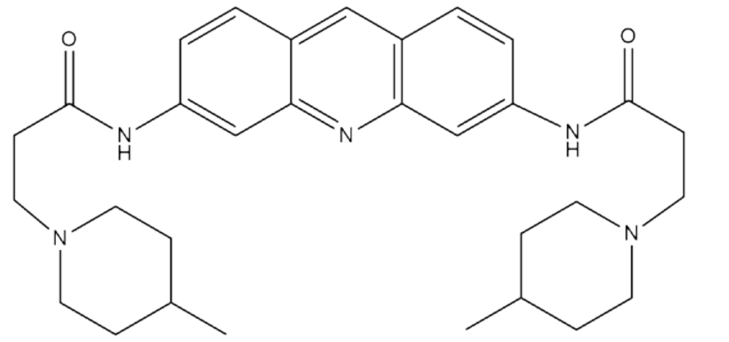	Bimolecular<br>Antiparallel<br>d(GGGGTTTTGGGG)	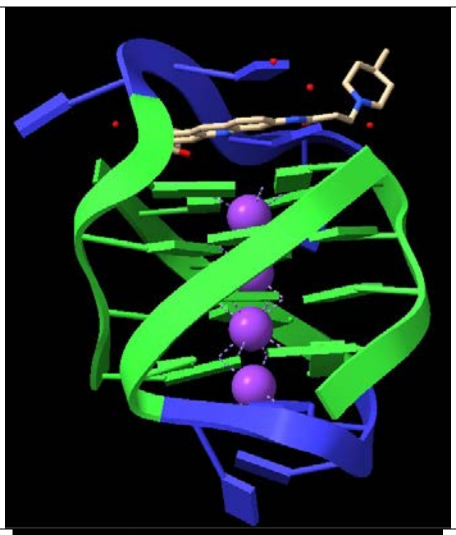	[32]
3ET8	3-(3-methylpiperidino)propionamido groups 3,6-disubstituted acridine derivative 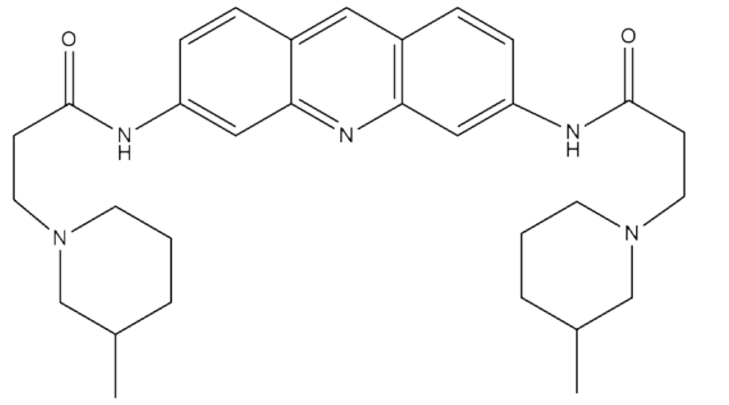	Bimolecular<br>Antiparallel<br>d(GGGGTTTTGGGG)	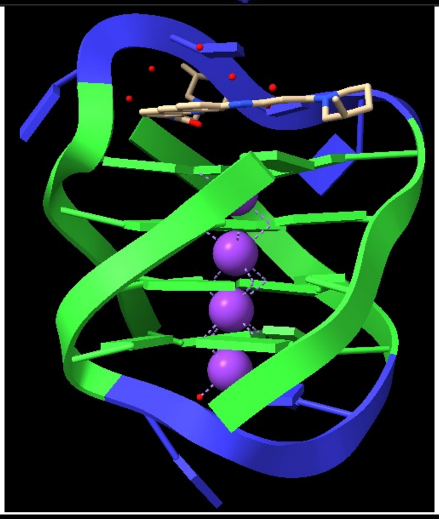	[32]
3EUM	3-(azepan-1-yl)propionamido 3,6-disubstituted acridine derivative 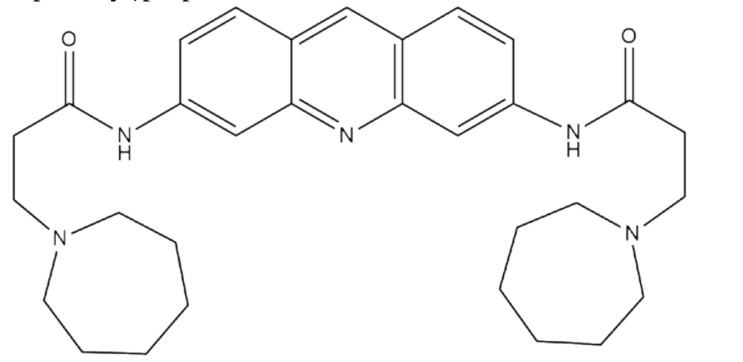	Bimolecular<br>Antiparallel<br>d(GGGGTTTTGGGG)	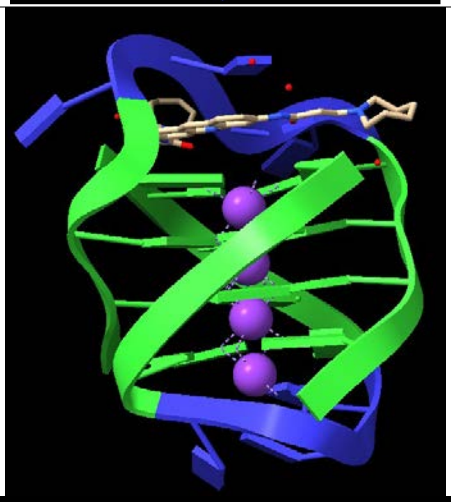	[32]
3NYP	bis-3-fluoropyrrolidine enantiomer (R,R) 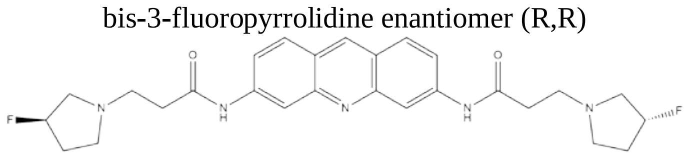	Bimolecular<br>Antiparallel<br>d(GGGGTTTTGGGG)	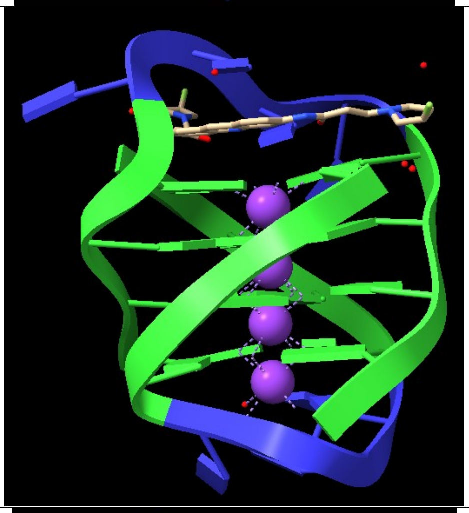	[33]
3NZ7	bis-3-fluoropyrrolidine enantiomer (S,S) 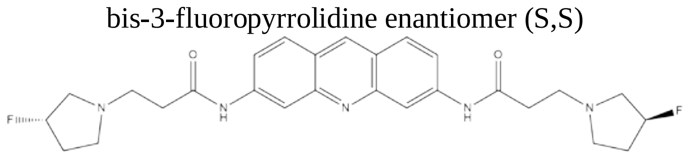	Bimolecular<br>Antiparallel<br>d(GGGGTTTTGGGG)	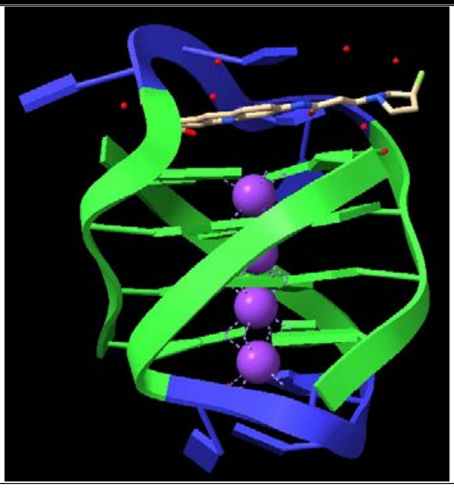	[33]

5HIX	Oligoamide foldamer<br>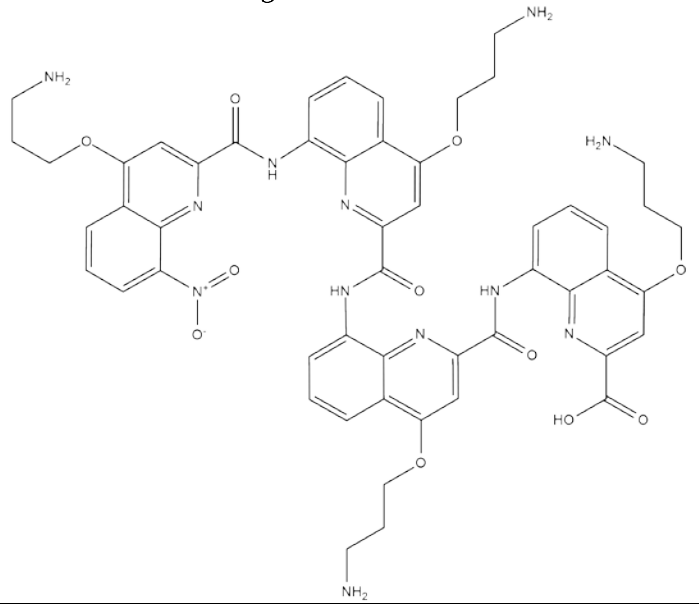	Bimolecular<br>Antiparallel<br>d(GGGGTTTTGGGG)	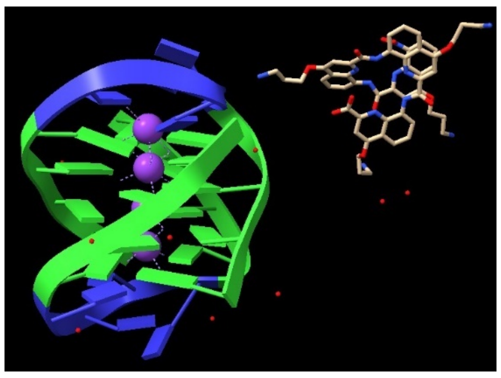	[34]
	Oxytricha **telomeric tetramolecular parallel G-quadruplexes**			
1O0K	Daunomycin<br>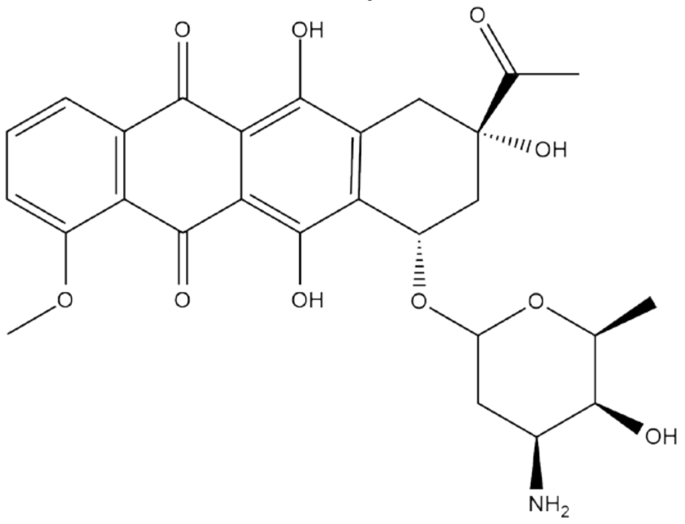	Tetramolecular<br>Parallel<br>d(TGGGGT)	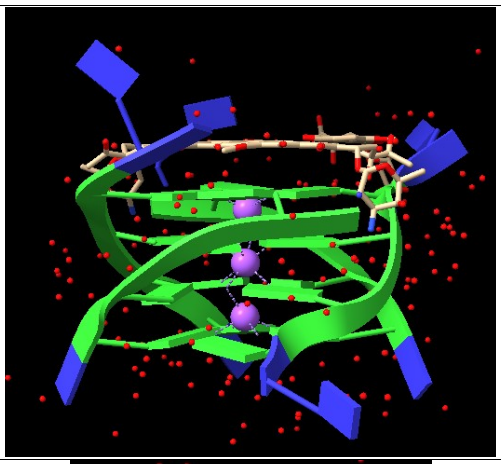	[35]
3TVB		Tetramolecular<br>Parallel<br>d(GGGG)	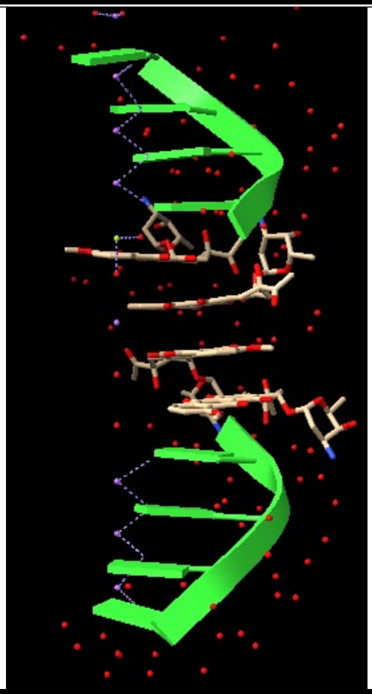	[36]
2JT7	Distamycin<br>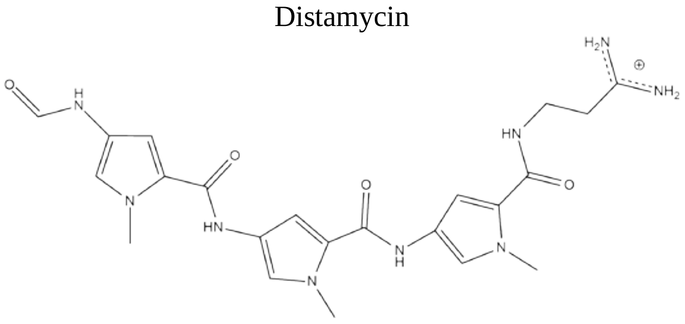	Tetramolecular<br>Parallel<br>d(TGGGGT)	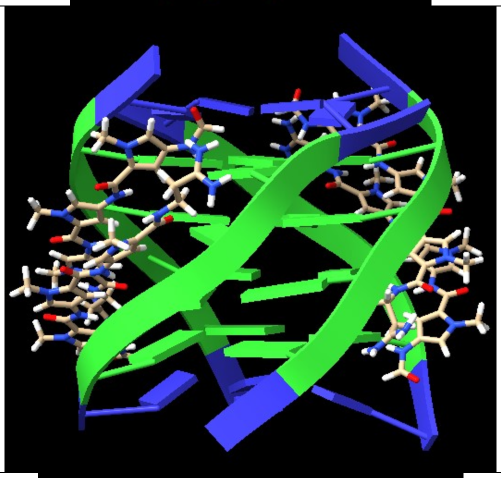	[37]
2KVY	*N*-methyl amide distamycin derivative<br>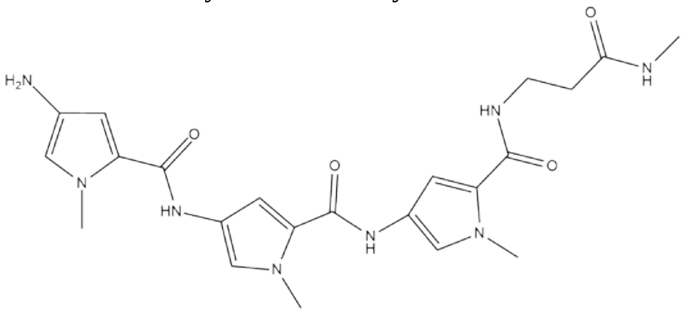	Tetramolecular<br>Parallel<br>d(TGGGGT)	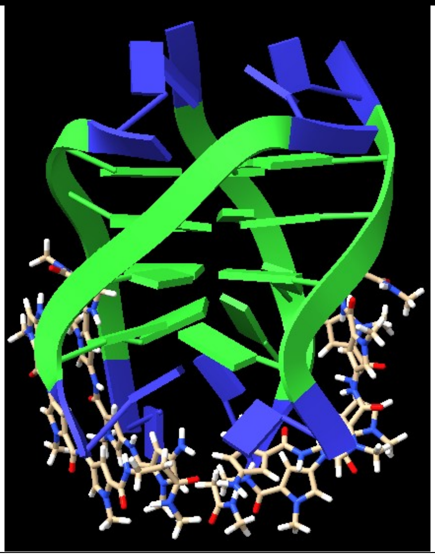	[38]

**RNA telomeric G-quadruplexes**				
3MIJ	Triazole-phenyl-diethylamine 3,6-disubstituted acridine 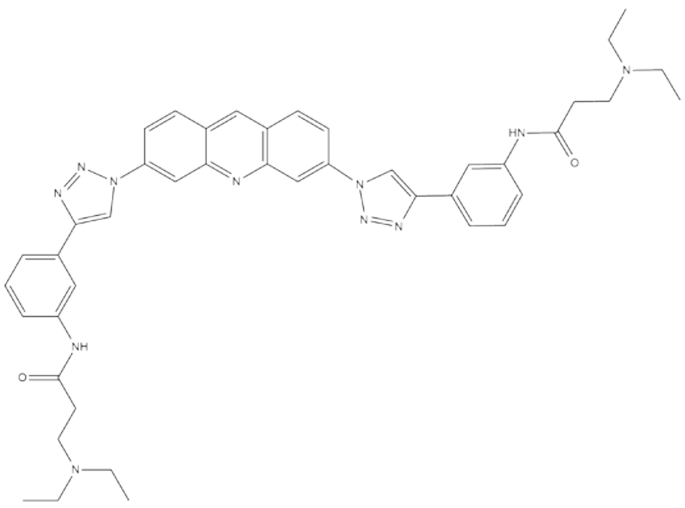	Bimolecular<br>Parallel<br>r[(UAGGGU)$_2$]	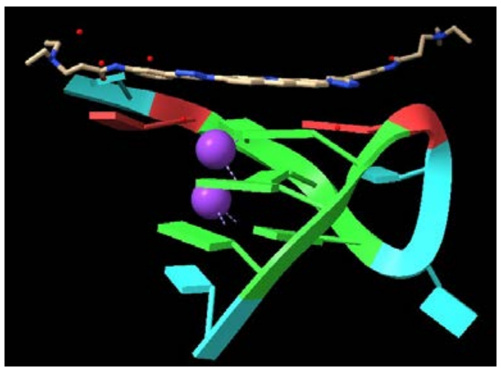	[39]
**Oncogenic G-quadruplexes**				
***C-MYC* oncogene promoter G-quadruplexes**				
2MGN	Phen-DC3 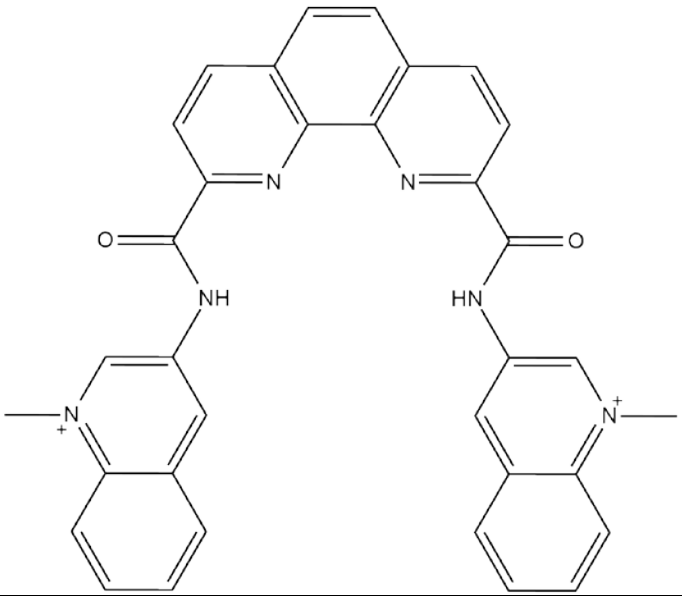	Unimolecular<br>Parallel<br>d(TGAGGGTGGTGAGGGTGGGGAAGG)	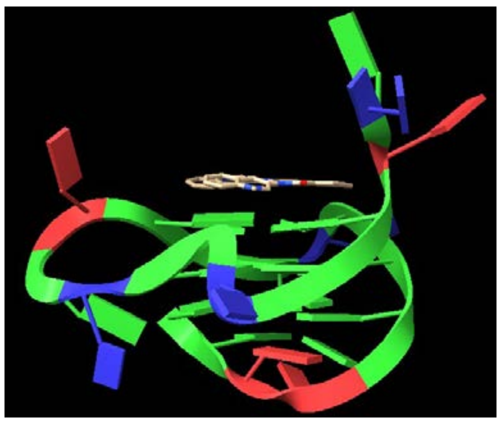	[40]
2A5R	TMPyP4 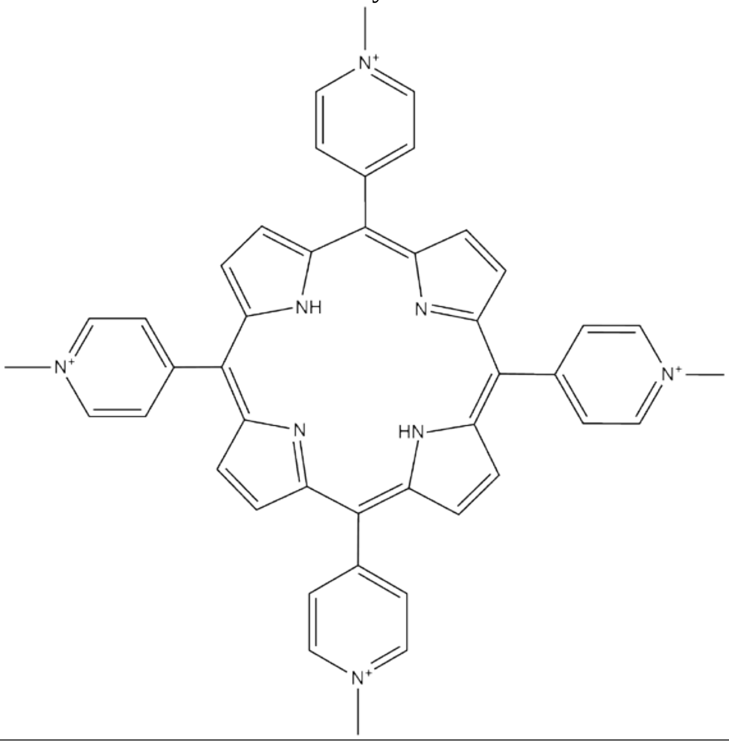	Unimolecular<br>Parallel<br>d(TGAGGGTGGIGAGGGTGGGGAAGG)	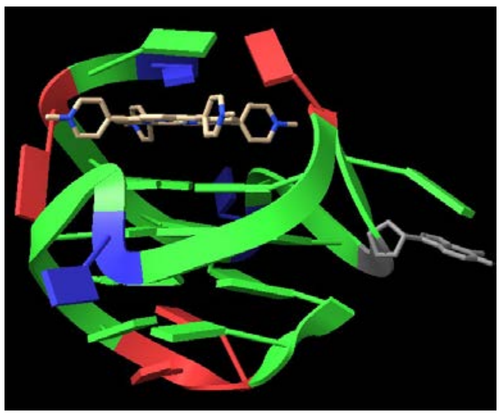	[41]
7N7D	Berberine 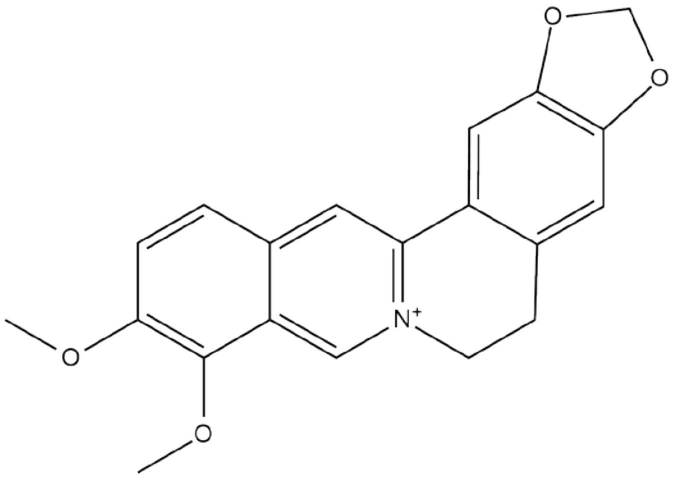	Unimolecular<br>Parallel<br>d(TGAGGGTGGGTAGGGTGGGGAA)	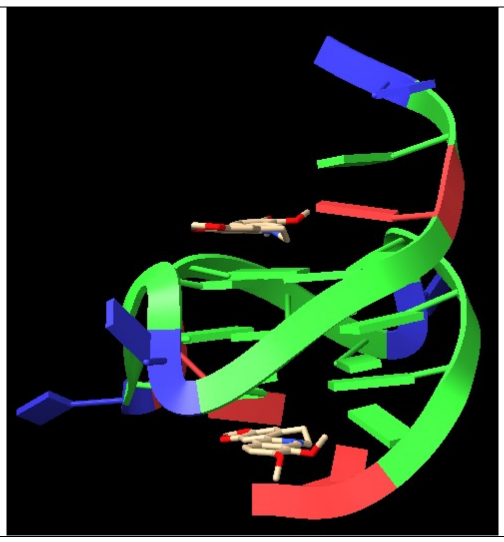	[42]

7N7E	Berberine 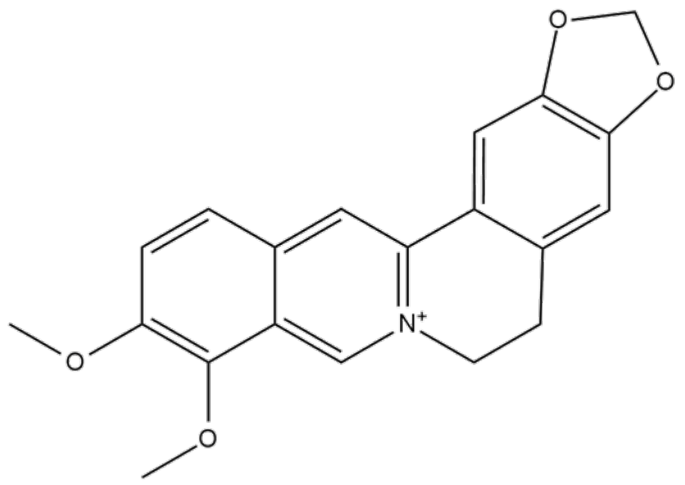	Unimolecular<br>Parallel<br>d(TGAGGGTGGGTAGGGTGGGGAA)	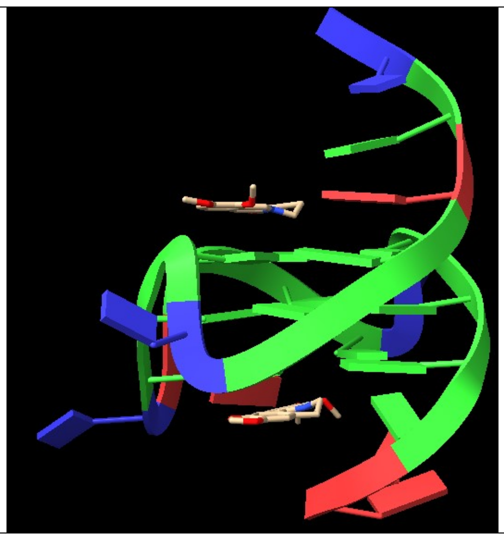	[42]
2L7V	Quindoline 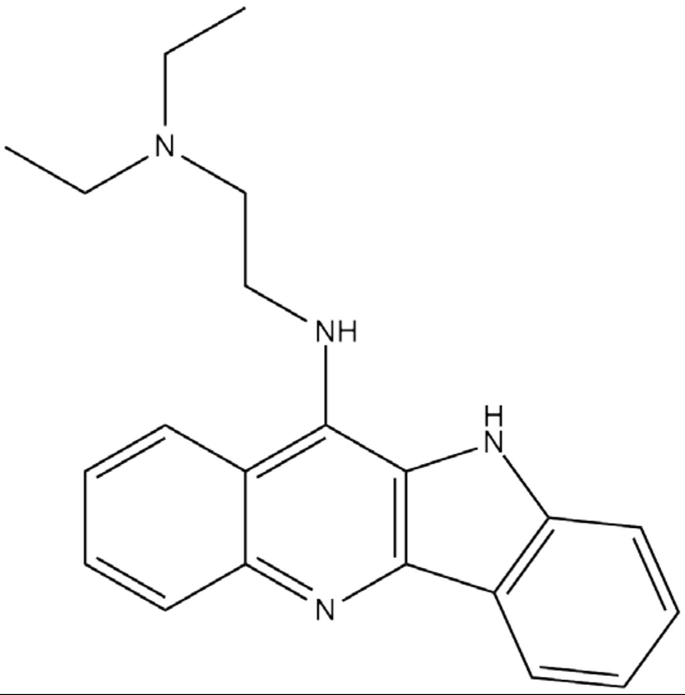	Unimolecular<br>Parallel<br>d(TGAGGGTGGGTAGGGTGGGTAA)	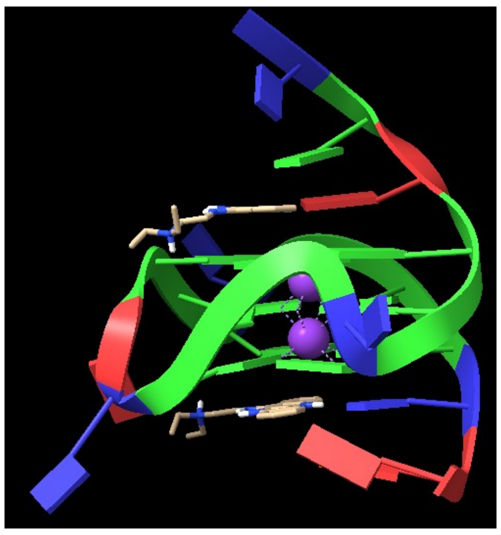	[43]
7KBW	PEQ 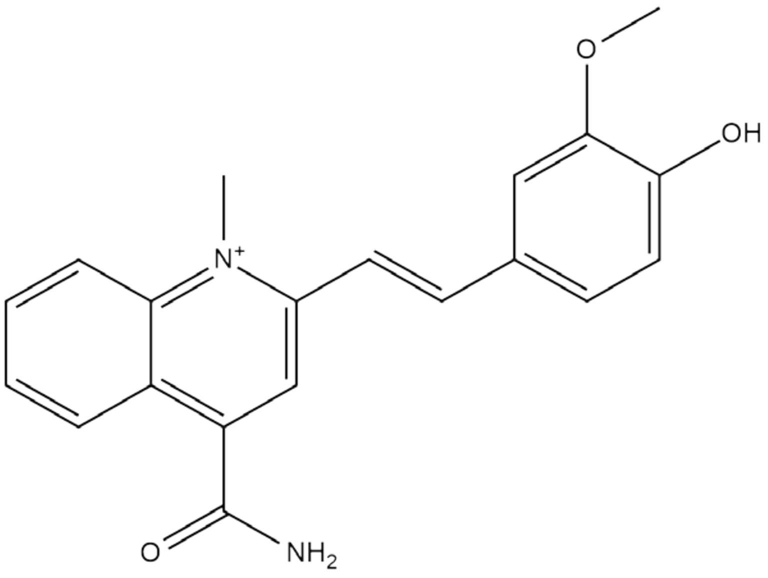	Unimolecular<br>Parallel<br>d(TGAGGGTGGGTAGGGTGGGGAA)	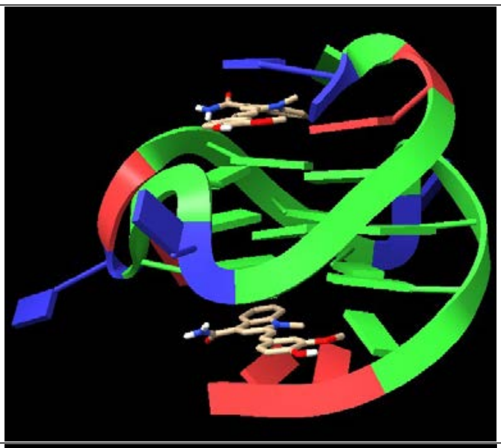	[44]
7KBX		Unimolecular<br>Parallel<br>d(TGAGGGTGGGTAGGGTGGGTAA)	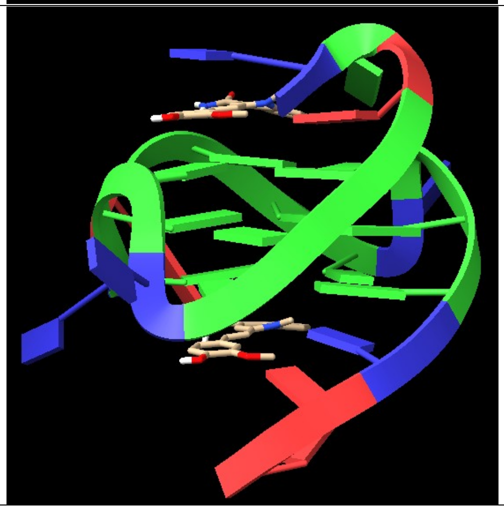	[44]
5W77	DC-34 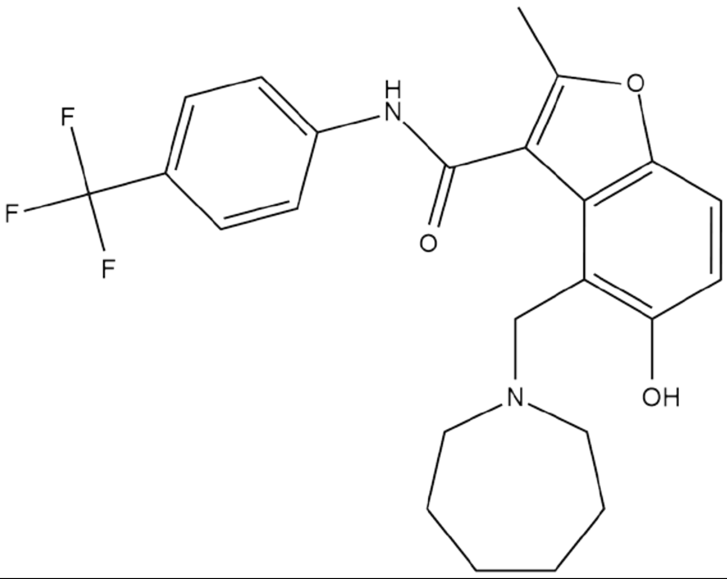	Unimolecular<br>Parallel<br>d(TGAGGGTGGGTAGGGTGGGTAA)	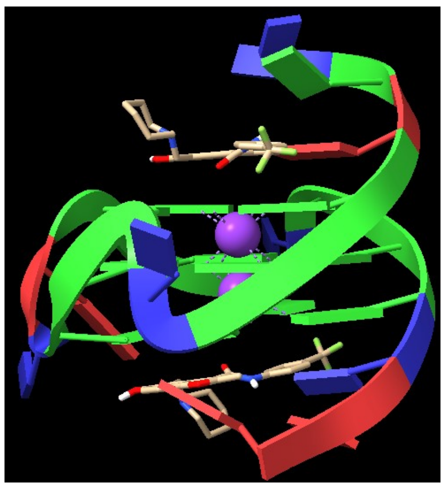	[45]

6JJ0	BMVC<br>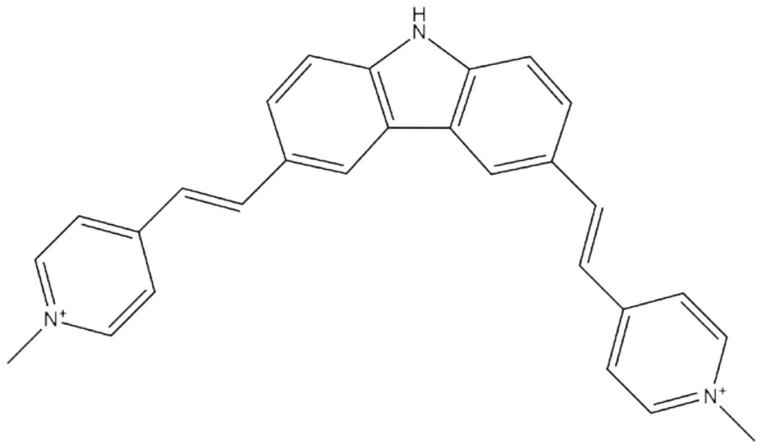	Unimolecular<br>Parallel<br>d(TGAGGGTGGGTAGGGTGGGTAA)	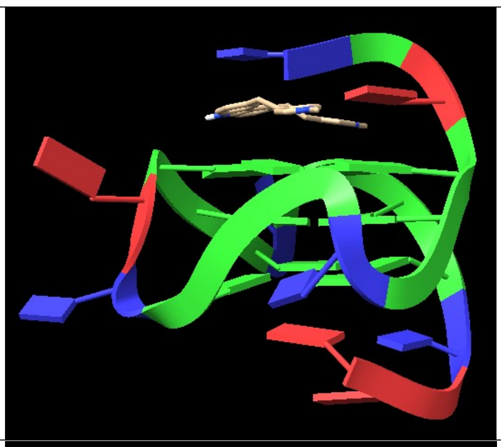	[46]
6O2L		Unimolecular<br>Parallel<br>d(TGAGGGTGGGTAGGGTGGGTAA)	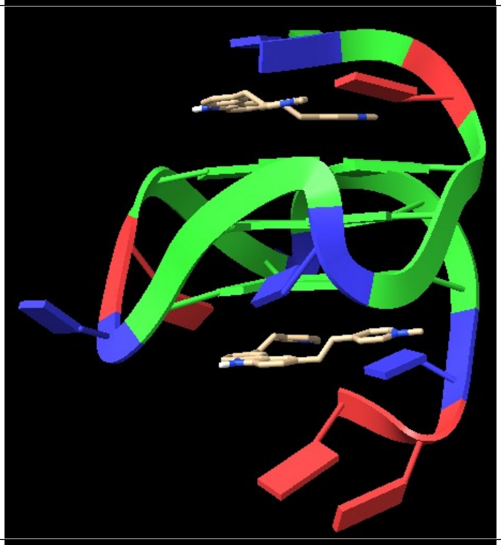	[46]
5LIG	DAOTA-M2<br>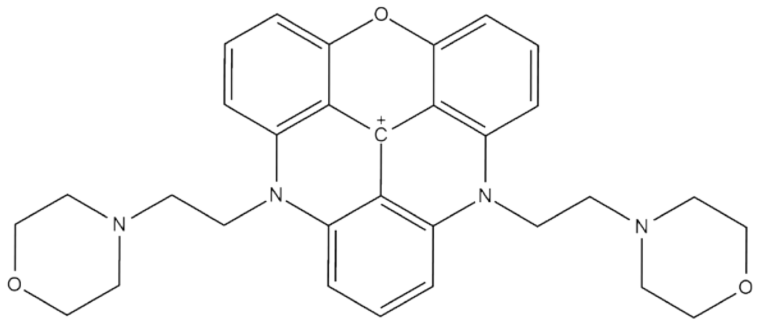	Unimolecular<br>Parallel<br>d(TAGGGAGGGTAGGGAGGGT)	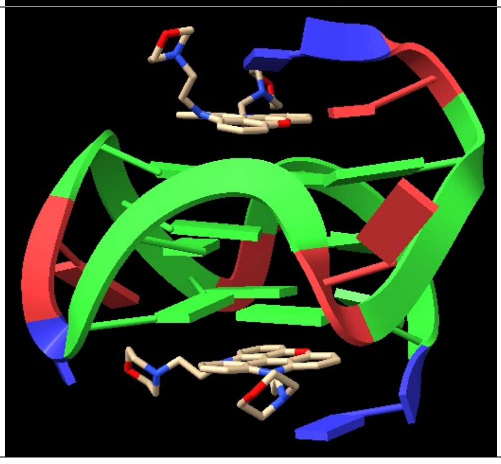	[47]
***RET* oncogene promoter G-quadruplexes**				
6JWE	Colchicine<br>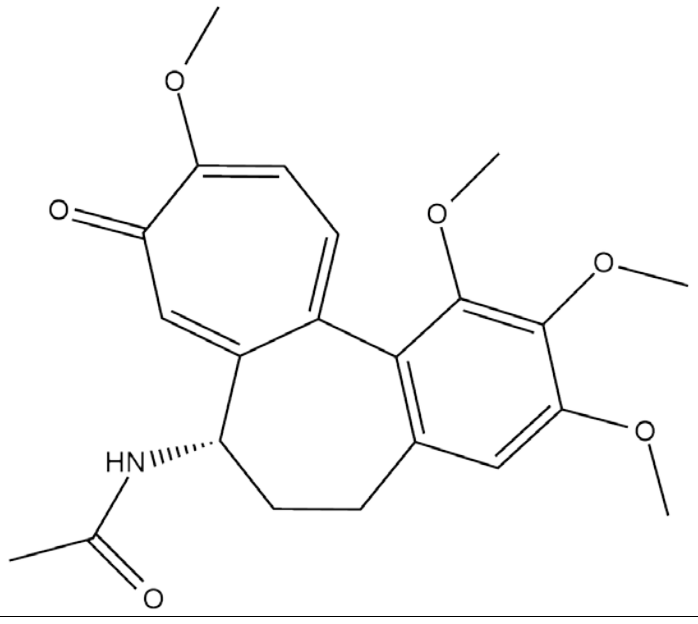	Unimolecular<br>Parallel<br>d(GGGGCGGGGCGGGGCGGGGT)	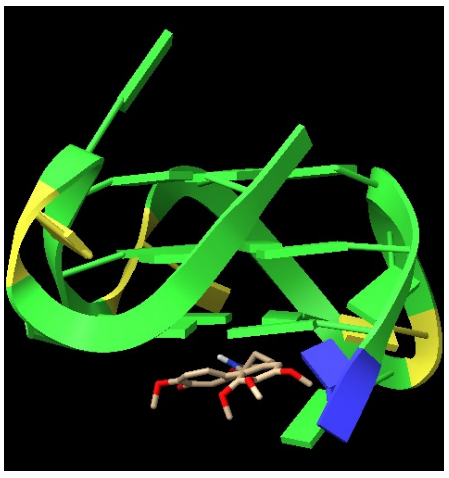	[48]
6JWD	Berberine<br>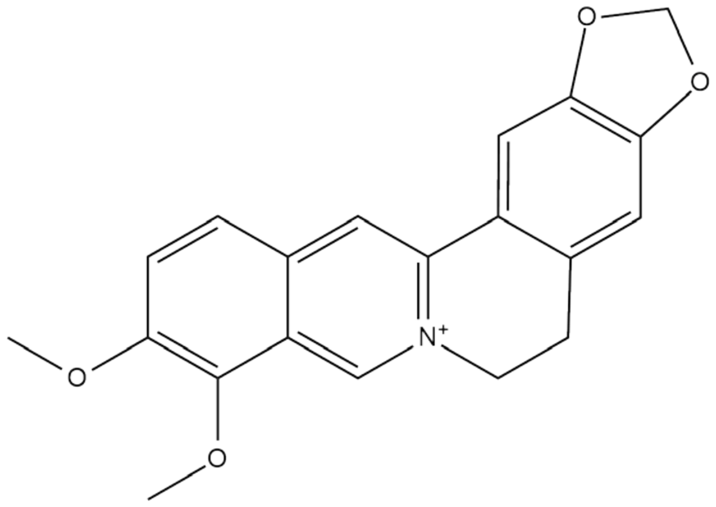	Unimolecular<br>Parallel<br>d(GGGGCGGGGCGGGGCGGGGT)	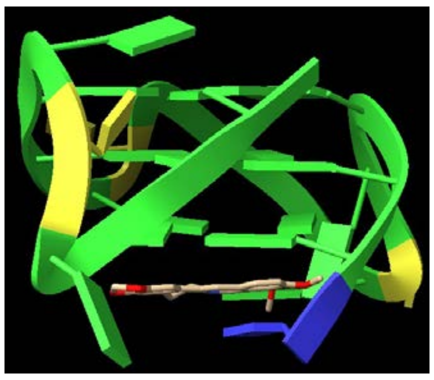	[48]
***PDGFR-β* oncogene promoter G-quadruplexes**				

6V0L	dGMP 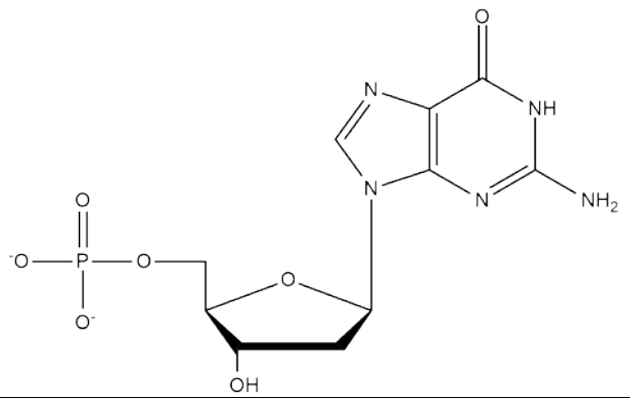	Unimolecular<br>Parallel<br>d(AAGGGAGGGCGGCGGGACA)	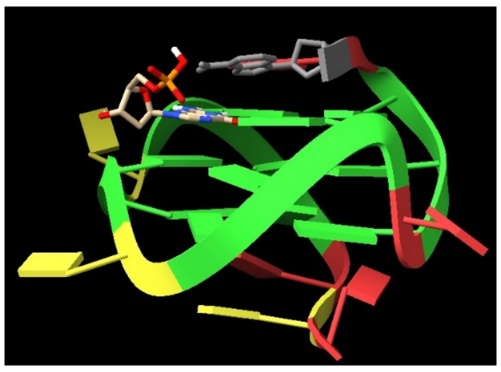	[49]
7MSV	dGMP and berberine 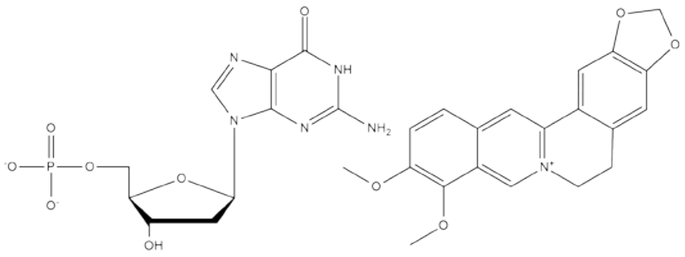	Unimolecular<br>Parallel<br>d(AAGGGAGGGCGGCGGGACA)	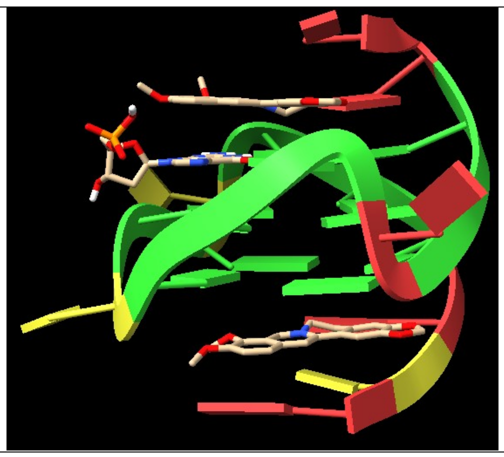	[50]
***VEGF* oncogene promoter G-quadruplexes**				
6LNZ	Pt1 to Pt2 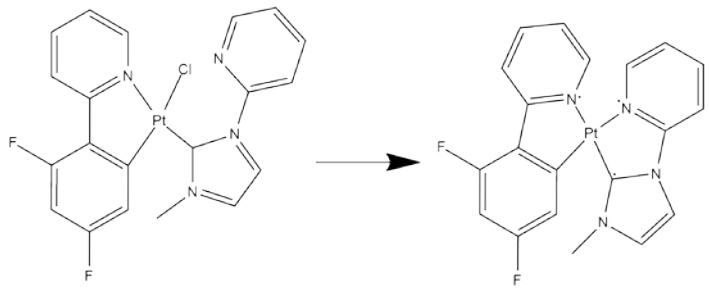	Unimolecular<br>Parallel<br>d(CGGGGCGGGCCTTGGGCGGGGT)	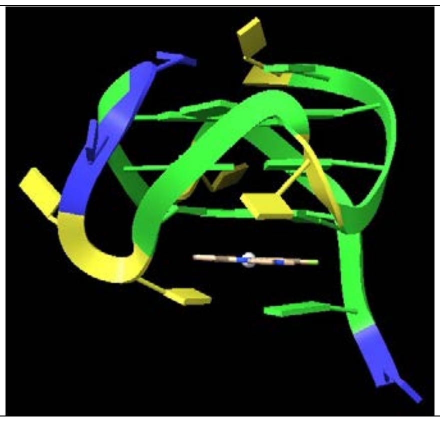	[51]
**Viral G-quadruplexes**				
6JJI	TMPyP4 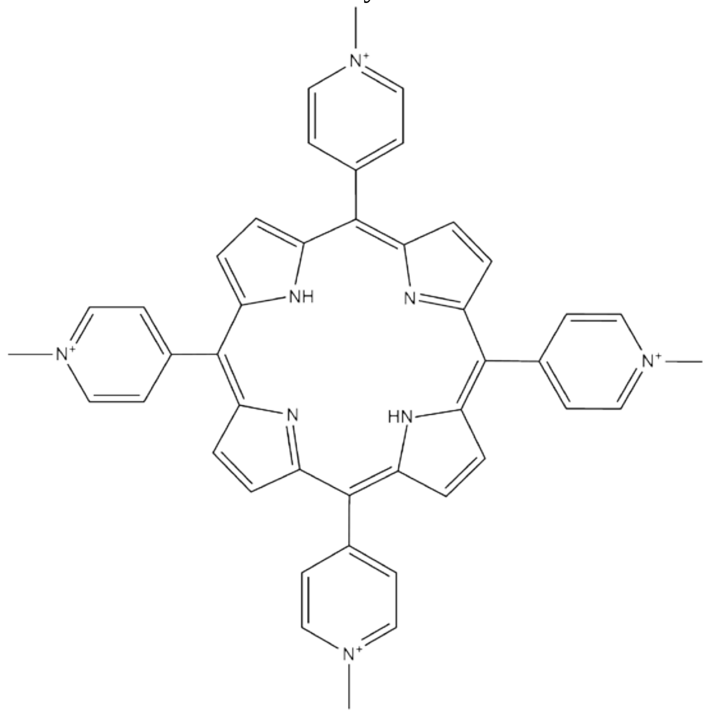	Bimolecular<br>Parallel<br>r(GGCUCGGCGGCGGA)	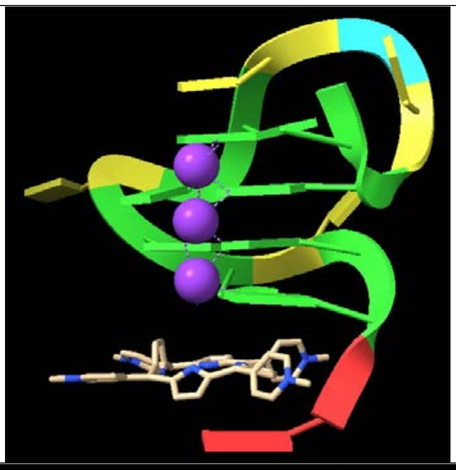	[52]
6JJH		Bimolecular<br>Parallel<br>r(GGCUCGGCGGCGGA)	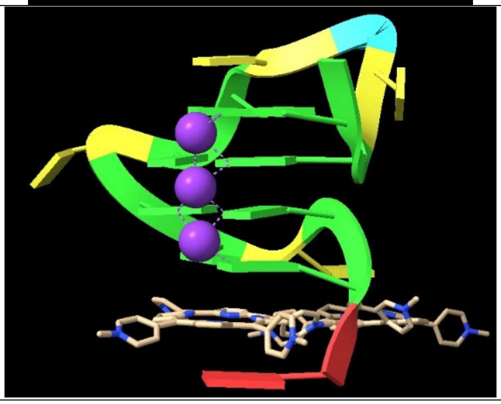	[52]

References

1. Wilson, T.; Costa, P.J.; Félix, V.; Williamson, M.P.; Thomas, J.A. Structural studies on dinuclear ruthenium(II) complexes that bind diastereoselectively to an antiparallel folded human telomere sequence. *J. Med. Chem.* **2013**, *56*, 8674–8683, doi:10.1021/jm401119b.
2. McQuaid, K.T.; Takahashi, S.; Baumgaertner, L.; Cardin, D.J.; Paterson, N.G.; Hall, J.P.; Sugimoto, N.; Cardin, C.J. Ruthenium polypyridyl complex bound to a unimolecular chair-form G-quadruplex. *J. Am. Chem. Soc.* **2022**, *144*, 5956–5964, doi:10.1021/jacs.2c00178.
3. Lin, C.; Wu, G.; Wang, K.; Onel, B.; Sakai, S.; Shao, Y.; Yang, D. Molecular recognition of the hybrid-2 human telomeric G-quadruplex by epiberberine: insights into conversion of telomeric G-quadruplex structures. *Angew. Chemie - Int. Ed.* **2018**, *57*, 10888–10893, doi:10.1002/anie.201804667.
4. Wirmer-Bartoschek, J.; Bendel, L.E.; Jonker, H.R.A.; Grün, J.T.; Papi, F.; Bazzicalupi, C.; Messori, L.; Gratteri, P.; Schwalbe, H. Solution NMR structure of a ligand/hybrid-2-G-quadruplex complex reveals rearrangements that affect ligand binding. *Angew. Chemie - Int. Ed.* **2017**, *56*, 7102–7106, doi:10.1002/anie.201702135.
5. Liu, L.Y.; Liu, W.; Wang, K.N.; Zhu, B.C.; Xia, X.Y.; Ji, L.N.; Mao, Z.W. Quantitative detection of G-quadruplex DNA in live cells based on photon counts and complex structure discrimination. *Angew. Chemie - Int. Ed.* **2020**, *59*, 9719–9726, doi:10.1002/anie.202002422.
6. Liu, W.; Zhong, Y.-F.; Liu, L.-Y.; Shen, C.-T.; Zeng, W.; Wang, F.; Yang, D.; Mao, Z.-W. Solution structures of multiple G-quadruplex complexes induced by a platinum(II)-based tripod reveal dynamic binding. *Nat. Commun.* **2018**, *9*, 3496–3506, doi:10.1038/s41467-018-05810-4.
7. Chung, W.J.; Heddi, B.; Tera, M.; Iida, K.; Nagasawa, K.; Phan, A.T. Solution structure of an intramolecular (3 + 1) human telomeric G-quadruplex bound to a telomestatin derivative. *J. Am. Chem. Soc.* **2013**, *135*, 13495–13501, doi:10.1021/ja405843r.
8. Ghosh, A.; Trajkovski, M.; Teulade-Fichou, M.; Gabelica, V.; Plavec, J. Phen-DC 3 induces refolding of human telomeric DNA into a chair-type antiparallel G-quadruplex through ligand intercalation. *Angew. Chemie Int. Ed.* **2022**, doi:10.1002/anie.202207384.
9. Bazzicalupi, C.; Ferraroni, M.; Bilia, A.R.; Scheggi, F.; Gratteri, P. The crystal structure of human telomeric DNA complexed with berberine: An interesting case of stacked ligand to G-tetrad ratio higher than 1:1. *Nucleic Acids Res.* **2013**, *41*, 632–638, doi:10.1093/nar/gks1001.
10. Bazzicalupi, C.; Ferraroni, M.; Papi, F.; Massai, L.; Bertrand, B.; Messori, L.; Gratteri, P.; Casini, A. Determinants for tight and selective binding of a medicinal dicarbene gold(I) complex to a telomeric DNA G-quadruplex: a joint ESI MS and XRD investigation. *Angew. Chemie - Int. Ed.* **2016**, *55*, 4256–4259, doi:10.1002/anie.201511999.
11. Guarra, F.; Marzo, T.; Ferraroni, M.; Papi, F.; Bazzicalupi, C.; Gratteri, P.; Pescitelli, G.; Messori, L.; Biver, T.; Gabbiani, C. Interaction of a gold(I) dicarbene anticancer drug with human telomeric DNA G-quadruplex: solution and computationally aided X-ray diffraction analysis. *Dalt. Trans.* **2018**, *47*, 16132–16138, doi:10.1039/c8dt03607a.
12. Parkinson, G.N.; Cuenca, F.; Neidle, S. Topology conservation and loop flexibility in quadruplex-drug recognition: crystal structures of inter- and intramolecular telomeric DNA quadruplex-drug complexes. *J. Mol. Biol.* **2008**, *381*, 1145–1156, doi:10.1016/j.jmb.2008.06.022.
13. Collie, G.W.; Promontorio, R.; Hampel, S.M.; Micco, M.; Neidle, S.; Parkinson, G.N. Structural basis for telomeric G-quadruplex targeting by naphthalene diimide ligands. *J. Am. Chem. Soc.* **2012**, *134*, 2723–2731, doi:10.1021/ja2102423.
14. Micco, M.; Collie, G.W.; Dale, A.G.; Ohnmacht, S.A.; Pazitna, I.; Gunaratnam, M.; Reszka, A.P.; Neidle, S. Structure-based design and evaluation of naphthalene diimide G-quadruplex ligands as telomere targeting agents in pancreatic cancer cells. *J. Med. Chem.* **2013**, *56*, 2959–2974, doi:10.1021/jm301899y.
15. Nicoludis, J.M.; Miller, S.T.; Jeffrey, P.D.; Barrett, S.P.; Rablen, P.R.; Lawton, T.J.; Yatsunyk, L.A. Optimized end-stacking provides specificity of N-methyl mesoporphyrin IX for human telomeric G-quadruplex DNA. *J. Am. Chem. Soc.* **2012**, *134*, 20446–20456, doi:10.1021/ja3088746.
16. Miron, C.E.; van Staalduinen, L.; Rangaswamy, A.M.; Chen, M.; Liang, Y.; Jia, Z.; Mergny, J.L.; Petitjean, A. Going platinum to the tune of a remarkable guanine quadruplex binder: solution- and solid-state investigations. *Angew. Chemie - Int. Ed.* **2021**, *60*, 2500–2507, doi:10.1002/anie.202012520.
17. Ferraroni, M.; Bazzicalupi, C.; Papi, F.; Fiorillo, G.; Guamán-Ortiz, L.M.; Nocentini, A.; Scovassi, A.I.; Lombardi, P.; Gratteri, P. Solution and solid-state analysis of binding of 13-substituted berberine analogues to human telomeric G-quadruplexes. *Chem. - An Asian J.* **2016**, *11*, 1107–1115, doi:10.1002/asia.201600116.
18. Papi, F.; Bazzicalupi, C.; Ferraroni, M.; Ciolli, G.; Lombardi, P.; Khan, A.Y.; Kumar, G.S.; Gratteri, P. Pyridine derivative of the natural alkaloid berberine as human telomeric G4-DNA binder: a solution and solid-state study. *ACS Med. Chem. Lett.* **2020**, *11*, 645–650, doi:10.1021/acsmedchemlett.9b00516.
19. Campbell, N.H.; Parkinson, G.N.; Reszka, A.P.; Neidle, S. Structural basis of DNA quadruplex recognition by an acridine drug. *J. Am. Chem. Soc.* **2008**, *130*, 6722–6724, doi:10.1021/ja8016973.
20. Parkinson, G.N.; Ghosh, R.; Neidle, S. Structural basis for binding of porphyrin to human telomeres. *Biochemistry* **2007**, *46*, 2390–2397, doi:10.1021/bi062244n.
21. Campbell, N.H.; Karim, N.H.A.; Parkinson, G.N.; Gunaratnam, M.; Petrucci, V.; Todd, A.K.; Vilar, R.; Neidle, S. Molecular basis of structure-activity relationships between salphen metal complexes and human telomeric DNA quadruplexes. *J. Med. Chem.* **2012**, *55*, 209–222, doi:10.1021/jm201140v.
22. McQuaid, K.; Abell, H.; Gurung, S.P.; Allan, D.R.; Winter, G.; Sorensen, T.; Cardin, D.J.; Brazier, J.A.; Cardin, C.J.; Hall, J.P. Structural studies reveal enantiospecific recognition of a DNA G-quadruplex by a ruthenium polypyridyl complex. *Angew. Chemie - Int. Ed.* **2019**, *58*, 9881–9885, doi:10.1002/anie.201814502.
23. McQuaid, K.; Hall, J.P.; Baumgaertner, L.; Cardin, D.J.; Cardin, C.J. Three thymine/adenine binding modes of the ruthenium complex Λ-[Ru(TAP)2(dppz)]$^{2+}$ to the G-quadruplex forming sequence d(TAGGGTT) shown by X-ray crystallography. *Chem. Commun.* **2019**, *55*, 9116–9119, doi:10.1039/c9cc04316k.
24. Barthwal, R.; Raje, S.; Pandav, K. Structural basis for stabilization of human telomeric G-quadruplex [d-(TTAGGGT)]$_4$ by anticancer drug epirubicin. *Bioorganic Med. Chem.* **2020**, *28*, 115761, doi:10.1016/j.bmc.2020.115761.
25. Barthwal, R.; Raje, S.; Pandav, K. Structural basis for stabilization of human telomeric G-quadruplex [d-(TTAGGGT)]$_4$ by anticancer drug adriamycin. *J. Biomol. Struct. Dyn.* **2021**, *39*, 795–815, doi:10.1080/07391102.2020.1730969.
26. Tawani, A.; Kumar, A. Structural insight into the interaction of flavonoids with human telomeric sequence. *Sci. Rep.* **2015**, *5*, 1–13, doi:10.1038/srep17574.
27. Hounsou, C.; Guittat, L.; Monchaud, D.; Jourdan, M.; Saettel, N.; Mergny, J.L.; Teulade-Fichou, M.P. G-quadruplex recognition by quinacridines: A SAR, NMR, and biological study. *ChemMedChem* **2007**, *2*, 655–666, doi:10.1002/cmdc.200600286.
28. Gavathiotis, E.; Heald, R.A.; Stevens, M.F.G.; Searle, M.S. Drug recognition and stabilisation of the parallel-stranded DNA quadruplex d(TTAGGGT)$_4$ containing the human telomeric repeat. *J. Mol. Biol.* **2003**, *334*, 25–36, doi:10.1016/j.jmb.2003.09.018.
29. Lin, L.Y.; McCarthy, S.; Powell, B.M.; Manurung, Y.; Xiang, I.M.; Dean, W.L.; Chaires, B.; Yatsunyk, L.A. Biophysical and X-ray structural studies of the (GGGTT)$_3$GGG G-quadruplex in complex with N-methyl mesoporphyrin IX. *PLoS One* **2020**, *15*, 1–23, doi:10.1371/journal.pone.0241513.
30. Winnerdy, F.R.; Das, P.; Heddi, B.; Phan, A.T. Solution structures of a G-quadruplex bound to linear- and cyclic-dinucleotides. *J. Am. Chem. Soc.* **2019**, *141*, 18038–18047, doi:10.1021/jacs.9b05642.
31. Haider, S.M.; Parkinson, G.N.; Neidle, S. Structure of a G-quadruplex-ligand complex. *J. Mol. Biol.* **2003**, *326*, 117–125, doi:10.1016/S0022-2836(02)01354-2.
32. Campbell, N.H.; Patel, M.; Tofa, A.B.; Ghosh, R.; Parkinson, G.N.; Neidle, S. Selectivity in ligand recognition of G-quadruplex loops. *Biochemistry* **2009**, *48*, 1675–1680, doi:10.1021/bi802233v.
33. Campbell, N.H.; Smith, D.L.; Reszka, A.P.; Neidle, S.; O'Hagan, D. Fluorine in medicinal chemistry: β-fluorination of peripheral pyrrolidines attached to acridine ligands affects their interactions with G-quadruplex DNA. *Org. Biomol. Chem.* **2011**, *9*, 1328–1331, doi:10.1039/c0ob00886a.
34. Mandal, P.K.; Baptiste, B.; Langlois d'Estaintot, B.; Kauffmann, B.; Huc, I. Multivalent interactions between an aromatic helical foldamer and a DNA G-quadruplex in the solid state. *ChemBioChem* **2016**, *17*, 1911–1914, doi:10.1002/cbic.201600281.
35. Clark, G.R.; Pytel, P.D.; Squire, C.J.; Neidle, S. Structure of the first parallel DNA quadruplex-drug complex. *J. Am. Chem. Soc.* **2003**, *125*, 4066–4067, doi:10.1021/ja0297988.
36. Clark, G.R.; Pytel, P.D.; Squire, C.J. The high-resolution crystal structure of a parallel intermolecular DNA G-4 quadruplex/drug complex employing syn glycosyl linkages. *Nucleic Acids Res.* **2012**, *40*, 5731–5738, doi:10.1093/nar/gks193.
37. Martino, L.; Virno, A.; Pagano, B.; Virgilio, A.; Di Micco, S.; Galeone, A.; Giancola, C.; Bifulco, G.; Mayol, L.; Randazzo, A. Structural and thermodynamic studies of the interaction of distamycin A with the parallel quadruplex structure [d(TGGGGT)]$_4$. *J. Am. Chem. Soc.* **2007**, *129*, 16048–16056, doi:10.1021/ja075710k.
38. Cosconati, S.; Marinelli, L.; Trotta, R.; Virno, A.; De Tito, S.; Romagnoli, R.; Pagano, B.; Limongelli, V.; Giancola, C.; Baraldi, P.G.; et al. Structural and

conformational requisites in DNA quadruplex groove binding: Another piece to the puzzle. *J. Am. Chem. Soc.* **2010**, *132*, 6425–6433, doi:10.1021/ja1003872.
39. Collie, G.W.; Sparapani, S.; Parkinson, G.N.; Neidle, S. Structural basis of telomeric RNA quadruplex-acridine ligand recognition. *J. Am. Chem. Soc.* **2011**, *133*, 2721–2728, doi:10.1021/ja109767y.
40. Chung, W.J.; Heddi, B.; Hamon, F.; Teulade-Fichou, M.P.; Phan, A.T. Solution structure of a G-quadruplex bound to the bisquinolinium compound phen-DC3. *Angew. Chemie - Int. Ed.* **2014**, *53*, 999–1002, doi:10.1002/anie.201308063.
41. Phan, A.T.; Kuryavyi, V.; Gaw, H.Y.; Patel, D.J. Small-molecule interaction with a five-guanine-tract G-quadruplex structure from the human myc promoter. *Nat. Chem. Biol.* **2005**, *1*, 167–173, doi:10.1038/nchembio723.
42. Dickerhoff, J.; Brundridge, N.; McLuckey, S.A.; Yang, D. Berberine molecular recognition of the parallel MYC G-quadruplex in solution. *J. Med. Chem.* **2021**, *64*, 16205–16212, doi:10.1021/acs.jmedchem.1c01508.
43. Dai, J.; Carver, M.; Hurley, L.H.; Yang, D. Solution structure of a 2:1 quindoline-c-MYC G-quadruplex: Insights into G-quadruplex-interactive small molecule drug design. *J. Am. Chem. Soc.* **2011**, *133*, 17673–17680, doi:10.1021/ja205646q.
44. Dickerhoff, J.; Dai, J.; Yang, D. Structural recognition of the MYC promoter G-quadruplex by a quinoline derivative: Insights into molecular targeting of parallel G-quadruplexes. *Nucleic Acids Res.* **2021**, *49*, 5905–5915, doi:10.1093/nar/gkab330.
45. Calabrese, D.R.; Chen, X.; Leon, E.C.; Gaikwad, S.M.; Phyo, Z.; Hewitt, W.M.; Alden, S.; Hilimire, T.A.; He, F.; Michalowski, A.M.; et al. Chemical and structural studies provide a mechanistic basis for recognition of the MYC G-quadruplex. *Nat. Commun.* **2018**, *9*, 1–15, doi:10.1038/s41467-018-06315-w.
46. Liu, W.; Lin, C.; Wu, G.; Dai, J.; Chang, T.C.; Yang, D. Structures of 1:1 and 2:1 complexes of BMVC and MYC promoter G-quadruplex reveal a mechanism of ligand conformation adjustment for G4-recognition. *Nucleic Acids Res.* **2019**, *47*, 11931–11942, doi:10.1093/nar/gkz1015.
47. Kotar, A.; Wang, B.; Shivalingam, A.; Gonzalez-Garcia, J.; Vilar, R.; Plavec, J. NMR structure of a triangulenium-based long-lived fluorescence probe bound to a G-quadruplex. *Angew. Chemie - Int. Ed.* **2016**, *55*, 12508–12511, doi:10.1002/anie.201606877.
48. Wang, F.; Wang, C.; Liu, Y.; Lan, W.; Han, H.; Wang, R.; Huang, S.; Cao, C. Colchicine selective interaction with oncogene: RET G-quadruplex revealed by NMR. *Chem. Commun.* **2020**, *56*, 2099–2102, doi:10.1039/d0cc00221f.
49. Wang, K.-B.; Dickerhoff, J.; Wu, G.; Yang, D. PDGFR-β promoter forms a vacancy G-quadruplex that can be filled in by dGMP: solution structure and molecular recognition of guanine metabolites and drugs. *J. Am. Chem. Soc.* **2020**, *142*, 5204–5211, doi:10.1021/jacs.9b12770.
50. Wang, K.B.; Dickerhoff, J.; Yang, D. Solution structure of ternary complex of berberine bound to a dGMP-fill-in vacancy G-quadruplex formed in the PDGFR-β promoter. *J. Am. Chem. Soc.* **2021**, *143*, 16549–16555, doi:10.1021/jacs.1c06200.
51. Zhu, B.C.; He, J.; Liu, W.; Xia, X.Y.; Liu, L.Y.; Liang, B.B.; Yao, H.G.; Liu, B.; Ji, L.N.; Mao, Z.W. Selectivity and targeting of G-quadruplex binders activated by adaptive binding and controlled by chemical kinetics. *Angew. Chemie - Int. Ed.* **2021**, *60*, 15340–15343, doi:10.1002/anie.202104624.
52. Zhang, Y.; El Omari, K.; Duman, R.; Liu, S.; Haider, S.; Wagner, A.; Parkinson, G.N.; Wei, D. Native de novo structural determinations of non-canonical nucleic acid motifs by X-ray crystallography at long wavelengths. *Nucleic Acids Res.* **2020**, *48*, 9886–9898, doi:10.1093/nar/gkaa439.
